# Supplementary material for: 2D Short-Time Fourier Transform for local morphological analysis of meibomian gland images
Source: PLoS One. 2022 Jun 24;17(6):e0270473. doi: 10.1371/journal.pone.0270473 (PMC9491703; doi:10.1371/journal.pone.0270473)
Supplement: S1 Appendix — (PDF) [file pone.0270473.s001.pdf]

S1. Meibomian gland images acquisition.

Meibomian gland imaging was performed using the home-built non-invasive meibography system composed of a ring-shaped IR LED illumination panel and a Imaging Source DMK72AUC02 8bit CMOS Camera with an IR low-pass filter (LP 720 nm) fixed in the LED ring centre. The images were acquired with a resolution of 20pix/mm. The device was mounted on the Topcon SL-D701 slit lamp and allows to record meibographic images of upper and lower eyelids in routine examination. The design model of the device and its physical implementation are shown in the Fig.1. An example photo recorded with this device is shown in Fig. 2.

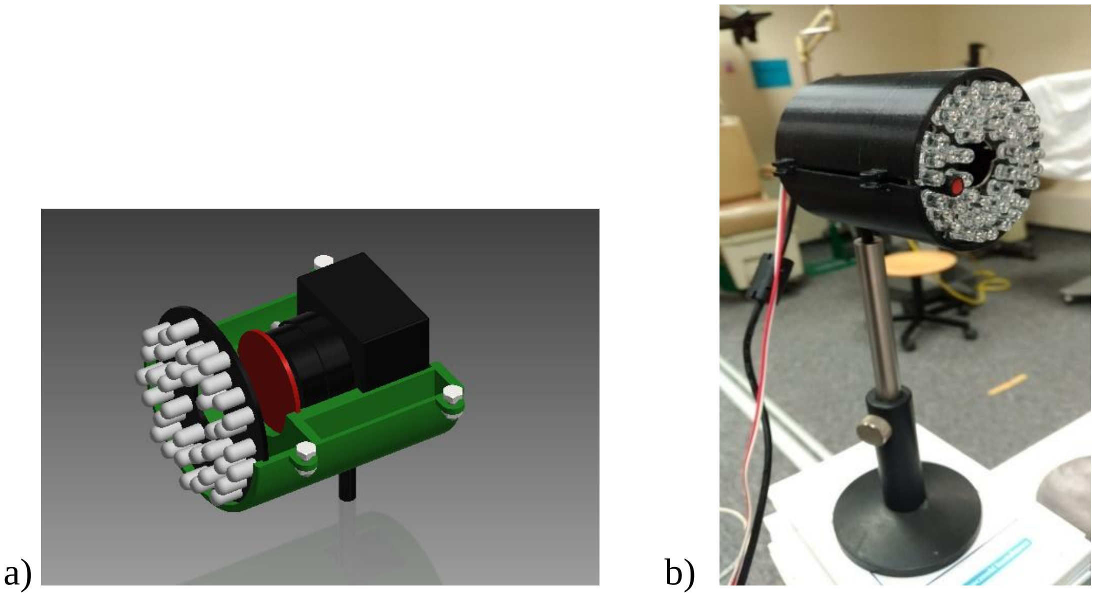

Fig.1 Home-built non-invasive meibography system. a) Design of the device. The source of infrared light consists of LEDs arranged on the ring (white cylinders). The low-pass infrared light filter (red disc) is placed in front of the CCD camera with an objective lens (black box). All elements are placed in the cover (green). b) image of the real device.

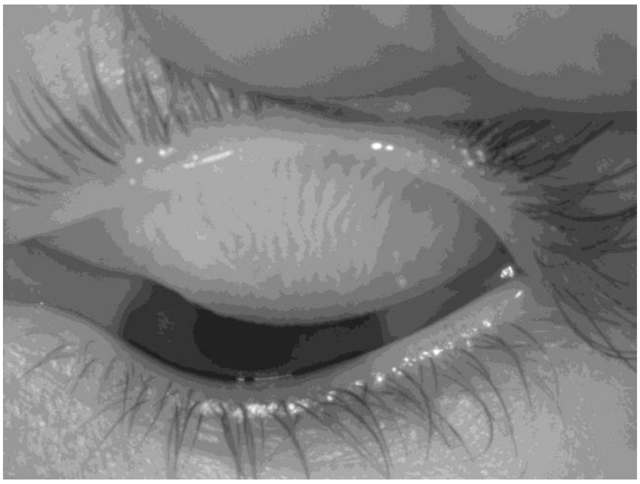

Fig.2 Example photo recorded with home-built meibography system shown in Fig.1
