# Supplementary material for: 2D Short-Time Fourier Transform for local morphological analysis of meibomian gland images
Source: PLoS One. 2022 Jun 24;17(6):e0270473. doi: 10.1371/journal.pone.0270473 (PMC9491703; doi:10.1371/journal.pone.0270473)
Supplement: S2 Appendix — (PDF) [file pone.0270473.s002.pdf]

S1. 2D Short-Time Fourier Transform

2D Fourier transformation of images together with all subsequent image manipulation was performed using NI LabVIEW® with NI Vision module.

2D Short-Time Fourier Transform consist of calculation of 2D discrete Fourier transform for selected region of the image. Selection is made by multiplying original image *f* by an image of a window *w*. The window can be placed in any position given by pixels (x_w, y_w). 2D discrete Fourier transform is calculated according to:

$$F_w(q_x,q_y) = \frac{1}{N^2}\sum_{q_x=0}^{N-1}\sum_{q_y=0}^{N-1} w(x-x_w, y-y_w) f(x,y) e^{-i\frac{2\pi}{N}(q_x x + q_y y)} \quad \text{(S1)}$$

where $f(x,y)$ is the pretreated Meibomian image, N is the number of pixels in x and y direction, q_x and q_y are new coordinates corresponding to spatial frequencies in direction x and y, respectively.

An image of a Gaussian window with width (standard deviation) σ_r and with center positon x_w, y_w was calculated using

$$w(x-x_w, y-y_w) = \exp\left(-\frac{r(x-x_w, y-y_w)^2}{2\sigma_r^2}\right) \quad \text{(S2)}$$

where r is an radial distance from Gaussian maximum given by

$$r(x-x_w, y-y_w) = \sqrt{(x-x_w)^2 + (y-y_w)^2} \quad \text{(S3)}$$

Before calculation of 2D Fourier transform of a windowed image ($w(x-x_w, y-y_w) f(x,y)$), a DC content of an image was removed by subtracting from each pixel of windowed image an average of its pixel values: $w \cdot f$ – avrg($w \cdot f$).

Eq.S1 provides a NxN matrix of complex numbers from which a Power Spectrum Density (PSD) was calculated

$$PSD(q_x,q_y) = \left|F(q_x,q_y)\right|^2 \quad \text{(S4)}$$

PSD calculated from eq. S1 was transformed from cartesian to polar coordinate system

$$F(q_x,q_y) \rightarrow F(q,\theta) \quad \text{(S5)}$$

$$\begin{aligned} q_x &= q\cos(\theta) \\ q_y &= q\sin(\theta) \end{aligned} \quad \text{(S6)}$$

The $F(q,\theta)$ was normalized to obtain probability density function $p(q,\theta)$

$$p(q,\theta) = \frac{|\mathrm{F}(q,\theta)|^2}{\iint |\mathrm{F}(q,\theta)|^2\, dq\, d\theta}\ , \quad \text{(S7)}$$

from which marginal density function $p(q)$ and $p(\theta)$ were calculated.

$$p(q) = \int p(q,\theta) d\theta \quad \text{(S8)}$$

$$p(\theta) = \int p(q,\theta) dq \quad \text{(S9)}$$
