# Supplementary material for: 2D Short-Time Fourier Transform for local morphological analysis of meibomian gland images
Source: PLoS One. 2022 Jun 24;17(6):e0270473. doi: 10.1371/journal.pone.0270473 (PMC9491703; doi:10.1371/journal.pone.0270473)
Supplement: S3 Appendix — (PDF) [file pone.0270473.s003.pdf]

S2. The consequence of finite width of Gaussian window

As follows from eq.S7-S9 Power Spectrum Density, PSD ($|F(q,\theta)|^2$), obtained by 2D Fourier transform of an image provides marginal density functions, $p(q)$ and $p(\theta)$, which inform on the probability of finding in analyzed image a sinusoidal pattern of certain spatial frequency and certain orientation, respectively. If analyzed image is the simplest stationary (unchanging) and unlimited (unwindowed) sinusoidal pattern (fig.S1 a1), these probability density functions take the form of the Dirac's delta function, which is a peak specified only for a single value (row 1 of fig.S1). In other words, the position of delta peak unambiguously indicates the frequency and orientation of the sinusoidal signal. If, on the other hand, the analyzed image is still unlimited but not stationary (the frequency or the orientation of pattern changes within image) then $p(q)$ and/or $p(\theta)$ probability distribution are characterized by certain width. The finite width of marginal distributions informs on the distortion of the gland structure. This was the situation we faced in our recent paper [1] where we analyzed unlimited image (whole image) of Meibomian glands (which are not stationary). One of the descriptive features extracted was width (variance) of orientation probability distribution, which informs about anisotropy in gland periodicity.

In current work we wish to derive the same information but on the local scale. To achieve this we performed series of 2D Fourier transforms on the limited regions of the Meibomian image indicated by positions of a window. Because within each of such window, Meibomian glands can change their shape, we deal here with Fourier analysis of non-stationary and limited signal.

The most important consequence of the signal limitation (windowing), is that its PSD (and so the probability distributions) is not described by a delta function anymore. PSD of an ideal image of a perfect sinusoidal pattern being limited by a window shows a peak of finite shape which depends on the particular window shape used to limit the analyzed image.

In our analysis we used a Gaussian shape of a window. The width of the window is defined by its variance (standard deviation), σ_r. Utilization of Gaussian shape has a convenient property that Fourier transformation preserve its Gaussian shape. In other words, the shape of the PSD peak is also Gaussian.

On fig. S1 we present the effect of image limitation on resulting PSD (both in Cartesian and polar coordinates). As an image we used the simples stationary sinusoidal signal oriented at $\pi/4$ rad (45°) . This is the simplest model of ideal Meibomian gland structure without any deviation in their width nor their orientations. We present the effect of Gaussian window variance as well the influence of the frequency of the signal (corresponding to the width of Meibomian glands) on resulting PSD's. First row (row 1) shows the result for unlimited image, row 2) is for the image limited by a broad Gaussian window, row 3) is for the image limited by a narrow Gaussian window, whereas row 4) is for higher frequency image (thinner glands) limited by a narrow Gaussian window. Column a) in Fig.S1 shows the analyzed image, column b) indicate the PSD of an image in Cartesian coordinates, whereas column c) shows PSD in polar coordinates.

As follows from first row of Fig.S1, PSD's of unlimited sinusoidal pattern shows extremely narrow spectral features. The situation changes when this image is limited by a Gaussian window of a finite variance, σ_r. It is clear how this translate to broadening of PSD spectral features (quantifies by its variance σ_q). This is even more enhanced when the Gaussian window becomes even narrower, which is illustrated on the third row of Fig.S1. This shows a common behavior connected with Fourier transform of a limited signal. The more localization we impose on the signal (the lower σ_r), the more distributed are their frequency components (the higher σ_q). It is also worth to indicate that when Gaussian limitation is imposed on the sinusoidal of the same frequency (first 3 rows in Fig.S1), PSD peaks position does not change. What is

changing is only width of those peaks (σ_q). It is also worth to notice the difference in the shape of the Gaussian peak observed in PSD depending on the coordination system used. In Cartesian coordinated PSD the peak is symmetric, which is not in general the case for PSD expressed in polar coordinates (compare columns b and c of fig.S1). This last observation is important because (as follows from eq.S7-S9) polar representation of PSD was used to calculate the probability distributions.

The situation complicates when the frequency of the windowed signal changes (the glands width changes). If the signal's frequency increases, then the peaks observed in Cartesian PSD moves to higher frequencies (moves away from the center of PSD image indicating zero frequency). The width of the peak in Cartesian PSD (σ_q) does not depend on the signals frequency (only on variance σ_r). This can be observed comparing fig.S1 3b and 4b. However, the frequency of the signal has a distinct consequences when PSD is presented in polar coordinates (compare fig.S1 3c and 4c). Although in polar representation the variance in the *q*-direction, σ_q, does not depend on signal's frequency, the variance in the θ-direction, σ_θ, strongly depends on the frequency of the signal.

The value of this ideal variance of the $p(q)$ distribution obtained when ideal sinusoidal signal (corresponding to undisturbed Meibomian glands) is windowed by a Gaussian can be calculated as

$$\sigma_{q,id} = \frac{\sqrt{2}}{4\pi\sigma_r}, \tag{S10}$$

where σ_r is the variance of Gaussian window.

Similarly, the value of ideal variance of the $p(\theta)$ distribution can be calculated as

$$\sigma_{\theta,id} = \mathrm{atan}\left(\frac{\sigma_{q,id}}{q_0}\right), \tag{S11}$$

where q_0 is mean value of $p(q)$ distribution given by eq.S12 and $\sigma_{\theta,\mathrm{id}}$ is ideal variance of this distribution given by eq.S10.

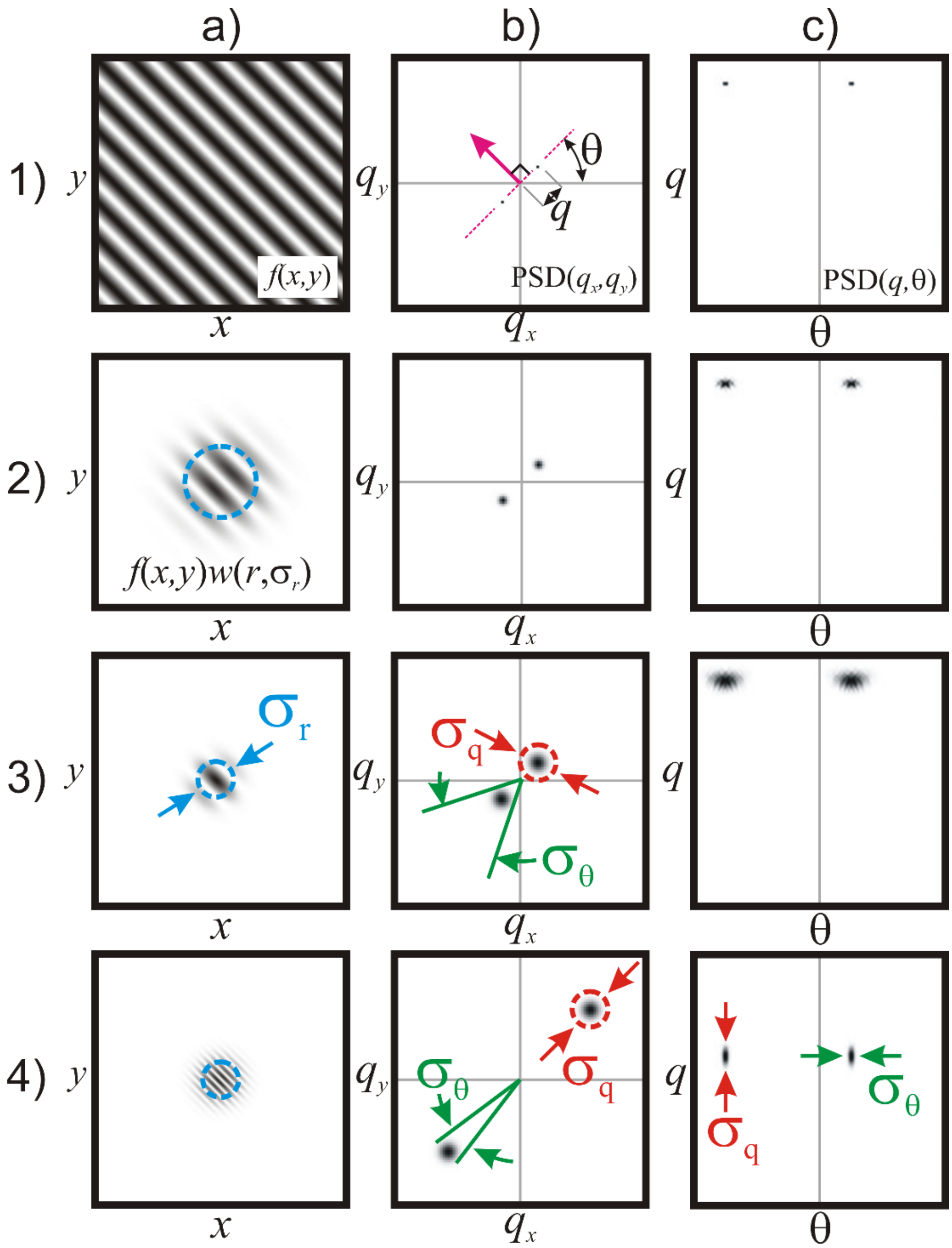

Fig.S1 The consequence of finite variance, σ_r, of Gaussian window, imposed on the image of perfect sinusoidal pattern, on the Power Spectral Density (PSD) in Cartesian and polar coordinates. Row 1) unlimited image of low frequency sinusoid; row 2) low frequency sinusoid image limited by a Gaussian window of high σ_r; row 3) low frequency sinusoid image limited by a Gaussian window of low σ_r; row 4) high frequency sinusoid image limited by a Gaussian window of low σ_r. Column a) an image; column b) PSD in Cartesian coordinates; column c) PSD in polar coordinates. Notice that for unlimited signal (row 1) both PSD's shows extremely narrow peaks. Definition of polar coordinates (q,θ) are shown in panel 1b). When the signal is limited by a Gaussian window (rows 2-4), the width of PSD's distributions become finite and depends on the variance of the Gaussian window (the broader the window, the narrower the PSD peak). Notice, that in Cartesian PSD (column b) the shape of the peak is symmetric with the same variance in both q_x and q_y directions equal to σ_q. In polar coordinate the shape of the PSD distribution is generally non-symmetric with variance in q and θ direction equal to σ_q and σ_θ, respectively. Notice that the higher the frequency of the signal (the further away the peak is from the Cartesian origin) the narrower it get in polar coordinates (panels 3c and 4c). This is explained in panels 3b) and 4b) where the peak of constant variance σ_q covers a greater range of angles σ_θ when moved further from Cartesian origin.

On fig.S2 the influence of Gaussian window variance and of frequency of sinusoidal signal on the $p(q)$ and $p(\theta)$ probability distribution are presented in more detail. Panel a) shows the

cross-section of 2D Gaussian window differing in variance σ_r. Green curve correspond to unlimited situation where the window's width is infinite. In panel b) an angular probability distributions $p(\theta)$ are presented for windows from panel a). Notice that for a constant frequency of signal (solid lines), increase in window's variance, σ_r, results in decrease of the variance of $p(\theta)$, σ_θ. For a window of a given variance, increase in signal's frequency results in narrower $p(\theta)$ distribution (lower σ_θ) (compare solid blue with dashed blue lines in panel b). The functional form relating σ_θ with σ_q and σ_r is given be eq.S10 and eq.S11. As sinusoidal image pattern was always oriented at 45° with respect to image x-axis, all $p(\theta)$ distributions are centered around $\theta=\pi/4$ (45°). In panel c) frequency probability distributions $p(q)$ are presented for windows from panel a). Notice inverse proportionality between σ_r and σ_q expressed in eq.S10. Also notice that the variance of $p(q)$, σ_q, does not depend on the mean of this distribution (compare solid blue with dashed blue lines in panel c).

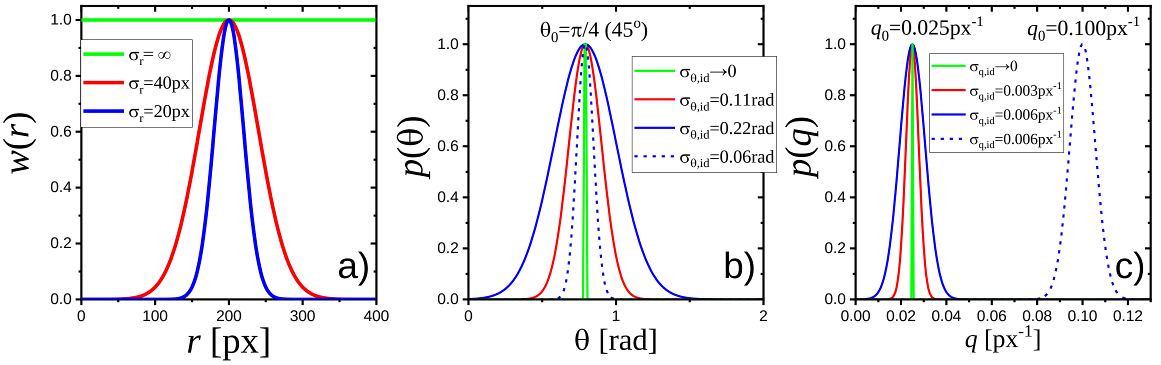

Fig.S2 Influence of Gaussian window variance on the $p(\theta)$ and $p(q)$ probability distributions. Solid lines in panels b) and c) are calculated for low frequency signal (q_0=0.025px^{-1}) limited by windows of different width, whereas dashed blue line is for signal of higher frequency (q_0=0.1px^{-1}) limited by narrow window (σ_r =20px).

To conclude, if we decide to use a local analysis using the Gaussian window limitation, then the width (variance) of the probability distribution is not a unambiguous measure of gland deformation (as was the case with the global analysis). The $p(\theta)$ and $p(q)$ probability distributions have finite variances already for ideal sinusoids (perfect glands).

Unlike in above ideal situation, in real images of Meibomian glands, their frequency and/or orientation are never constant. As a result, measured variances of the $p(q)$ and/or $p(\theta)$ probability distributions for real gland images deviates from their ideal (expected for perfect sinusoid) values. The real value of probability distribution variance, unaffected by the presence of window, was then derived from a difference between measured and ideal values of variances of both p(q) and p(θ) distributions. These were named: the gland orientation variance and the gland frequency variance, respectively (detailed definitions are given below).

The choice of Gaussian window introduces additional parameter, namely its width (given by standard deviation of an Gaussian, σ_r). The window width has to be wide enough to embrace the gland structure, but narrow enough to ensure locality (window covering whole Meibomian image will result in global analysis). As a rule of thumb, the width of the window should be slightly greater than the mean gland period (gland-gland distance). For our imaging system the mean Meibomian gland period was around 30px which justifies the full width of Gaussian filer $2\sigma_r$ =40pix used in our analysis.

[1] Ciężar, K., Pochylski, M. 2D fourier transform for global analysis and classification of meibomian gland images. Ocul Surf 2020, 18(4), 865-870. https://doi.org/10.1016/j.jtos.2020.09.005.
