# Supplementary material for: 2D Short-Time Fourier Transform for local morphological analysis of meibomian gland images
Source: PLoS One. 2022 Jun 24;17(6):e0270473. doi: 10.1371/journal.pone.0270473 (PMC9491703; doi:10.1371/journal.pone.0270473)
Supplement: S4 Appendix — (PDF) [file pone.0270473.s004.pdf]

S3. Calculation of intrinsic images

Marginal density functions $p(q)$ and $p(\theta)$ describe probability that, in selected (by a window) region of Meibomian image, there exist a periodic structure of a given frequency q and angle θ. Fig.S3 illustrates how the shapes of probability distributions depend on the window position. Open symbols in Fig.S3 correspond to two arbitrary window positions (w_1 and w_2) shown in Fig.1a in the main manuscript.

The $p(q)$ and $p(\theta)$ distributions can be fitted to appropriate theoretical distributions in order to obtain numerical values of measures of distributions. Collecting these values for all analyzed regions of Meibomian image (for all window positions (x_w, y_w)) in form of a matrix allows to create images of these parameters.

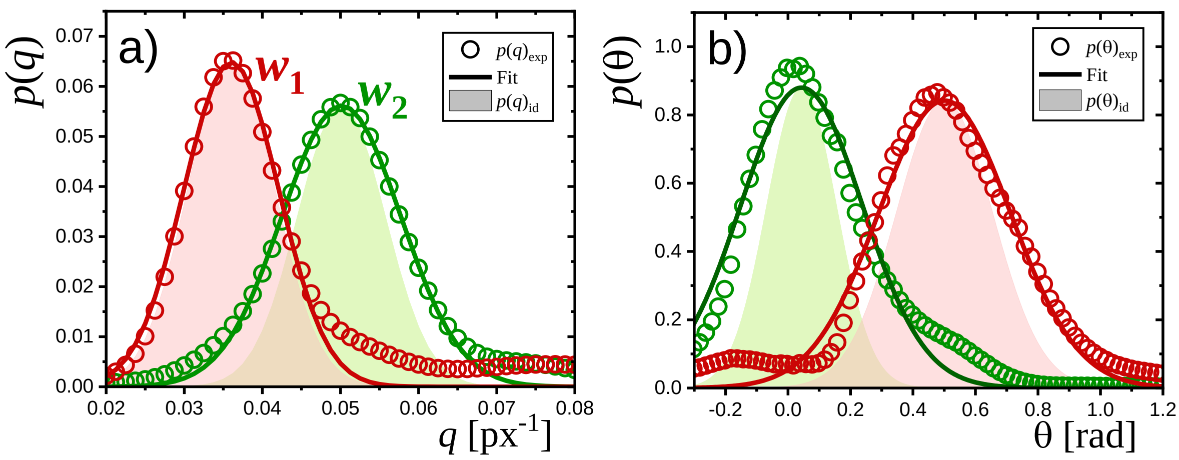

Fig.S3 Comparison of $p(q)$ (panel a) and $p(\theta)$ (panel b) distributions for two different window positions (w_1 and w_2 in Fig.1). Notice that depending on the window position, the distribution maximum and the variances of both distributions changes. Plotting the values of those parameters for all window positions produce maps being intrinsic images. Open symbols are experimentally determined probability distributions. Solid lines are the result of fitting with proper theoretical model (eq. S.12 and S.15). Shaded areas are ideal distributions expected for undisturbed gland structure. The width of these ideal distributions are given by eq.S.10 and S.11.

Image of gland frequency, q_0

The value of gland frequency, q_0, was obtain by comparing the experimental frequency density function $p(q)$ (eq.S8) with the normal distribution

$$p(q) = \frac{1}{\sigma_{q,fit}\sqrt{2\pi}} \exp\left(\frac{-(q-q_0)^2}{2\sigma_{q,fit}^2} \right) \qquad \text{(S12)}$$

where $\sigma_{q,fit}^2$ is a variance, whereas q_0 is a mean of the distribution.

Collecting q_0 values for all window position results in an image of frequency, $q_0(x_w, y_w)$.

Image of gland frequency variance, σ_q

The value of variance of experimental angular density function, $\sigma_{q,fit}$, can be found from fit with eq.S12. However, the fact that the Gaussian window used in 2D STFT analysis has finite width is reflected in finite width of the PSD (and so in $p(q)$ distribution). As a result, even an ideal single frequency periodic feature (for which no variance in $p(q)$ distribution

should be expected) will be described by finite $p(q)$ distribution, and so with non-zero variance, $\sigma_{q,id}$, given by eq. S10. The true variance in gland frequency was therefore calculated as a difference between the experimental variance obtained from fit, $\sigma_{q,fit}$, and that the ideal variance, $\sigma_{q,id}$, expected for a window of finite width

$$\Delta\sigma_q = \sigma_q^{fit} - \sigma_q^{id} \tag{S13}$$

As a final step we expressed the value of so obtained true variance in gland frequency with relation to an ideal variance expected for undisturbed gland structure

$$\sigma_q = \frac{\Delta\sigma_q}{\sigma_q^{id}} \tag{S14}$$

Collecting σ_q values for all window positions (x_w, y_w), results in an image of gland frequency variance, $\sigma_q(x_w, y_w)$.

Image of gland orientation, θ_0

An angle of a gland structure, θ_0, was obtain by fitting the experimental angular density function $p(\theta)$ (eq.S9) to von Mises probability density function [2] (an analogue of the normal distribution in directional statistics) given by:

$$p(\theta) = \frac{\exp\left(\kappa\cos(\theta - \theta_0)\right)}{2\pi \mathrm{I}_o(\kappa)} \tag{S15}$$

where $I_0(\kappa)$ stands for modified Bessel function of order 0, κ^{-1} is an analogues of to σ^2(variance) in normal distribution, and θ_0 is a measure of the location of the maximum of the distribution (an analogues of mean in normal distribution). Collecting θ_0 values for all window position results in an image of gland orientation, $\theta_0(x_w, y_w)$.

For regions with poor gland structure an estimated gland orientation happens to be inaccurate. For this reason we smoothen an gland orientation image using a Gaussian kernel of size 3x3. This approach allows to estimate the gland orientation by considering the orientation of its immediate neighborhood.

Image of gland orientation variance, σ_θ

The value of gland orientation variance, $\sigma_{\theta,fit}$, can be found from parameter κ obtained by fitting the experimental angular density function $p(\theta)$ to eq.S15.

$$\sigma_{\theta,fit} = 1/\sqrt{\kappa} \tag{S16}$$

However, the fact that the Gaussian window used in 2D STFT analysis has finite width is reflected in finite width of the PSD (and so in $p(\theta)$ distribution). As a result, even ideal uni-directional periodic feature (for which no variance in $p(\theta)$ distribution should be expected) will be described by finite $p(\theta)$ distribution, and so with non-zero variance, $\sigma_{\theta,id}$, given by eq.S11.

The true variance in gland orientation was therefore calculated as a difference between the experimental variance obtained from fit, $\sigma_{\theta,fit}$, and that the ideal variance, $\sigma_{\theta,id}$, expected for a window of chosen width

$$\Delta\sigma_\theta = \sigma_{\theta,fit} - \sigma_{\theta,id} \tag{S17}$$

As a final step we expressed so obtained true variance in gland orientation relative to the ideal variance expected for undisturbed gland structure

$$\sigma_\theta = \frac{\Delta\sigma_\theta}{\sigma_{\theta,id}} \tag{S18}$$

Collecting σ_θ values for all window positions (x_w, y_w), results in an image of gland orientation variance, $\sigma_\theta(x_w, y_w)$.

Image of frequency gradient, G_q

The frequency gradient image was created to observe the rate of local changes in the meibomian gland frequencies. It was calculated from the image of gland frequency $q_0(x_w, y_w)$ using Sobel filter [1]. The edges of calculated frequency gradient image were eroded with Regional mask in order to clear out edge artifacts.

Image of angular incoherence, C_θ

The angular incoherence image was created to present local fluctuations in gland orientation. From an image of gland orientations, θ_0, small regions (composed of BxB blocks) of angular values were extracted. The size of that region was set to 3x3 (B=3). The value of angular incoherence, C_θ, for a central block $\theta_0(x_0, y_0)$ was defined as

$$C_\theta = \frac{\sum_{(i,j)\in B} \left|\sin\left(\theta_0(x_0,y_0) - \theta_0(x_i,y_i)\right)\right|}{B\times B} \tag{S19}$$

where $\theta_0(x_i, y_i)$ are the orientation values in neighboring blocks. As follows from eq.S19 if the angle of the central block is similar to an angle of its neighbor then their difference is close to 0, on so is the sine of the difference. As a result, the angular incoherence value is low when the orientation in central block is similar to orientation in the surrounded blocks.

An image of angular incoherence, $C_\theta(x_w, y_w)$ was obtained by calculating C_θ values for each pixel in $\theta_\theta(x_w, y_w)$ image.

Fig.S4 shows how C_θ was calculated for two arbitrary positions of the block region.

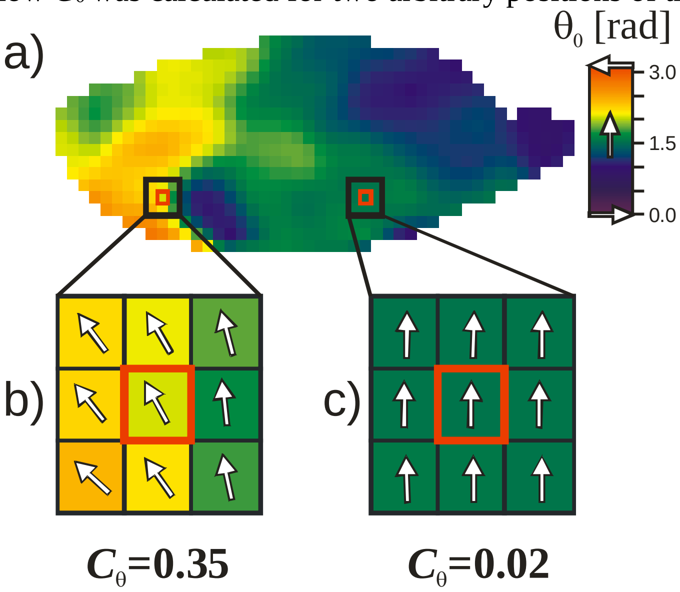

Fig.S4 Method for calculation angular incoherence, C_θ. a) image of gland orientations, θ_0 from which 3x3 blocks of pixels are extracted. b) Blocks of high angular incoherence. Note that in these blocks arrows indicating gland direction show range of orientations. c) Blocks of low angular incoherence. In these region of image gland orientation does not change much. The value of angular incoherence is calculated using eq.S19 for a central block (indicated by red squares). The image of angular incoherence is produced by calculating C_θ for every pixel in image of gland orientations.

Region mask

The region mask allows to distinguish the area of the Meibomian glands from the image background. It was used for the purpose of erosion the edges of intrinsic images to avoid edge artifacts.

In order to obtain region mask, an energy located within windowed image was obtained as an integral of its PSD

$$e = \log\left\{ \int_q \int_\theta |F(q,\theta)|^2 \right\} \tag{S20}$$

Collecting e values for all window positions (x_w, y_w), results in an image of energy, e (x_w, y_w). The region mask was then obtained by thresholding the energy image (Fig.S5).

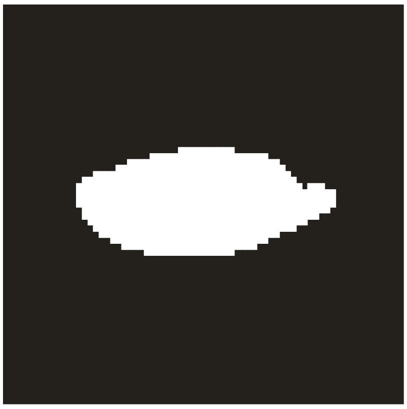

Fig.S5 The region mask obtained by thresholding the energy image obtained from eq.S20.

[1] Jähne, B., Scharr, H., & Körkel, S. (1999). Principles of filter design. Handbook of Computer Vision and Applications.

[2] Forbes, C., Evans, M., Hastings, N., & Peacock, B. (2011). Statistical distributions. John Wiley & Sons.
