# Supplementary material for: 2D Short-Time Fourier Transform for local morphological analysis of meibomian gland images
Source: PLoS One. 2022 Jun 24;17(6):e0270473. doi: 10.1371/journal.pone.0270473 (PMC9491703; doi:10.1371/journal.pone.0270473)
Supplement: S5 Appendix — (PDF) [file pone.0270473.s005.pdf]

S4. Quantification of intrinsic images

Each inherent image was quantified by 5 objective measures (features), being the Shannon entropy (E) and the first 4 moments of distribution of its values (namely: mean (M), variance (Var), skewness (Skw) and kurtosis (Krt)).
Shannon entropy for a distribution (histogram) of a random variable X (that is inherent image quantity) was calculated taking into consideration constant width of histogram bin w

$$E(X) = -\sum_{k=1}^{n} p_k \log p_k + \log w \tag{S21}$$

where p_k are bins probabilities (histogram values) [1].

This approach quantify each of six inherent images with 5 objective measures, giving total of 30 descriptive features for each Meibomian gland image.

As a naming convention, for each feature we use a symbol of distribution measure followed by an inherent quantity given in parenthesis. For example, Var(C_θ) means variance of angular incoherence distribution, whereas E(q_0) means entropy of gland frequency distribution.

[1] Rich R., Tracy J. The relationships among expected inflation, disagreement, and uncertainty: evidence from matched point and density forecasts. Rev Econ Stat 2010, *92*(1), 200-207. https://doi.org/10.1162/rest.2009.11167.
