# Supplementary material for: 2D Short-Time Fourier Transform for local morphological analysis of meibomian gland images
Source: PLoS One. 2022 Jun 24;17(6):e0270473. doi: 10.1371/journal.pone.0270473 (PMC9491703; doi:10.1371/journal.pone.0270473)
Supplement: S6 Appendix — (PDF) [file pone.0270473.s006.pdf]

S5. Dimensionality reduction and image categorization

Using our approach each Meibomian image can be described by 30 independent features. Finding regularities in such a high-dimension data can be difficult and requires proper data analysis procedure. To get more important information out of the whole set of data we firstly used Principal Component Analysis (PCA) [1,2].This method consists of projecting each 30-dimentional data point onto new axes (principal components) while preserving as much of the data's variation as possible. Because in most cases only the first few principal components contain most of the variance in data, the method is commonly used for dimensionality reduction.

In Fig.S6 the result of PCA analysis is shown. Scree plot in panel a) shows the variances explained by first 10 principal components. It is clear that focusing only on the first two PCA components is enough to explain nearly 50% of all variation in the whole set of data. The correlation plot between the first two PCA components is presented on panel b) for images subjectively classified as healthy, intermediate and unhealthy. Here it is worth to note that PCA belongs to the unsupervised class of machine learning algorithms meaning that the analysis does not require prior categorization of data set. The fact that the data points for each category seems separated in $PCA_1(PCA_2)$ representation suggest that this kind of analysis can be used as an objective classification routine. In panel c) the PCA loading (score) vectors, for each of 30 objective features, are presented in $PCA_1(PCA_2)$ representation. Projection of a loading vector for a given feature on a particular PCA axis indicates significance of this feature on this principal component (Fig.S6c).

All 30 features sorted according to their PCA1 loadings are given in Table S1. As follows from the data collected in tab.S1, an entropies and the means of distributions of intrinsic properties sensitive to variability in gland frequency and orientation (σ_q, σ_θ, C_θ, G_q) are the features with the most descriptive potential.

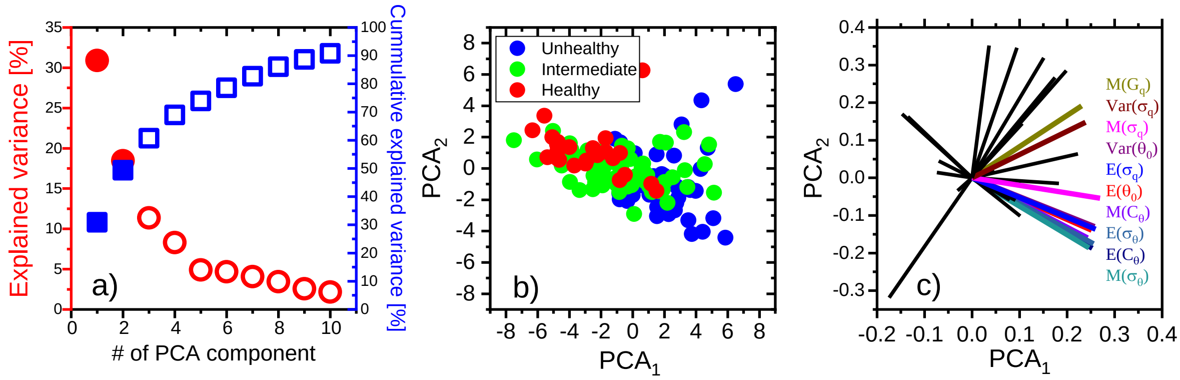

Fig.S6 a) Scree plot indicating how much variance in whole set of 30-dimentional data is explained by each of ten first ten PCA components (red dots). Blue squares shows the cumulative variance. Notice that first two PCA components are responsible for almost 50% of variance in whole data set. b) Correlation plot between two first PCA components. Notice that although PCA is unsupervised method (it does not make use of the a-priori knowledge about the existence of categories) it allows to separate the categories along PCA components. c) PCA score (loading) plot. Each of the 30 feature has corresponding vector (solid line) whose projection on particular PCA axis indicates its influence on this principal component value. 10 features of the highest projection on PCA1 axis are indicated with color lines and corresponding symbols.

Tab. S1 All 30 features sorted according to the highest weight they have on PCA1 component. Feature symbol includes name of distribution measure followed by an inherent quantity given in parenthesis. Two features indicated in red ($Var(\theta_0)$ and $M(q_0)$) correspond to global features (periodicity anisotropy parameter (κ^{-1}) and mean gland frequency ($\bar{q}$), respectively) derived in our earlier work [3].

Feature	PCA1 Loading
$M(\sigma_q)$	0.270
$E(\sigma_q)$	0.260
$Var(\theta_0)$	0.258
$E(\sigma_\theta)$	0.256
$E(C_\theta)$	0.253
$E(\theta_0)$	0.252
$M(\sigma_\theta)$	0.246
$M(C_\theta)$	0.243
$Var(\sigma_q)$	0.239
$M(G_q)$	0.230
$E(G_q)$	0.223
$Var(C_\theta)$	0.211
$Var(q_0)$	0.198
$E(q_0)$	0.183
$Skw(q_0)$	0.175
$Var(G_q)$	0.174
$Krt(q_0)$	0.151
$Skw(C_\theta)$	0.147
$Skw(G_q)$	0.143
$Krt(C_\theta)$	0.135
$Krt(G_q)$	0.106
$Var(\sigma_\theta)$	0.101
$Krt(\sigma_q)$	0.094
$Krt(\sigma_\theta)$	0.091
$Skw(\sigma_\theta)$	0.080
$M(q_0)$	0.074
$Krt(\theta_0)$	0.070
$Skw(\theta_0)$	0.047
$Skw(\sigma_q)$	0.036
$M(\theta_0)$	0.031

Differences in the values of the descriptive features found for each Meibomian image can be used to automatically categorize the images into subjective categories. There are many machine learning algorithms which can be utilized for this task: k-means clustering (unsupervised), support vector machine, neural networks, to mention just the most popular ones [3,4]. Here we test the performance of two widely used approaches based on PCA analysis and Linear Discriminant Analysis (LDA). Both methods are similar in a sense that they reduce dimensionality of data by projecting them on new axes. The difference is that PCA is an unsupervised method and it does not make use of the a-priori knowledge about the existence of categories. LDA on the other hand belongs to supervised class of learning algorithms and uses information about classes.

On Fig.S7 we show the result of PCA (panel a) and LDA (panel b) analysis performed on the same 30-dimentional set of data collected for all Meibomian images. After analysis the dimensionality of data can be reduced. We present our data as a correlation plot of the first two

components ($PCA_{1,2}$ and $LDA_{1,2}$). It is clear that both methods allow to separate the categories along their components plane. A simple comparison of the results presented in the Fig.S6 shows that the LDA analysis allows for a better separation of data for three subjective categories which is not surprising considering that LDA is supervised method.

To use the data dispersion observed in main panels of Fig.S7 for automatic image categorization, the marginal distributions of each of the component ($PCA_{1,2}$ and $LDA_{1,2}$) for each subjective category was first calculated. These distributions are shown as histograms on Fig.S7. It can be observed that for each type of analysis used, the maxima of the distribution appears in different location and shows some overlap. Next, the experimental histograms were described by normal distribution (Gaussian) to get continuous probability distribution of each component for each category (solid lines in marginals of Fig.S7). Knowing the probability distributions of a given component for each category, a simple threshold classifiers were created.

We tested the classifier based on the probability that an image is parametrized by certain value of first component (e.g. PCA_1) AND certain value of the second one (e.g. PCA_2). More specifically, an image being parametrized with a pair of component values (PCA_1/PCA_2 or LDA_1/LDA_2) is assigned to a category specified by the highest value of the product of corresponding probability functions ($p(PCA_1)p(PCA_2)$ or $p(LDA_1)p(LDA_2)$). The classification performance of this approach is presented in Table 1 of the main manuscript. The symbols of these classifiers are: $p(PCA_1)p(PCA_2)$ and $p(LDA_1)p(LDA_2)$.

Notice that $p(LDA_1)$ distributions for images classified as healthy and unhealthy barely overlap. This means that, based solely on $p(LDA_1)$, meaning that images belonging to these two categories can be distinguish with nearly 100% accuracy. Notice also that $p(LDA_2)$ for intermediate category is clearly moved away from probability distributions for other two categories (being close together). This information can be used to increase the effectiveness of automatic classification of images belonging to intermediate category.

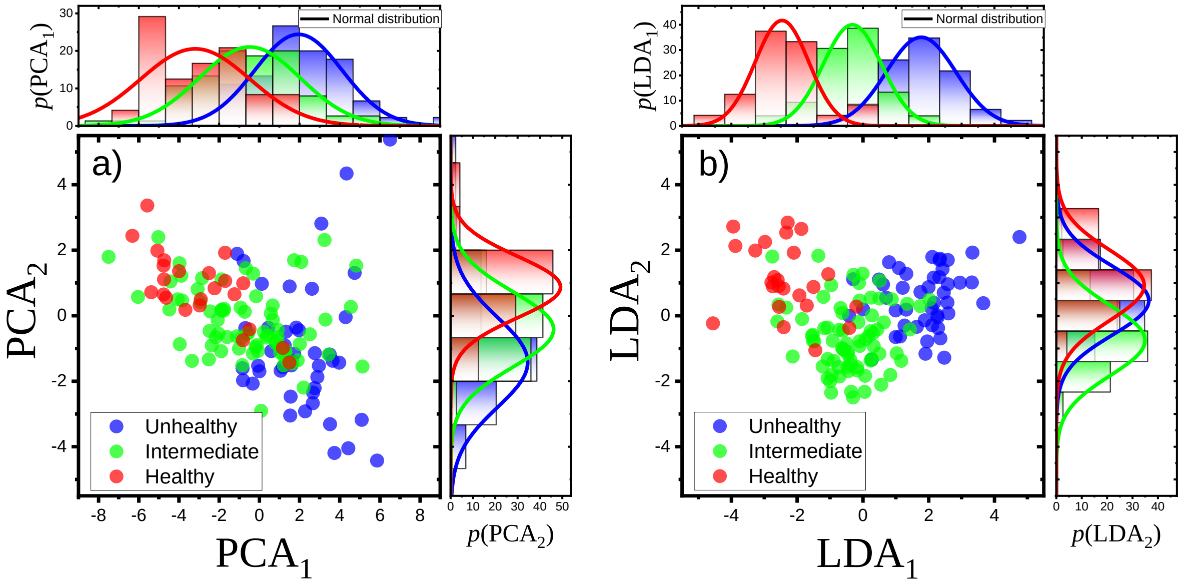

Fig.S7 Correlation plots between first two components of a) Principal Component Analysis (PCA) and b) Linear Discriminant Analysis (LDA) for images classified as healthy, intermediate and unhealthy. Marginal plots shows probability distributions of corresponding components. Notice that although both analysis provide noticeable separation of categories, the LDA analysis (being supervised method) provides much better clustering of classes.

[1] Jolliffe IT. Principal Component Analysis. 2nd ed. New York: Springer-Verlag; 2002. https://doi.org/10.1007/b98835.

[2] Martínez A. M., Kak A. C. PCA versus LDA. *IEEE Trans*. Pattern Anal. Mach. Intell. 2001, 23(2), 228-233. https://doi.org/10.1109/34.908974.

[3] Ciężar K., Pochylski M. 2D fourier transform for global analysis and classification of meibomian gland images. Ocul Surf 2020, 18(4), 865-870. https://doi.org/10.1016/j.jtos.2020.09.005.

[4] Celik T, Lee HK, Petznick A. Bioimage informatics approach to automated meibomian gland analysis in infrared images of meibography. J Optom. 2013; 6: 194–204. https://doi.org/10.1016/j.optom.2013.09.001.
