# Supplementary material for: 2D Short-Time Fourier Transform for local morphological analysis of meibomian gland images
Source: PLoS One. 2022 Jun 24;17(6):e0270473. doi: 10.1371/journal.pone.0270473 (PMC9491703; doi:10.1371/journal.pone.0270473)
Supplement: S7 Appendix — (PDF) [file pone.0270473.s007.pdf]

S7. Estimation of confidence interval for classification efficiency

Confidence interval for classification efficiency was estimated from the knowledge of probability density for each category (healthy, intermediate and unhealthy) and for each descriptive parameter (PCA1, PCA2 or LDA1, LDA2). The appropriate probability distributions are presented on Fig.S7 as marginal plots.
Our classifier was based on product of probability functions ($p(PCA_1)p(PCA_2)$ or $p(LDA_1)p(LDA_2)$). In this case, the probability that a *i*-th sample image (randomly selected from the existing set of data) will be classified as Healthy can be written as

$$H_i = \frac{p1_{H,i}\, p2_{H,i}}{p1_{H,i}\, p2_{H,i} + p1_{\mathrm{I},i}\, p2_{\mathrm{I},i} + p1_{\mathrm{U},i}\, p2_{\mathrm{U},i}} \tag{S22a}$$

where $p1_{H,i}$ is the value of probability density (here the red curve on top marginal plot on Fig.S7a) that *i*-th image, ground-truth classified as "Healthy", will be described by a value of PCA1 (or LDA1). Similarly, $p2_{H,i}$ is the density of probability that healthy image will be described by a value of PCA2 (LDA2). $p1_{I,i}$, $p2_{I,\mathrm{i}}$, $p1_{U,i}$ and $p2_{U,\mathrm{i}}$ are corresponding probability values for Intermediate and Unhealthy images, respectively.

Similar equations can be written for I_i and U_i being probabilities that a *i*-th image will be classified as Intermediate and Unhealthy, respectively

$$I_i = \frac{p1_{I,i}\, p2_{I,i}}{p1_{H,i}\, p2_{H,i} + p1_{\mathrm{I},i}\, p2_{\mathrm{I},i} + p1_{\mathrm{U},i}\, p2_{\mathrm{U},i}} \tag{S22b}$$

$$H_i = \frac{p1_{U,i}\, p2_{U,i}}{p1_{H,i}\, p2_{H,i} + p1_{\mathrm{I},i}\, p2_{\mathrm{I},i} + p1_{\mathrm{U},i}\, p2_{\mathrm{U},i}} \tag{S22c}$$

Using equations S22 we have calculated the values of H_i, I_i and U_i for every image obtaining a sets of classification probability values. As a measure of classification uncertainty we have taken the standard deviation (SD) of these sets ($SD_H \cong 0.31$, $SD_I \cong 0.18$ and $SD_H \cong 0.31$). Assuming 95% confidence interval the margins of errors in classification (ΔH, ΔI and ΔU) were calculated from

$$\Delta H = 1.96\frac{SD_H}{\sqrt{N_H}}$$

$$\Delta I = 1.96\frac{SD_I}{\sqrt{N_I}} \tag{S23}$$

$$\Delta U = 1.96\frac{SD_U}{\sqrt{N_U}}$$

where N_H=24, N_I=75 and N_U=46 are number of images in each category.
The categorization efficiencies (*H*, *I* and *U*) collected in Table 1 of the main manuscript are presented in form $H \pm \Delta H$.
