# Supplementary figures and images for: 2D Short-Time Fourier Transform for local morphological analysis of meibomian gland images (part 2 of 2)
Source: PLoS One. 2022 Jun 24;17(6):e0270473. doi: 10.1371/journal.pone.0270473 (PMC9491703; doi:10.1371/journal.pone.0270473)

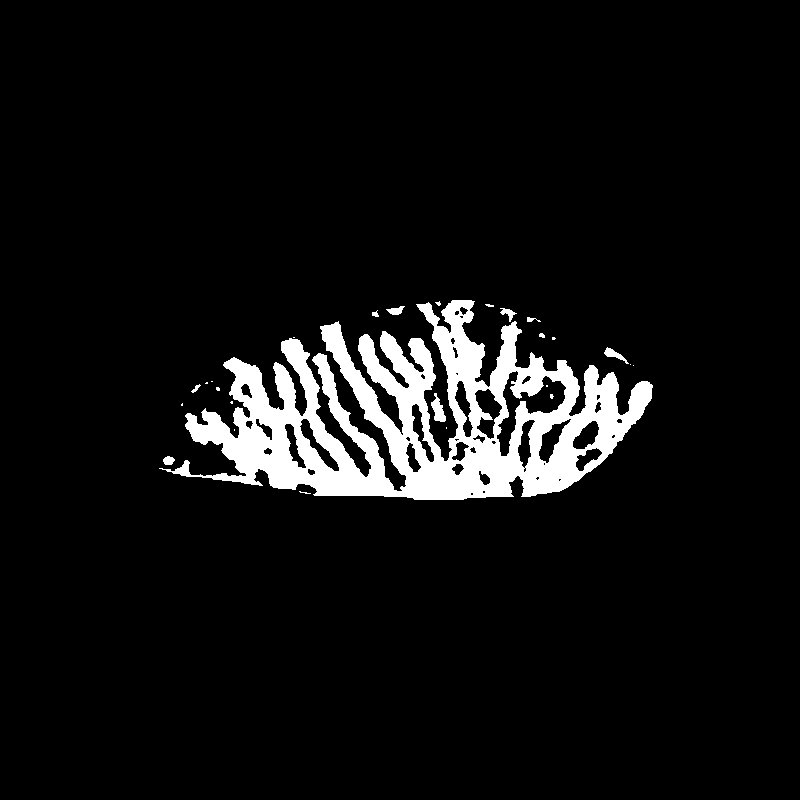

Supplement: S1 Raw images — (ZIP) [file pone.0270473.s008.zip › Unhealthy/unhealthy 11.jpg]

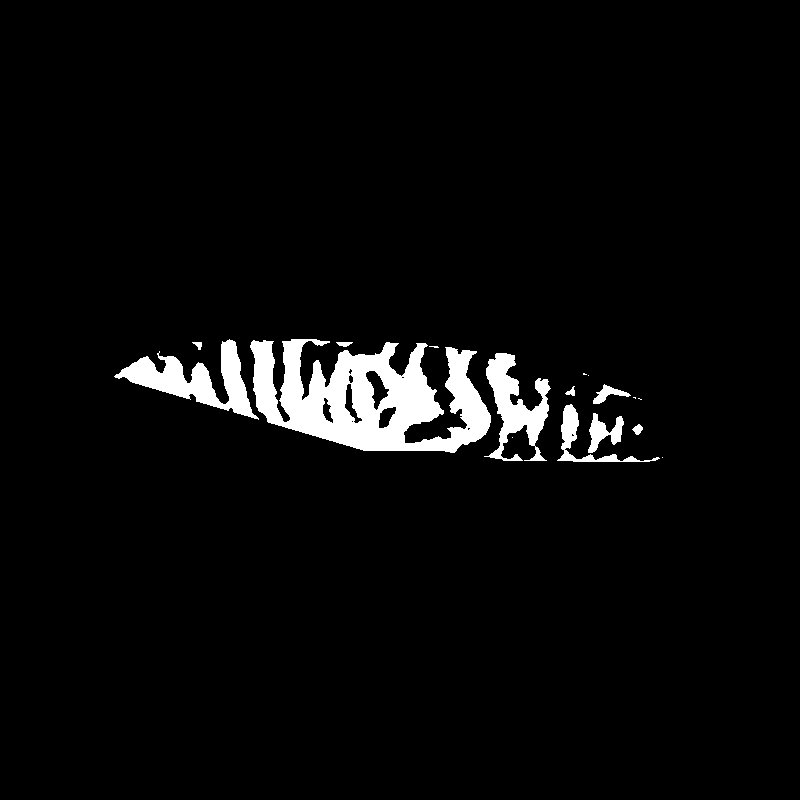

Supplement: S1 Raw images — (ZIP) [file pone.0270473.s008.zip › Unhealthy/unhealthy 12.jpg]

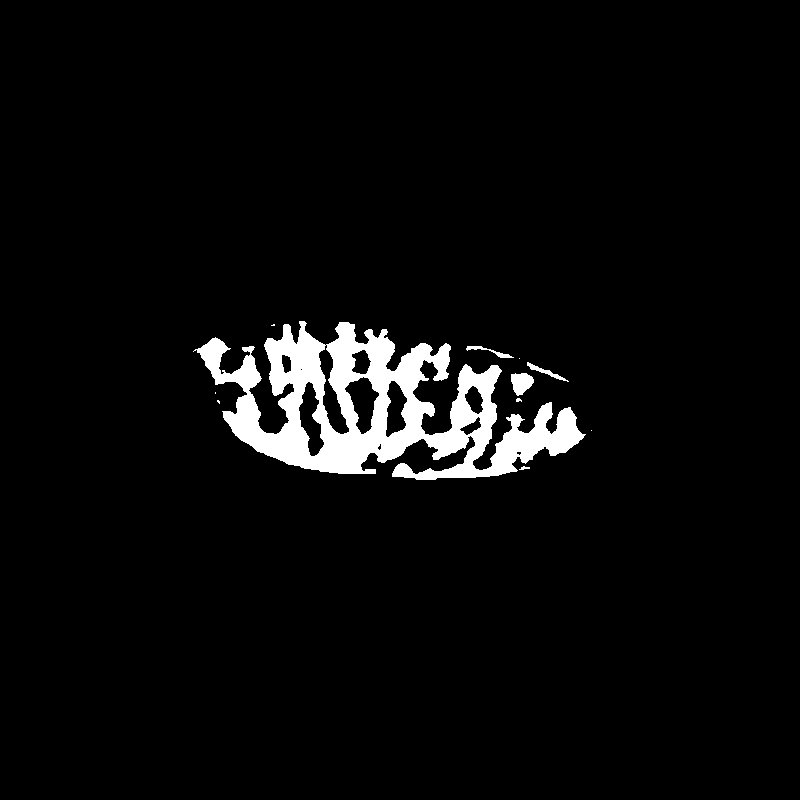

Supplement: S1 Raw images — (ZIP) [file pone.0270473.s008.zip › Unhealthy/unhealthy 13.jpg]

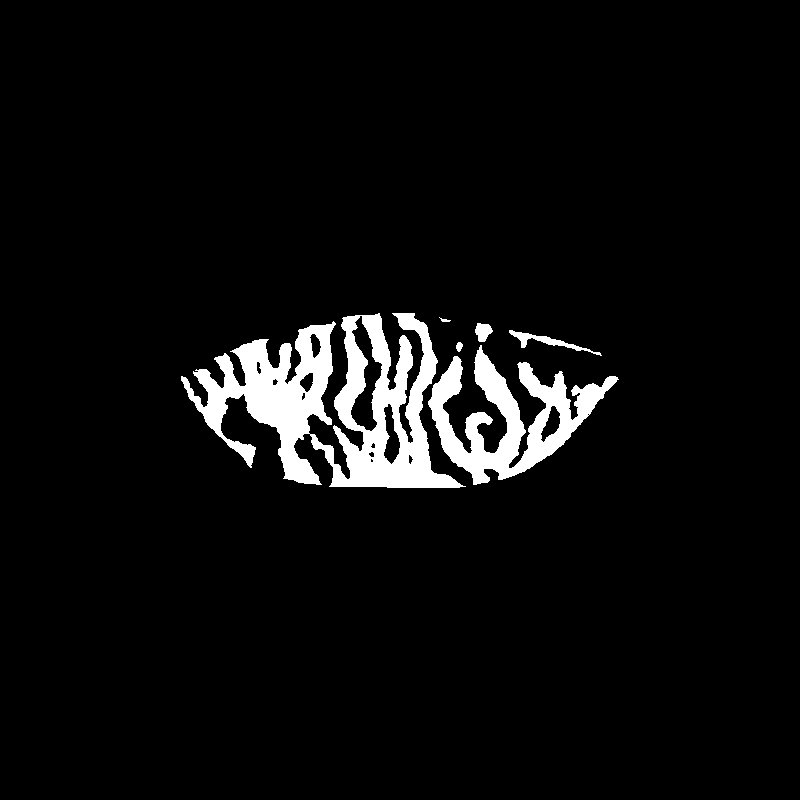

Supplement: S1 Raw images — (ZIP) [file pone.0270473.s008.zip › Unhealthy/unhealthy 14.jpg]

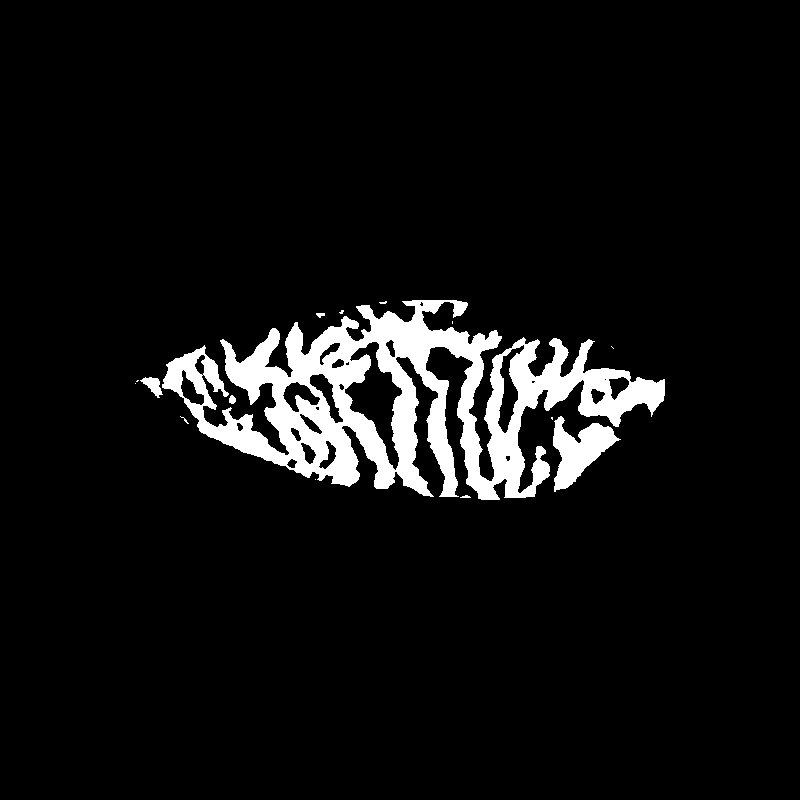

Supplement: S1 Raw images — (ZIP) [file pone.0270473.s008.zip › Unhealthy/unhealthy 15.jpg]

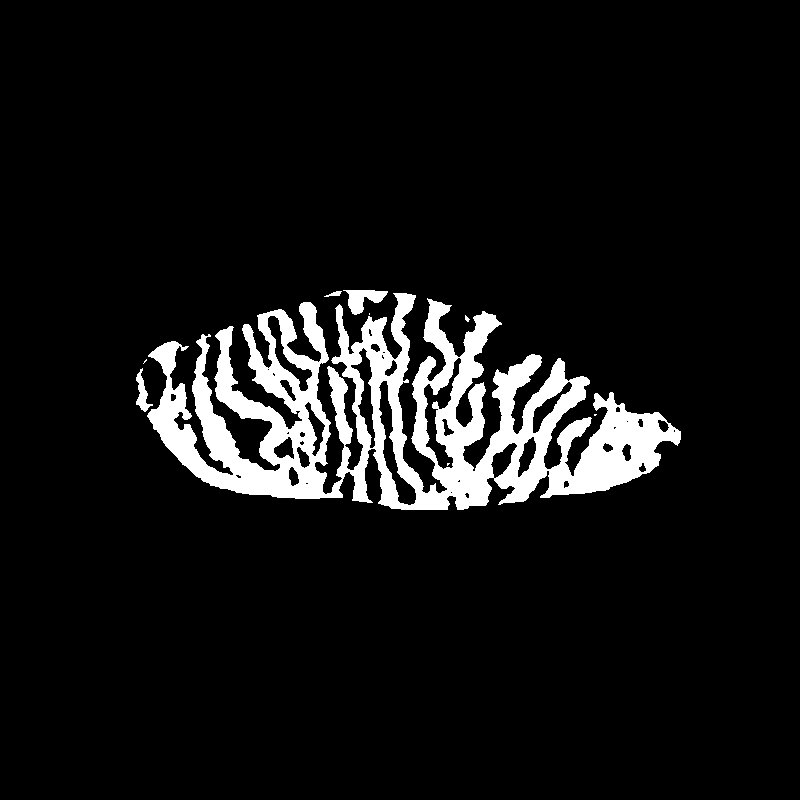

Supplement: S1 Raw images — (ZIP) [file pone.0270473.s008.zip › Unhealthy/unhealthy 16.jpg]

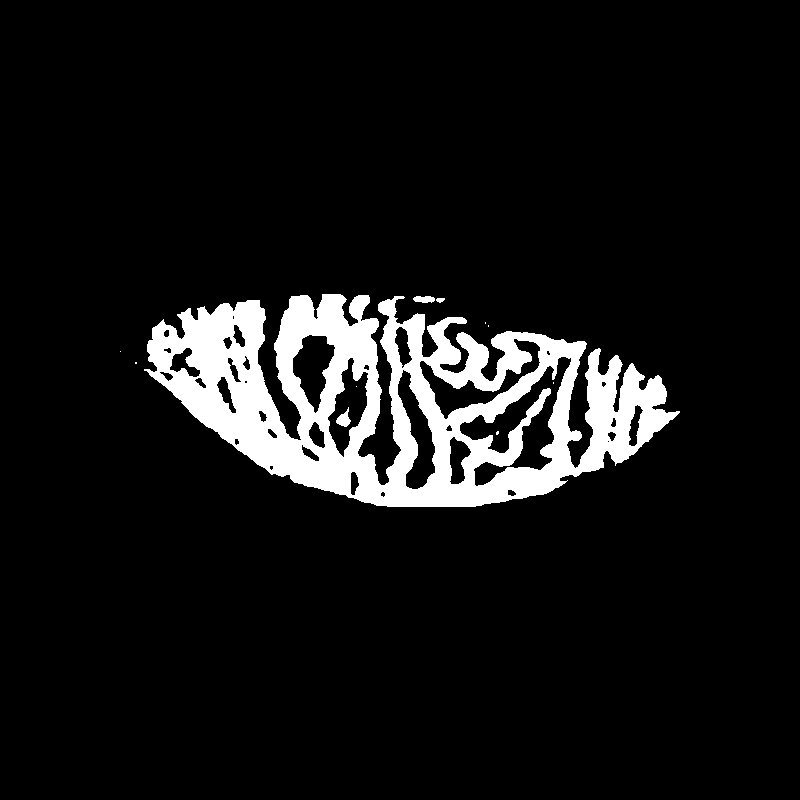

Supplement: S1 Raw images — (ZIP) [file pone.0270473.s008.zip › Unhealthy/unhealthy 17.jpg]

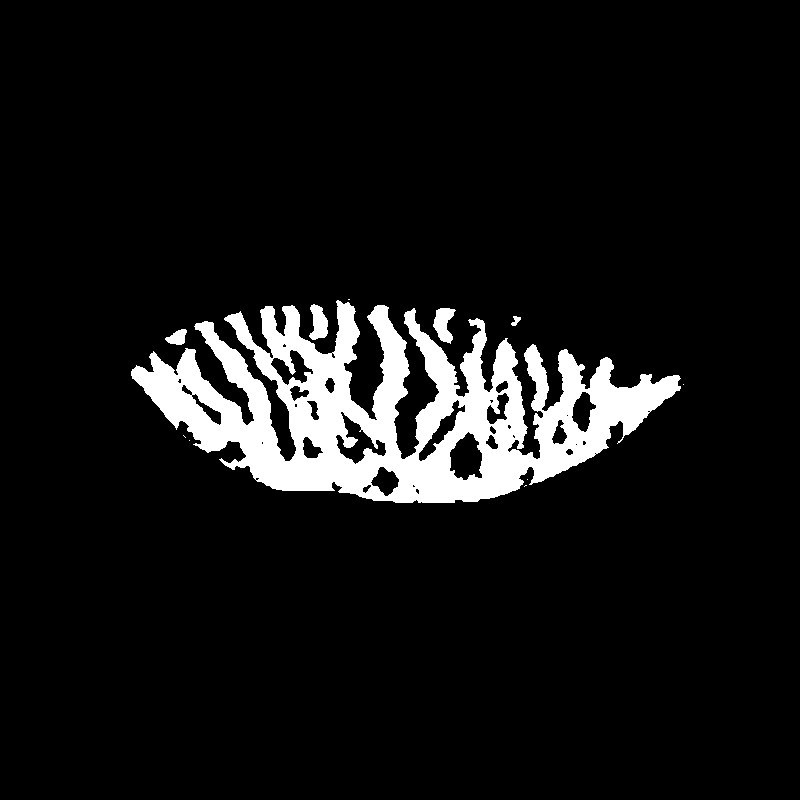

Supplement: S1 Raw images — (ZIP) [file pone.0270473.s008.zip › Unhealthy/unhealthy 18.jpg]

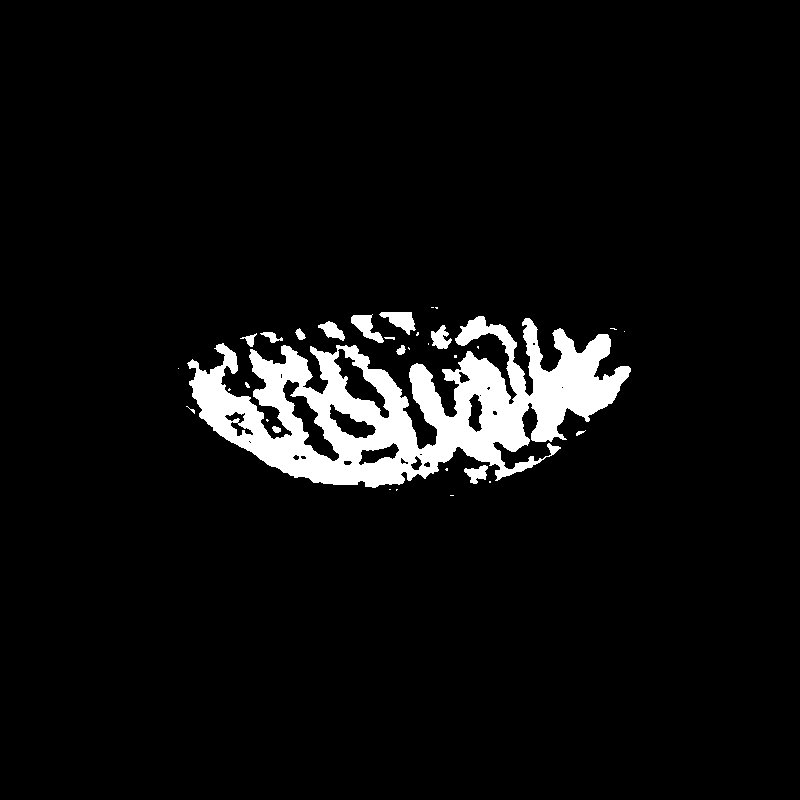

Supplement: S1 Raw images — (ZIP) [file pone.0270473.s008.zip › Unhealthy/unhealthy 19.jpg]

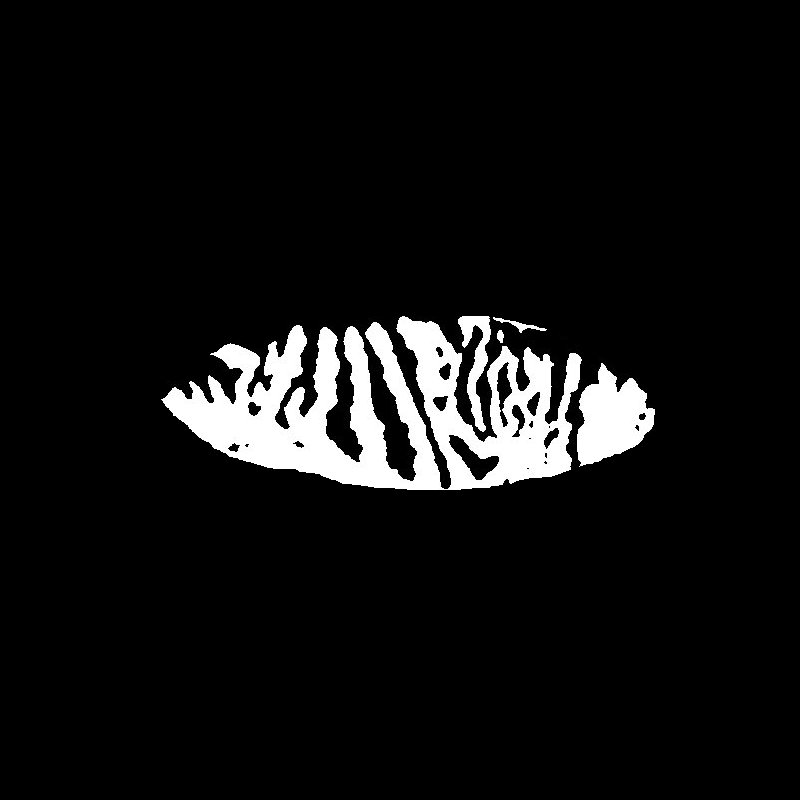

Supplement: S1 Raw images — (ZIP) [file pone.0270473.s008.zip › Unhealthy/unhealthy 2.jpg]

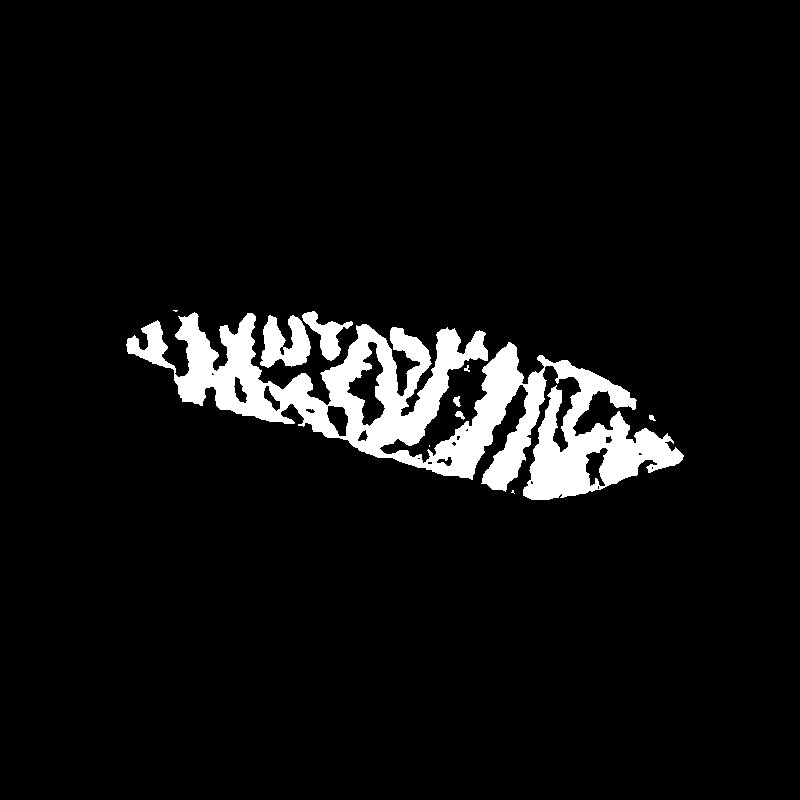

Supplement: S1 Raw images — (ZIP) [file pone.0270473.s008.zip › Unhealthy/unhealthy 20.jpg]

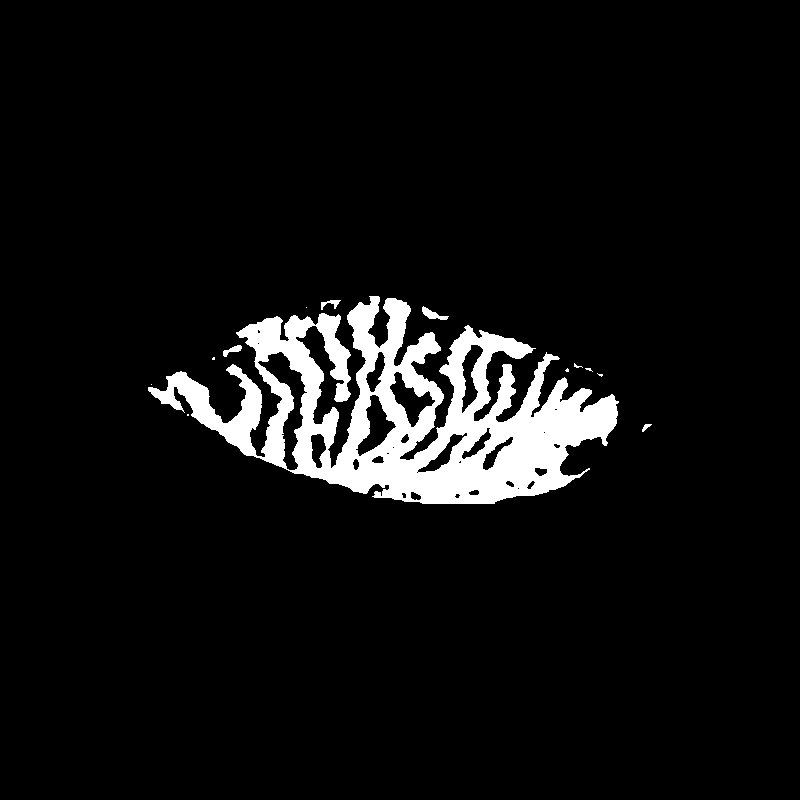

Supplement: S1 Raw images — (ZIP) [file pone.0270473.s008.zip › Unhealthy/unhealthy 21.jpg]

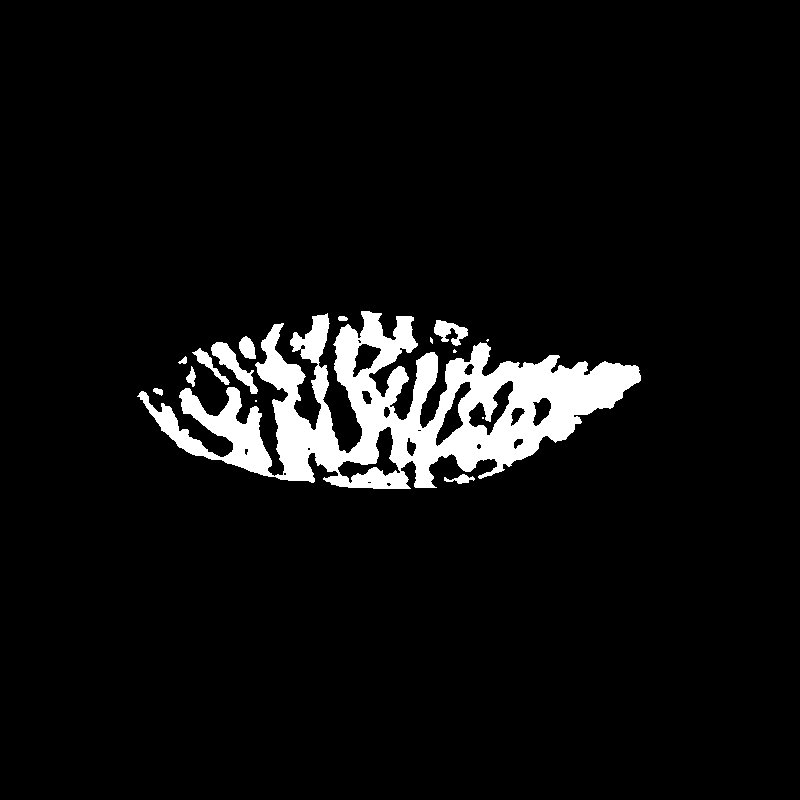

Supplement: S1 Raw images — (ZIP) [file pone.0270473.s008.zip › Unhealthy/unhealthy 22.jpg]

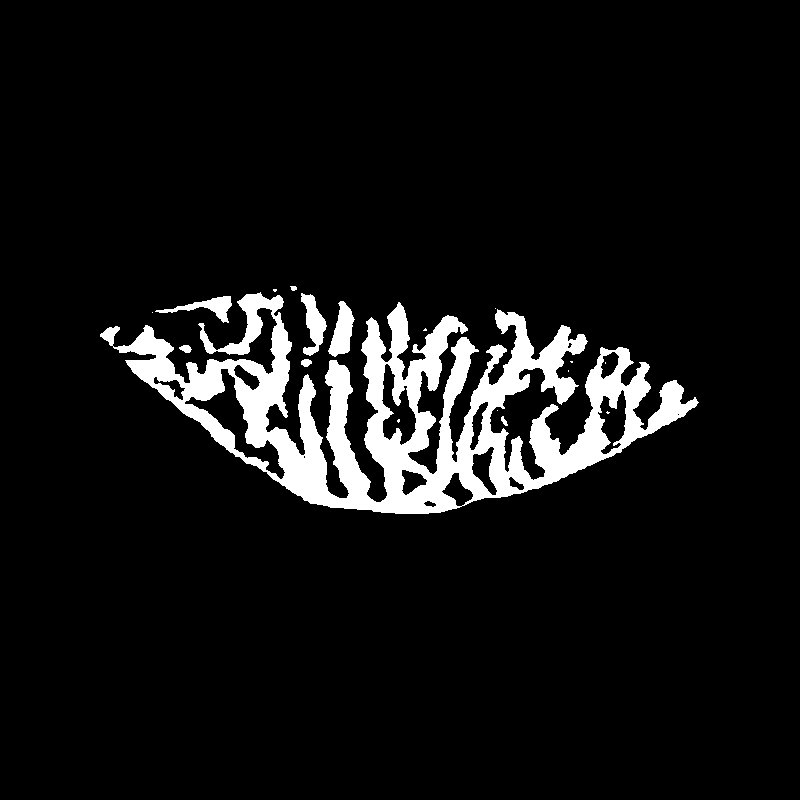

Supplement: S1 Raw images — (ZIP) [file pone.0270473.s008.zip › Unhealthy/unhealthy 23.jpg]

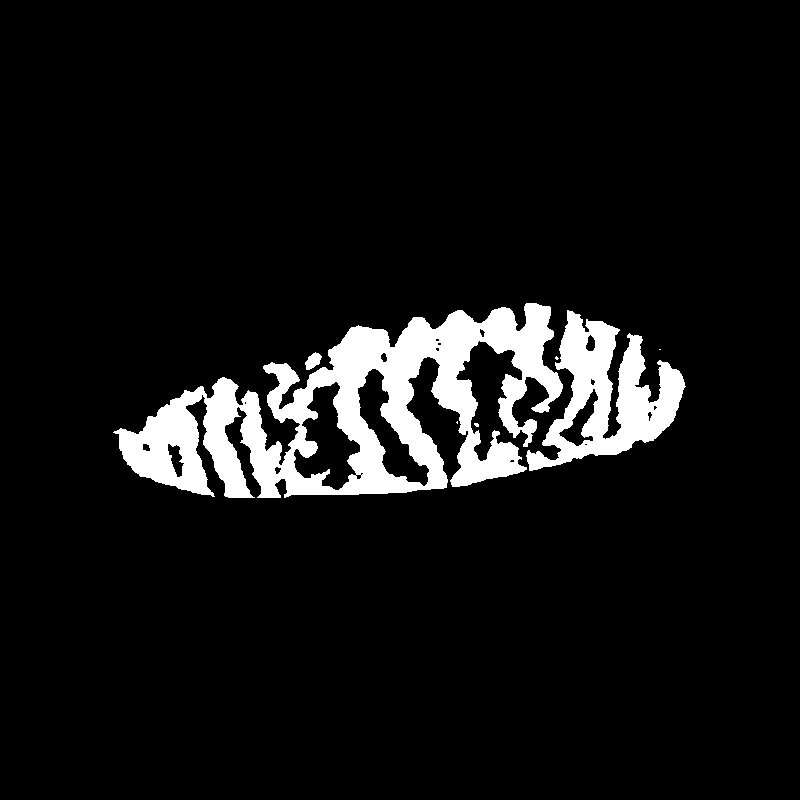

Supplement: S1 Raw images — (ZIP) [file pone.0270473.s008.zip › Unhealthy/unhealthy 24.jpg]

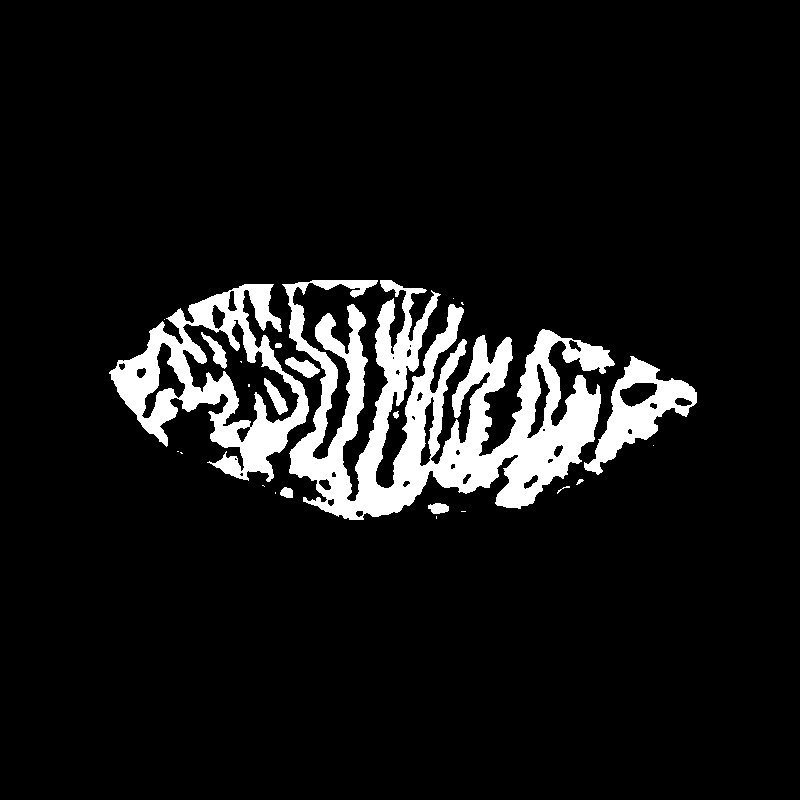

Supplement: S1 Raw images — (ZIP) [file pone.0270473.s008.zip › Unhealthy/unhealthy 25.jpg]

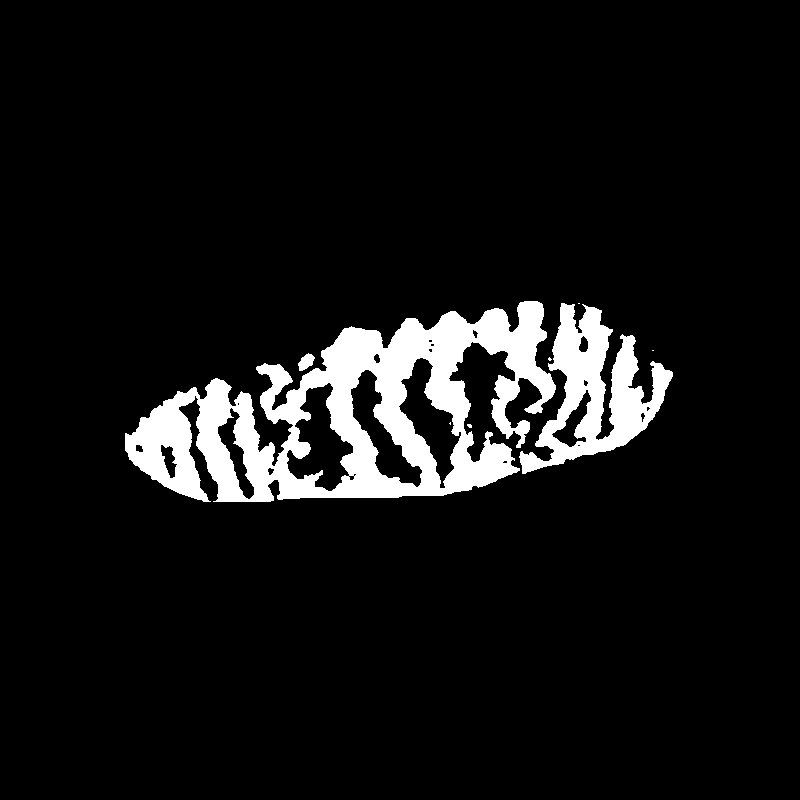

Supplement: S1 Raw images — (ZIP) [file pone.0270473.s008.zip › Unhealthy/unhealthy 26.jpg]

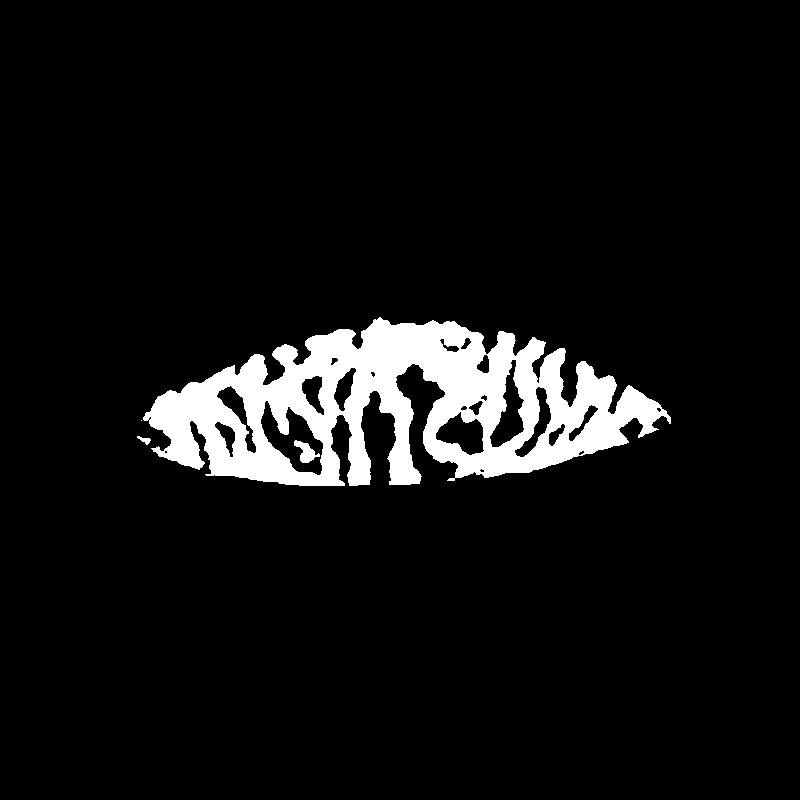

Supplement: S1 Raw images — (ZIP) [file pone.0270473.s008.zip › Unhealthy/unhealthy 27.jpg]

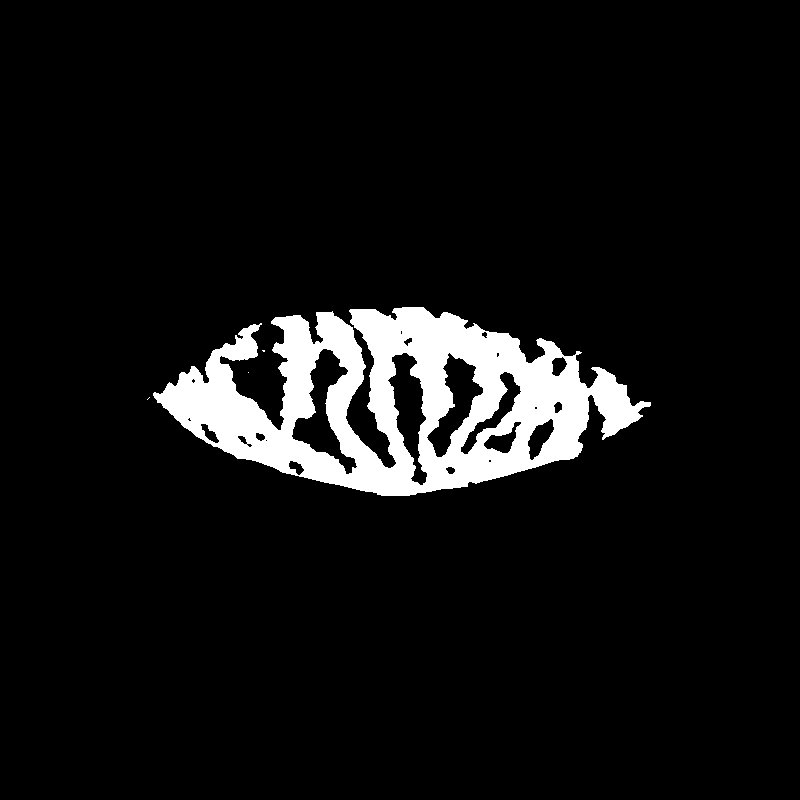

Supplement: S1 Raw images — (ZIP) [file pone.0270473.s008.zip › Unhealthy/unhealthy 28.jpg]

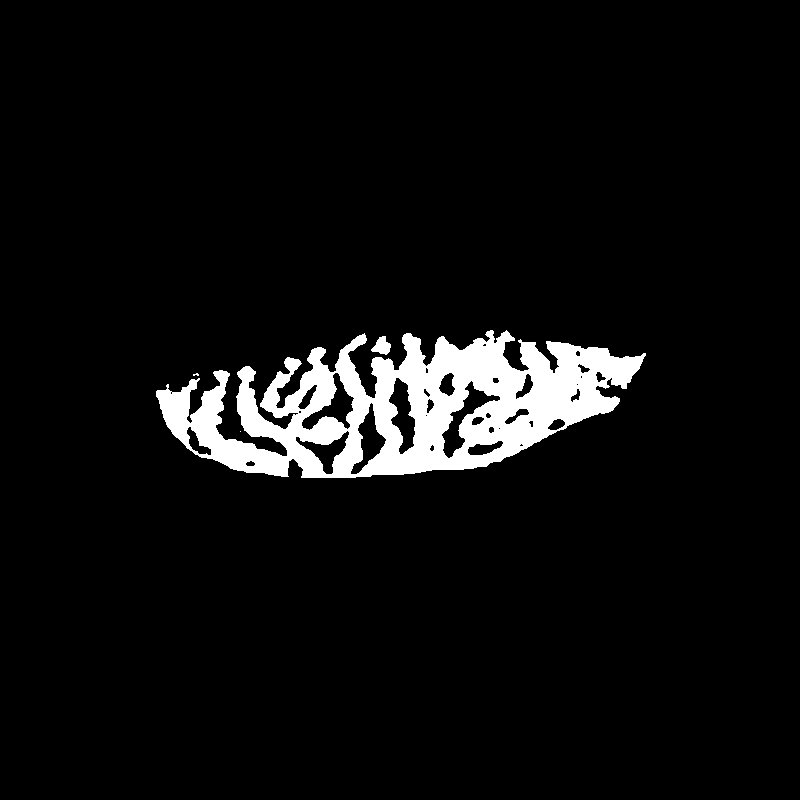

Supplement: S1 Raw images — (ZIP) [file pone.0270473.s008.zip › Unhealthy/unhealthy 29.jpg]

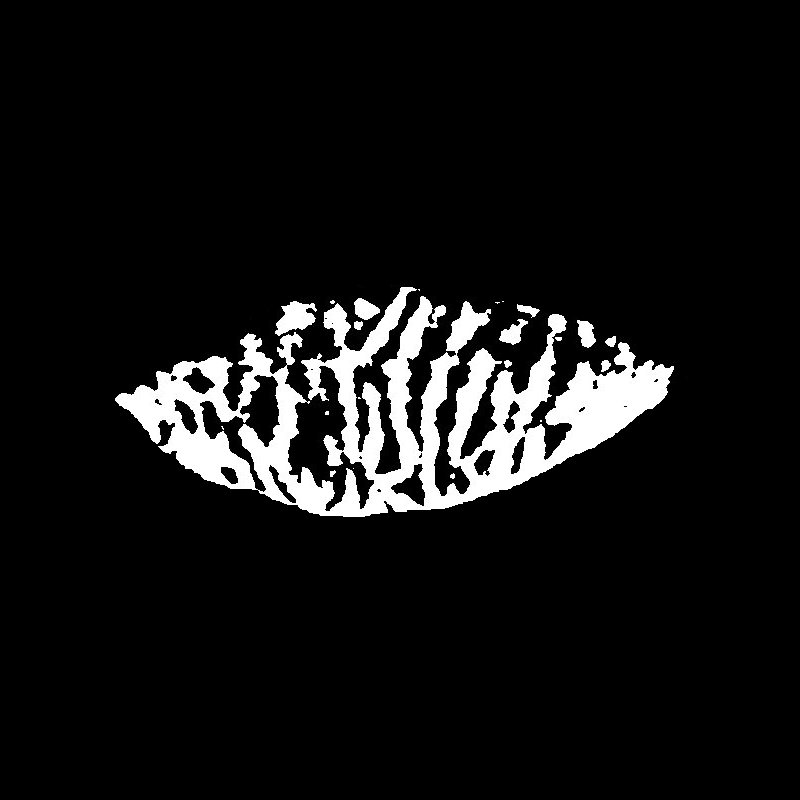

Supplement: S1 Raw images — (ZIP) [file pone.0270473.s008.zip › Unhealthy/unhealthy 3.jpg]

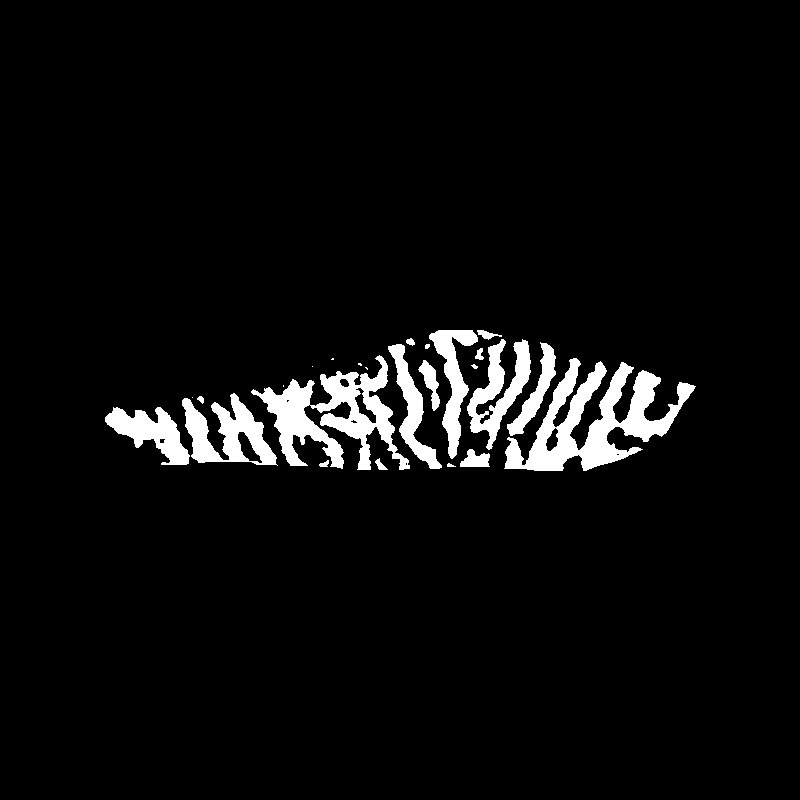

Supplement: S1 Raw images — (ZIP) [file pone.0270473.s008.zip › Unhealthy/unhealthy 30.jpg]

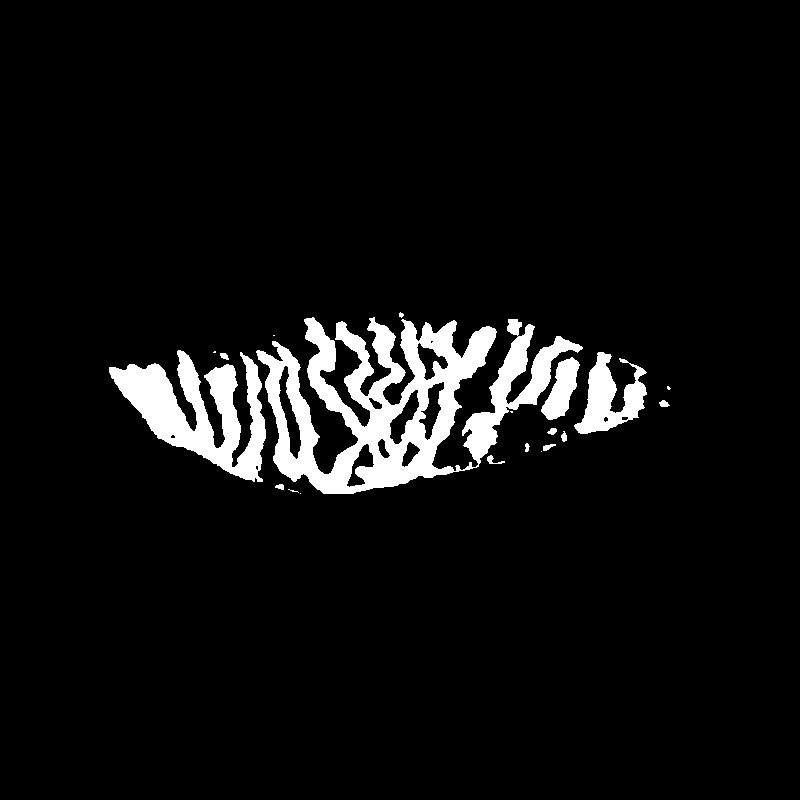

Supplement: S1 Raw images — (ZIP) [file pone.0270473.s008.zip › Unhealthy/unhealthy 31.jpg]

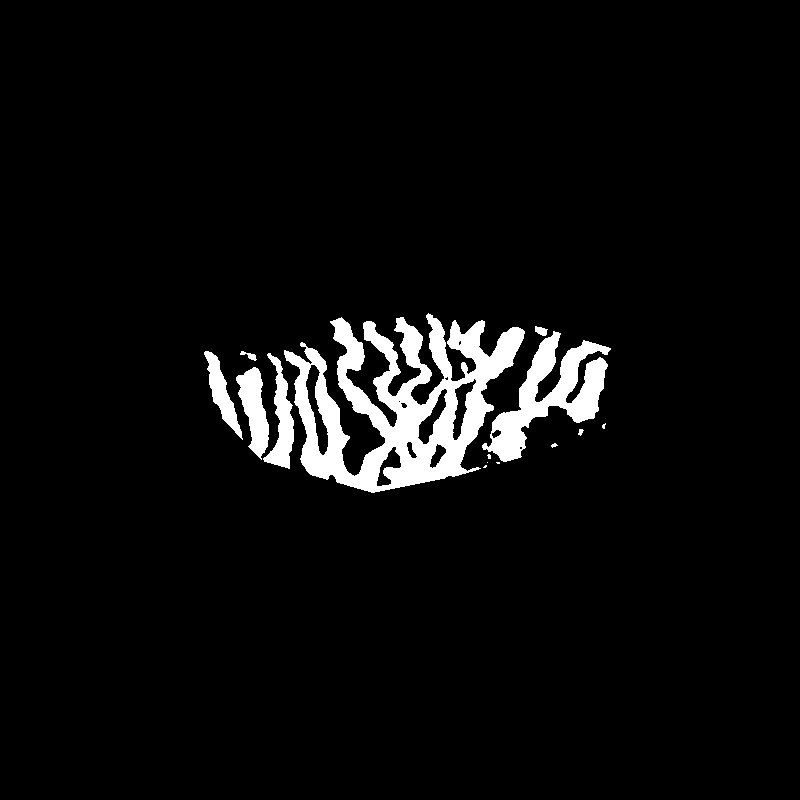

Supplement: S1 Raw images — (ZIP) [file pone.0270473.s008.zip › Unhealthy/unhealthy 32.jpg]

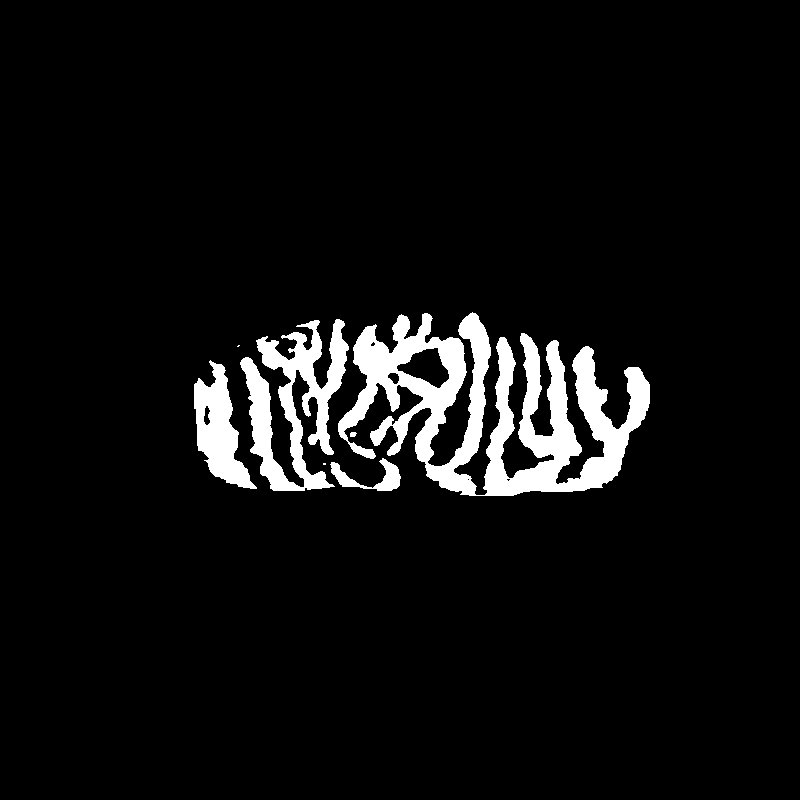

Supplement: S1 Raw images — (ZIP) [file pone.0270473.s008.zip › Unhealthy/unhealthy 33.jpg]

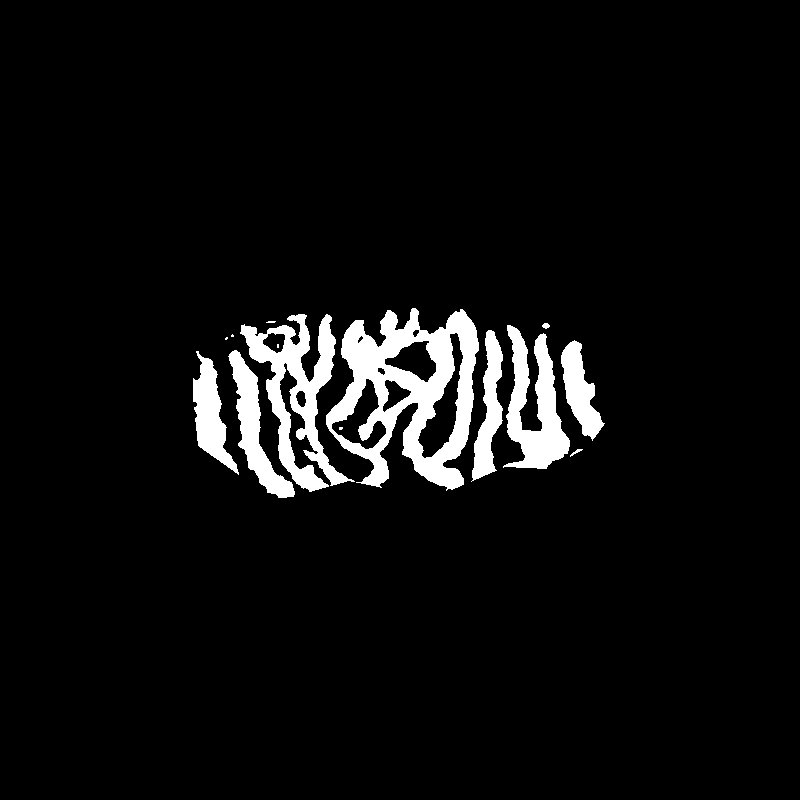

Supplement: S1 Raw images — (ZIP) [file pone.0270473.s008.zip › Unhealthy/unhealthy 34.jpg]

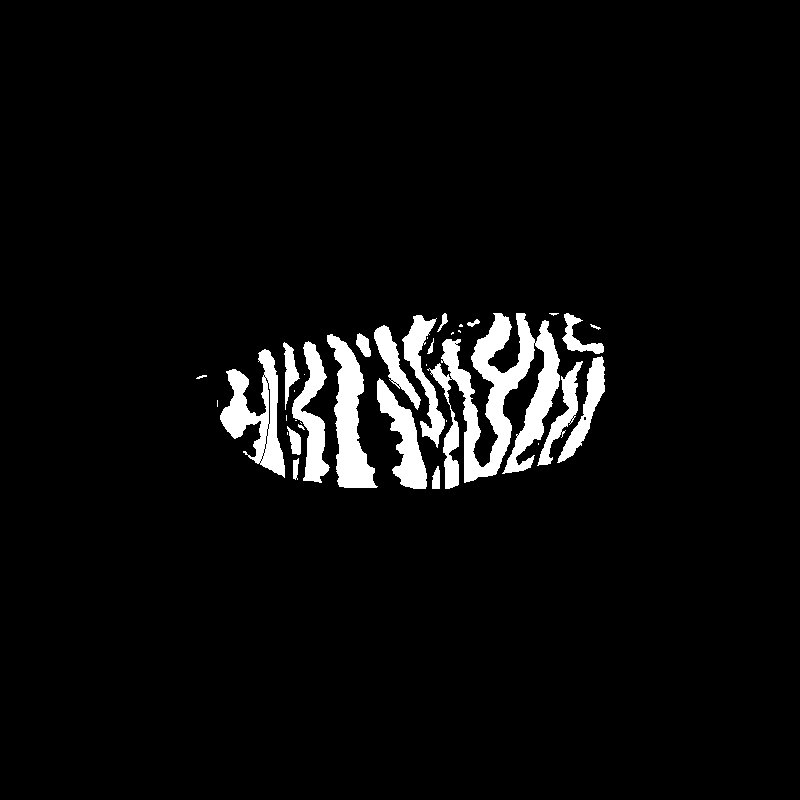

Supplement: S1 Raw images — (ZIP) [file pone.0270473.s008.zip › Unhealthy/unhealthy 35.jpg]

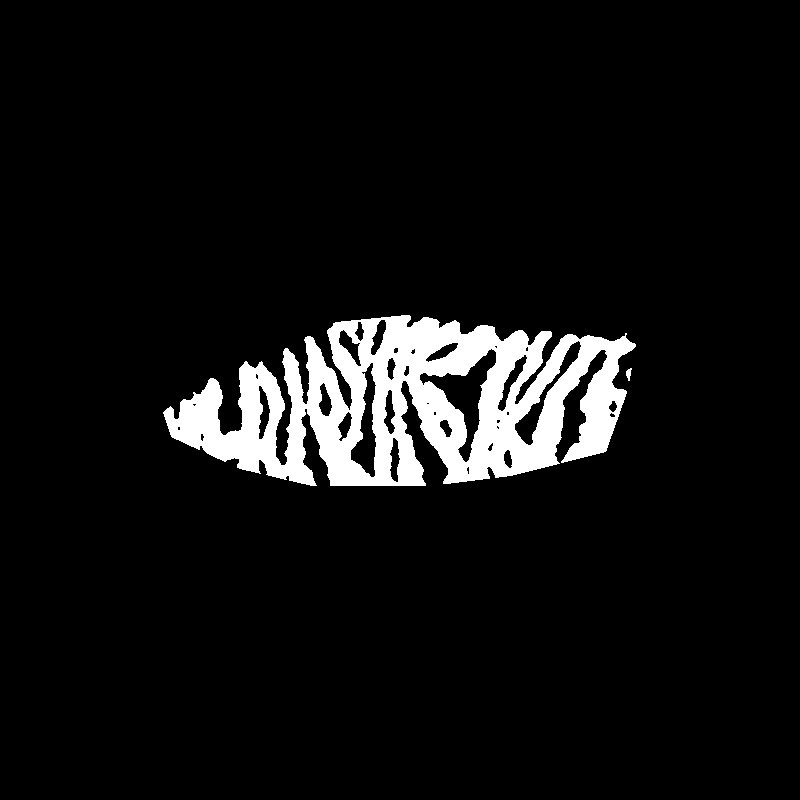

Supplement: S1 Raw images — (ZIP) [file pone.0270473.s008.zip › Unhealthy/unhealthy 36.jpg]

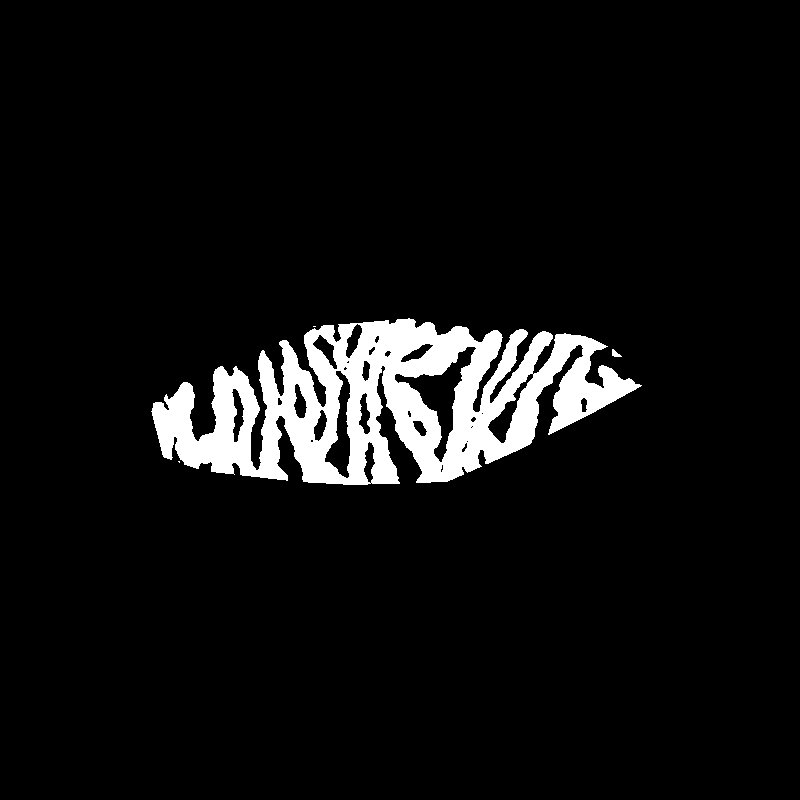

Supplement: S1 Raw images — (ZIP) [file pone.0270473.s008.zip › Unhealthy/unhealthy 37.jpg]

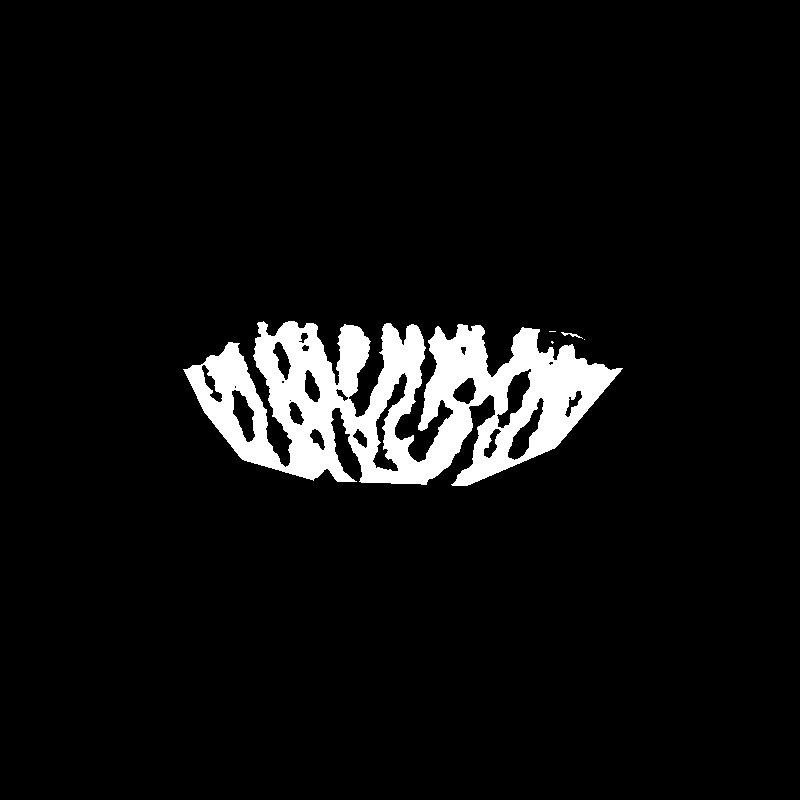

Supplement: S1 Raw images — (ZIP) [file pone.0270473.s008.zip › Unhealthy/unhealthy 38.jpg]

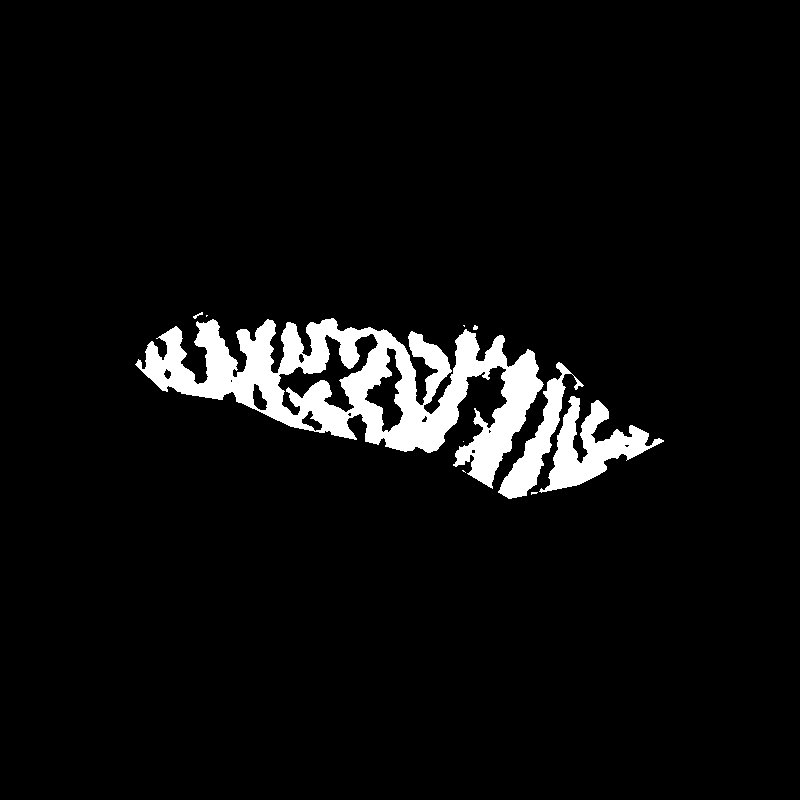

Supplement: S1 Raw images — (ZIP) [file pone.0270473.s008.zip › Unhealthy/unhealthy 39.jpg]

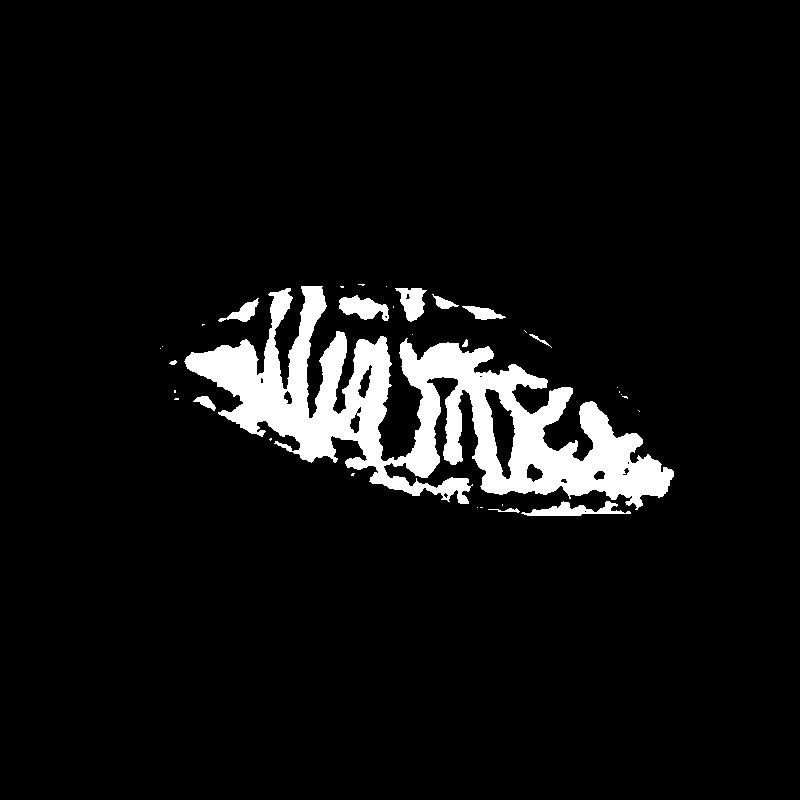

Supplement: S1 Raw images — (ZIP) [file pone.0270473.s008.zip › Unhealthy/unhealthy 4.jpg]

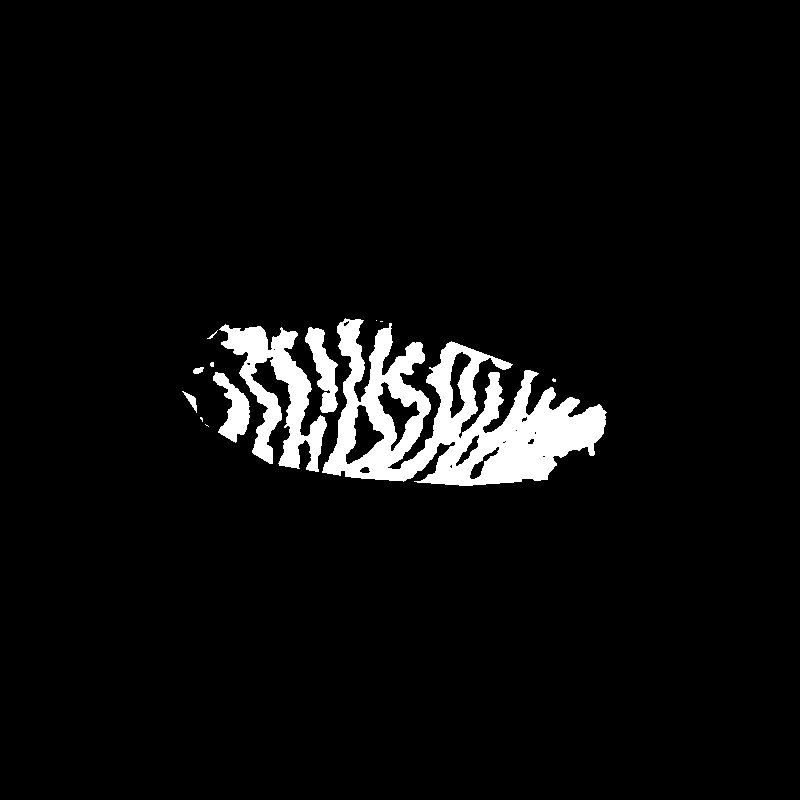

Supplement: S1 Raw images — (ZIP) [file pone.0270473.s008.zip › Unhealthy/unhealthy 40.jpg]

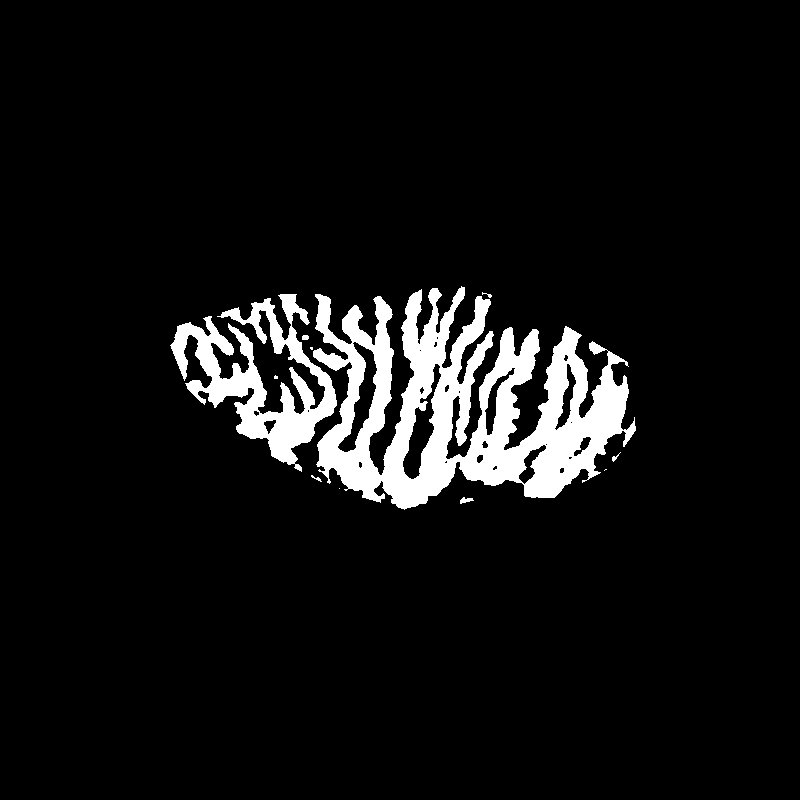

Supplement: S1 Raw images — (ZIP) [file pone.0270473.s008.zip › Unhealthy/unhealthy 41.jpg]

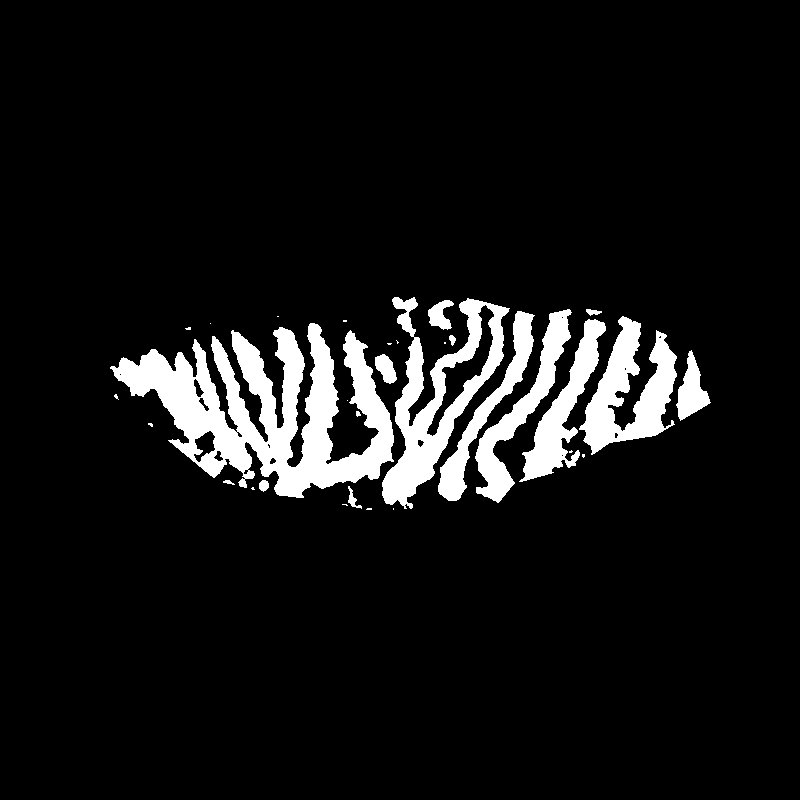

Supplement: S1 Raw images — (ZIP) [file pone.0270473.s008.zip › Unhealthy/unhealthy 42.jpg]

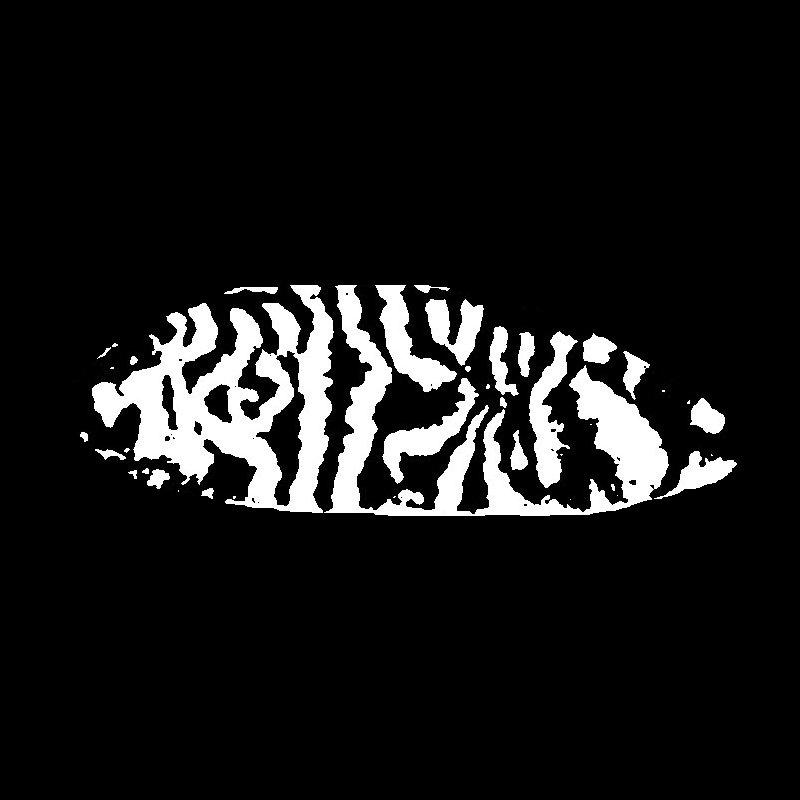

Supplement: S1 Raw images — (ZIP) [file pone.0270473.s008.zip › Unhealthy/unhealthy 43.jpg]

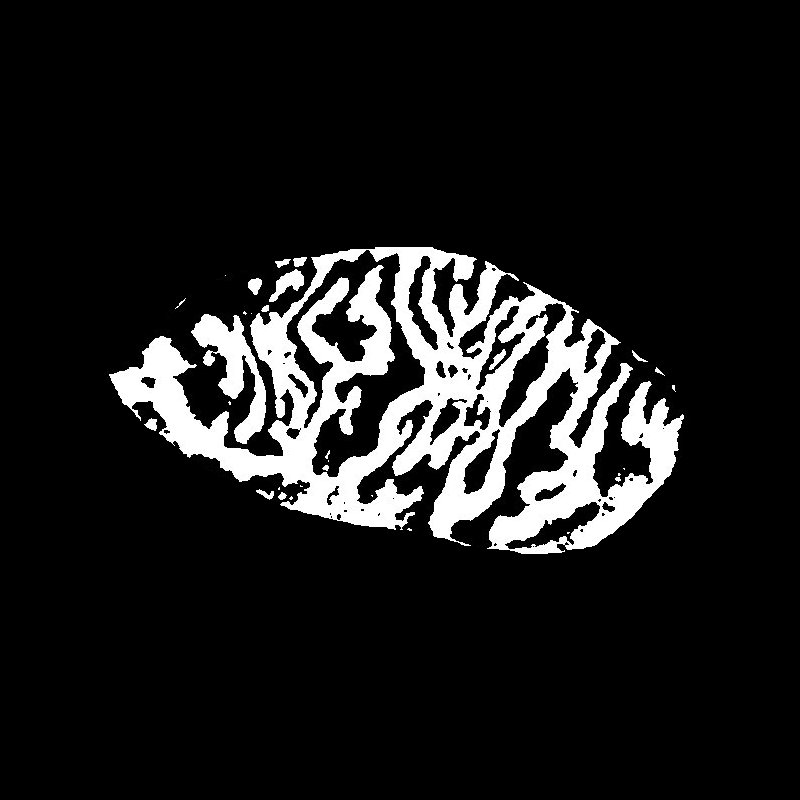

Supplement: S1 Raw images — (ZIP) [file pone.0270473.s008.zip › Unhealthy/unhealthy 44.jpg]

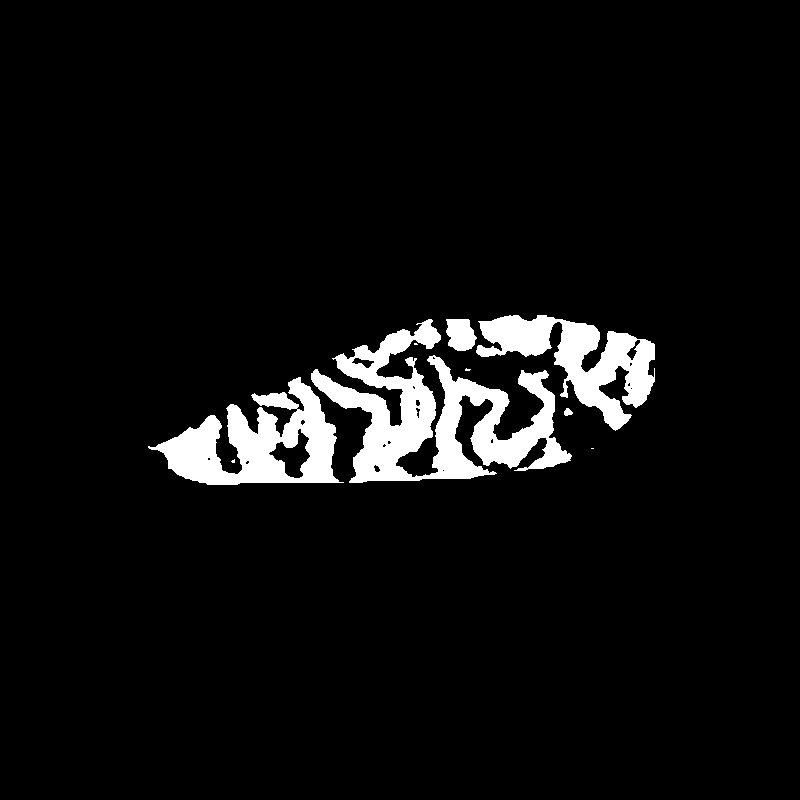

Supplement: S1 Raw images — (ZIP) [file pone.0270473.s008.zip › Unhealthy/unhealthy 45.jpg]

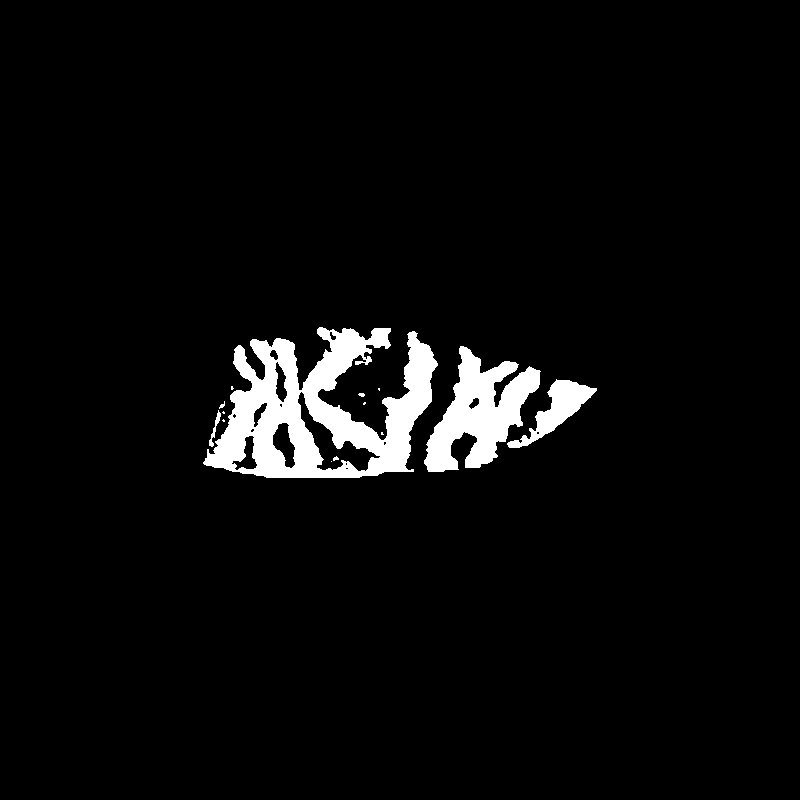

Supplement: S1 Raw images — (ZIP) [file pone.0270473.s008.zip › Unhealthy/unhealthy 46.jpg]

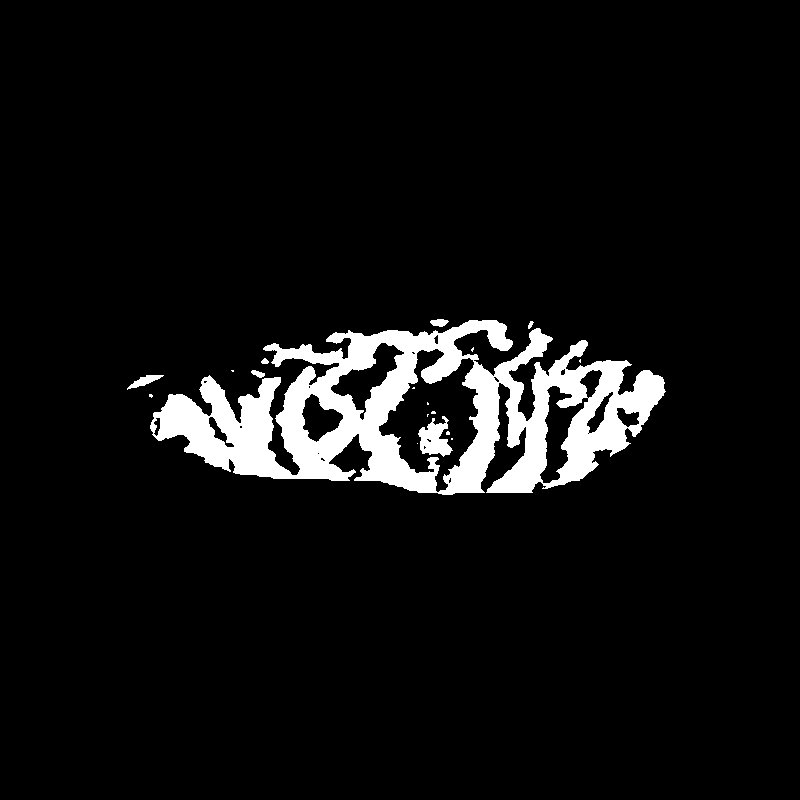

Supplement: S1 Raw images — (ZIP) [file pone.0270473.s008.zip › Unhealthy/unhealthy 47.jpg]

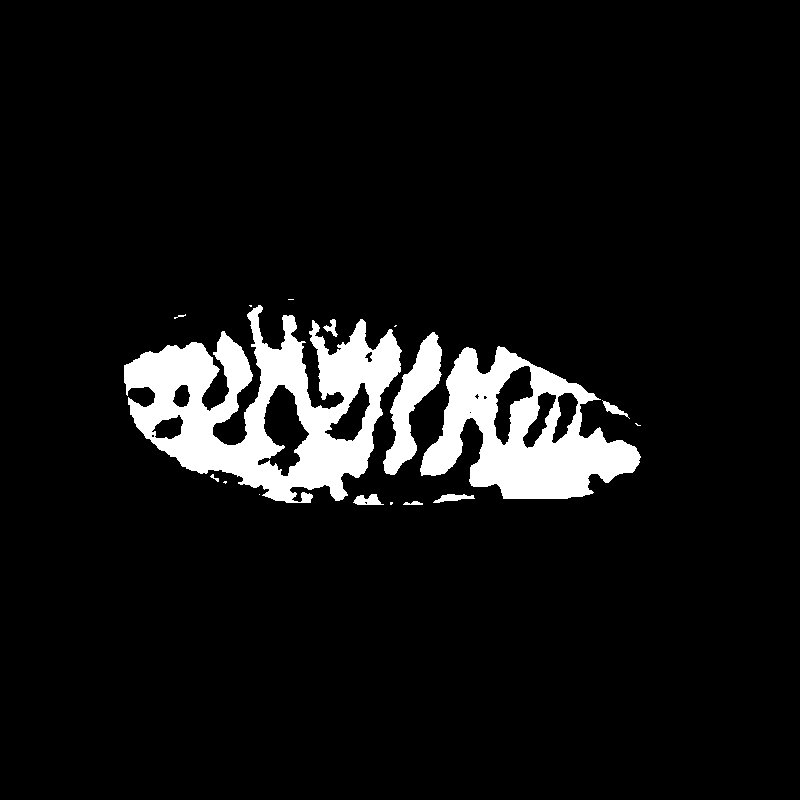

Supplement: S1 Raw images — (ZIP) [file pone.0270473.s008.zip › Unhealthy/unhealthy 5.jpg]

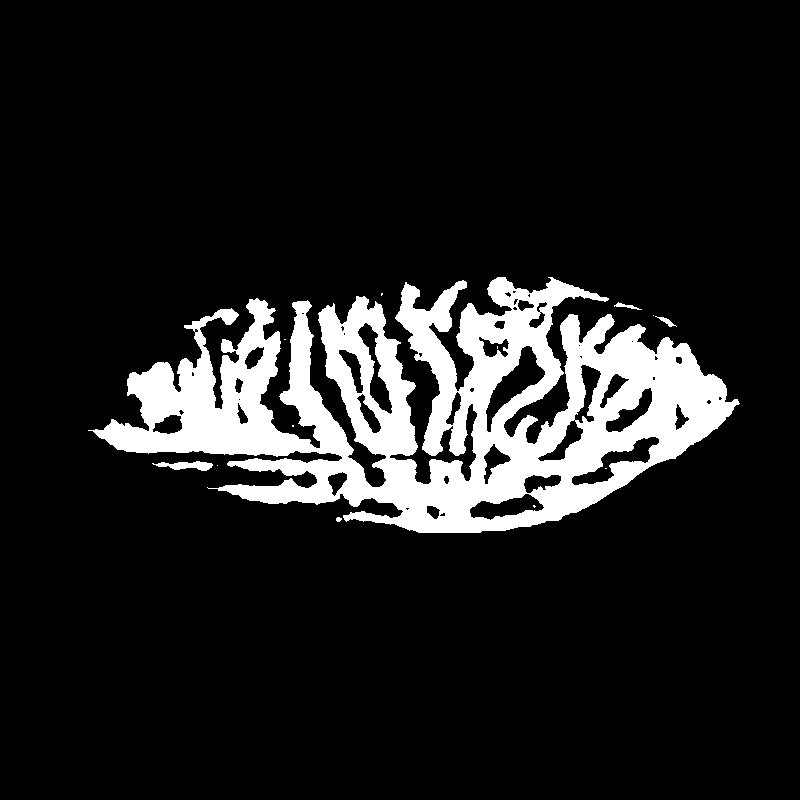

Supplement: S1 Raw images — (ZIP) [file pone.0270473.s008.zip › Unhealthy/unhealthy 6.jpg]

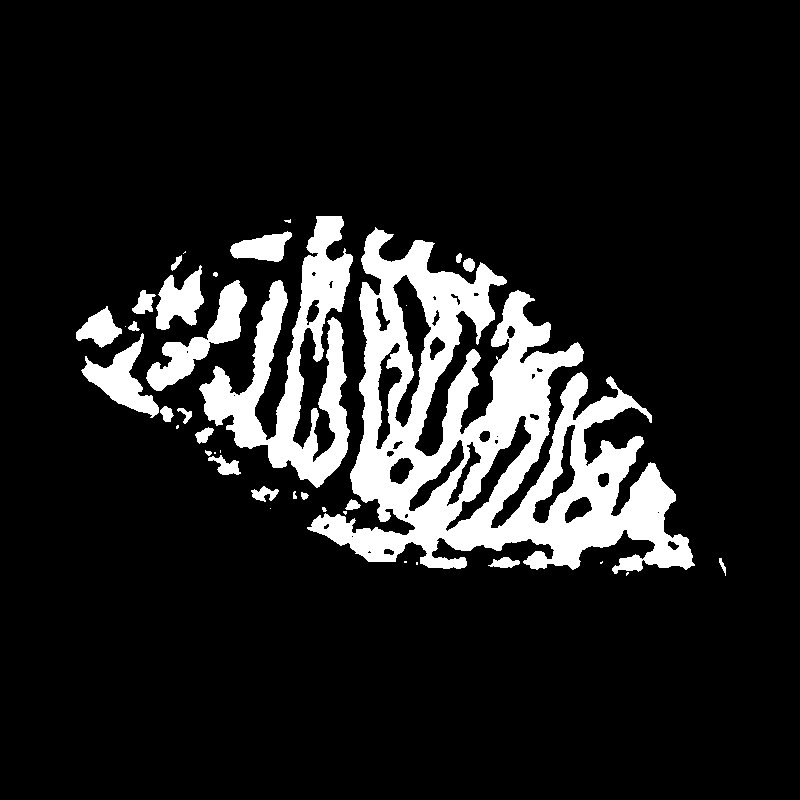

Supplement: S1 Raw images — (ZIP) [file pone.0270473.s008.zip › Unhealthy/unhealthy 7.jpg]

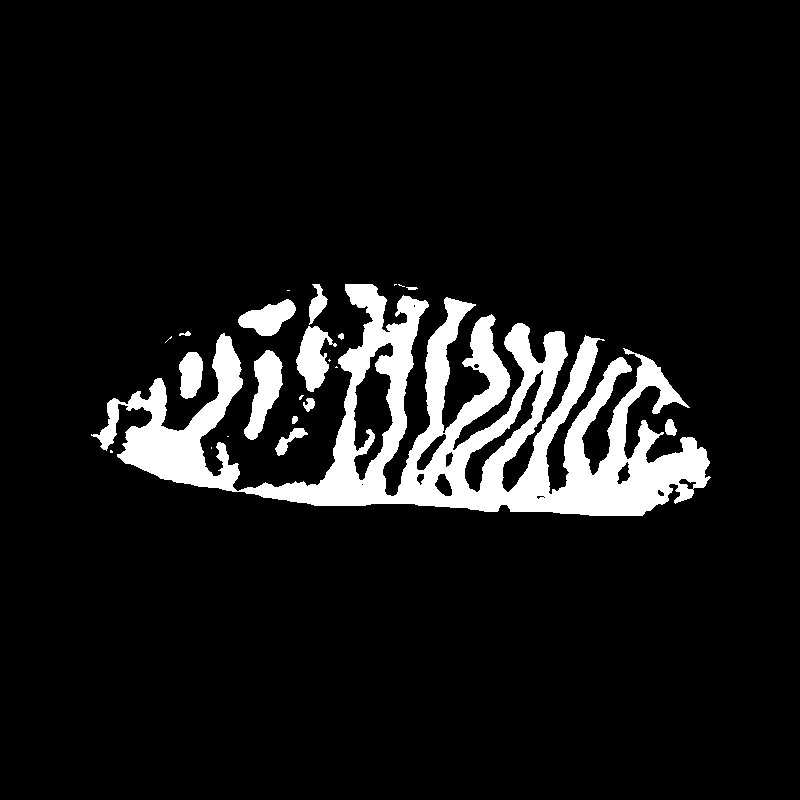

Supplement: S1 Raw images — (ZIP) [file pone.0270473.s008.zip › Unhealthy/unhealthy 8.jpg]

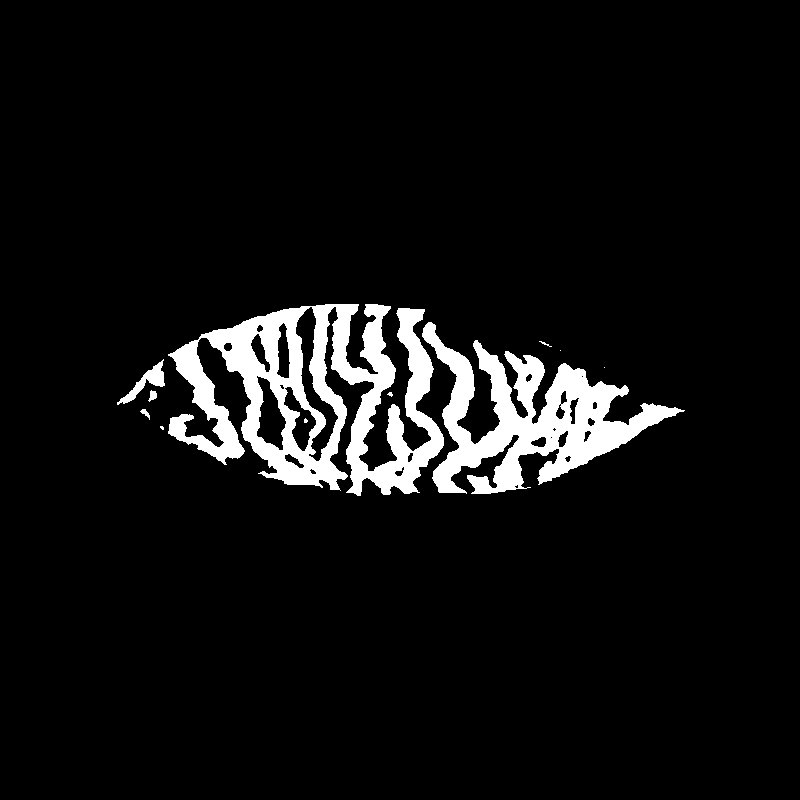

Supplement: S1 Raw images — (ZIP) [file pone.0270473.s008.zip › Unhealthy/unhealthy 9.jpg]
