# Supplementary figures and images for: 2D Short-Time Fourier Transform for local morphological analysis of meibomian gland images (part 1 of 2)
Source: PLoS One. 2022 Jun 24;17(6):e0270473. doi: 10.1371/journal.pone.0270473 (PMC9491703; doi:10.1371/journal.pone.0270473)

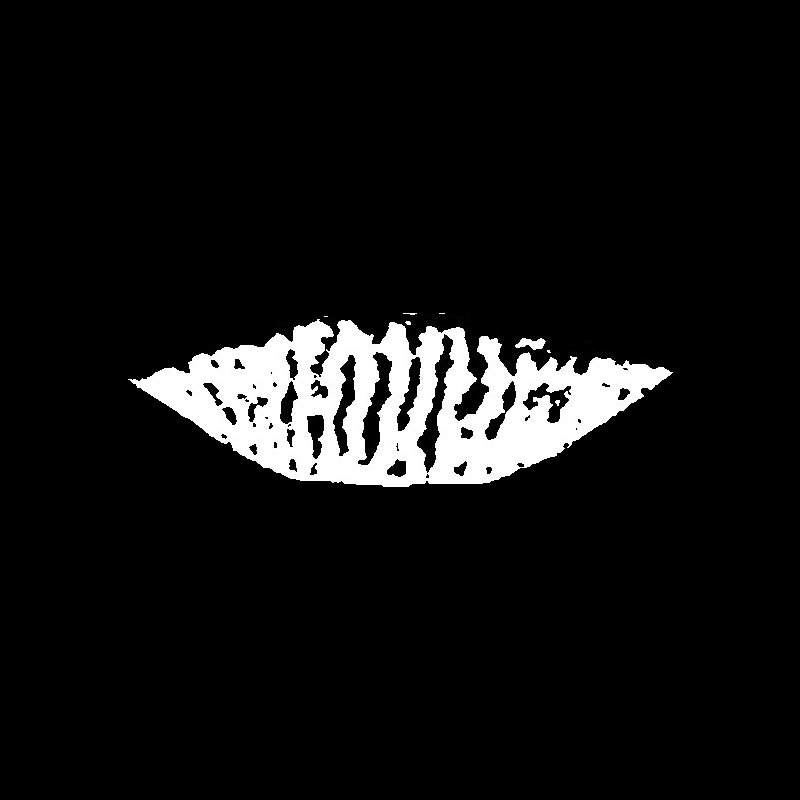

Supplement: S1 Raw images — (ZIP) [file pone.0270473.s008.zip › Healthy/healthy 1.jpg]

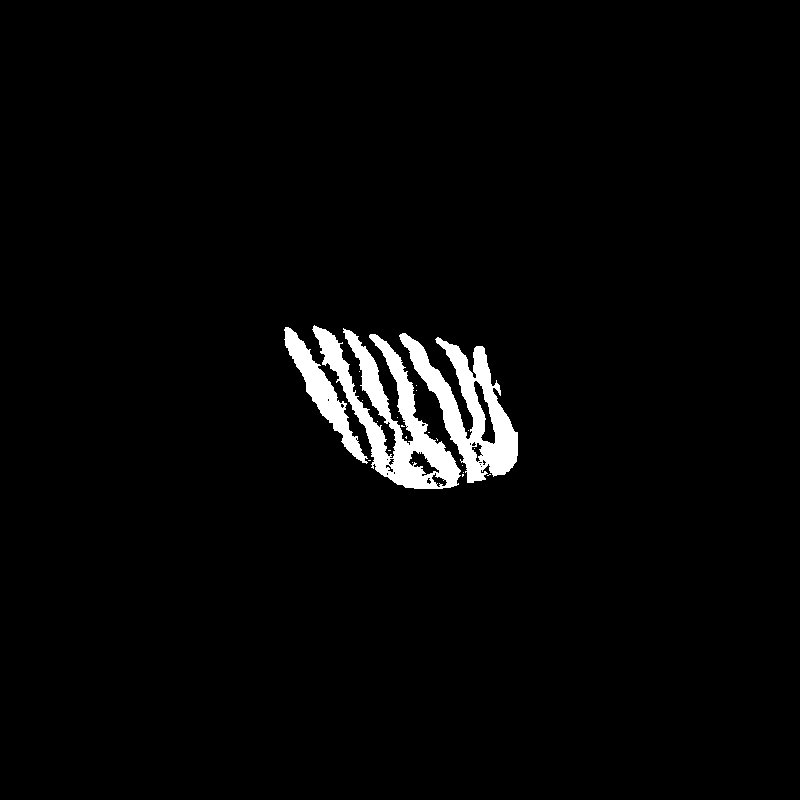

Supplement: S1 Raw images — (ZIP) [file pone.0270473.s008.zip › Healthy/healthy 10.jpg]

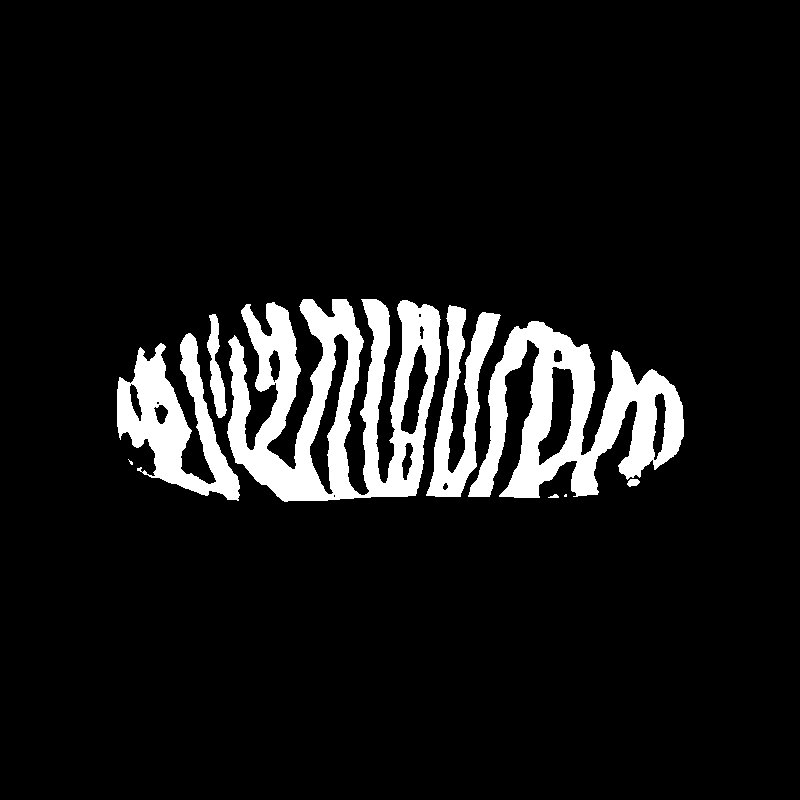

Supplement: S1 Raw images — (ZIP) [file pone.0270473.s008.zip › Healthy/healthy 11.jpg]

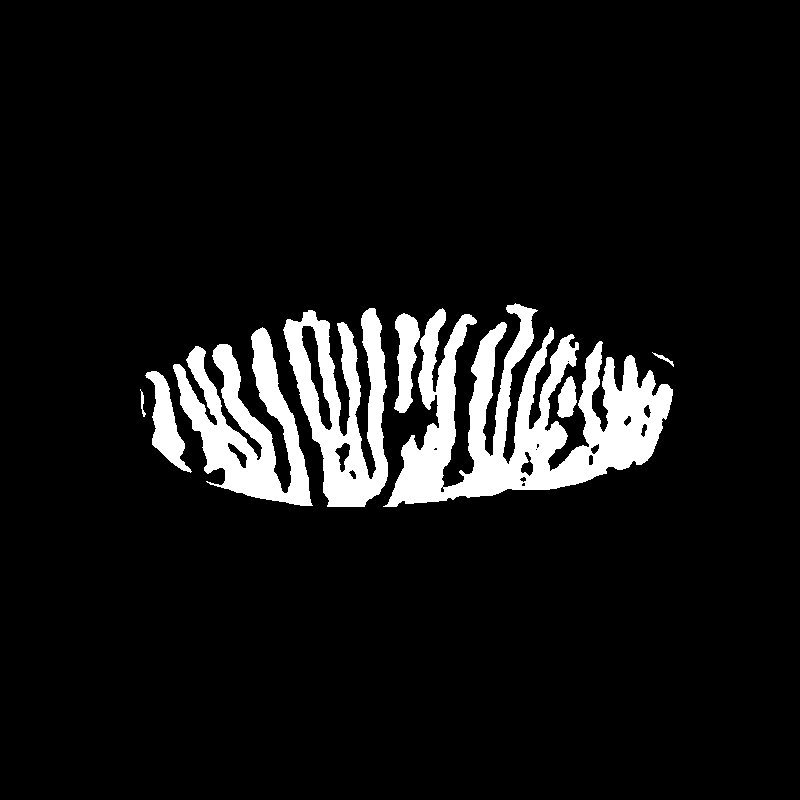

Supplement: S1 Raw images — (ZIP) [file pone.0270473.s008.zip › Healthy/healthy 12.jpg]

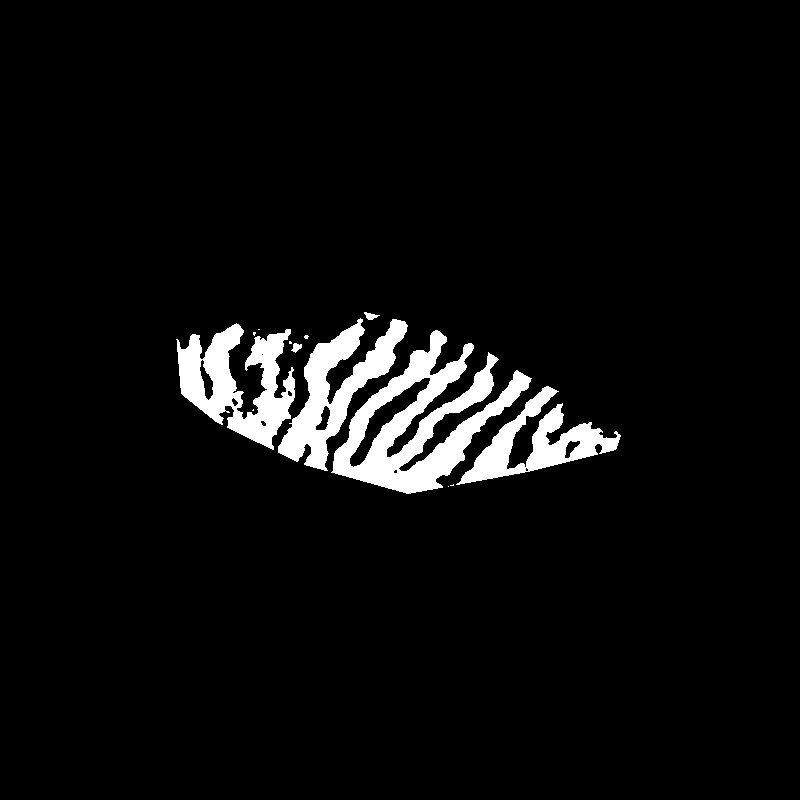

Supplement: S1 Raw images — (ZIP) [file pone.0270473.s008.zip › Healthy/healthy 13.jpg]

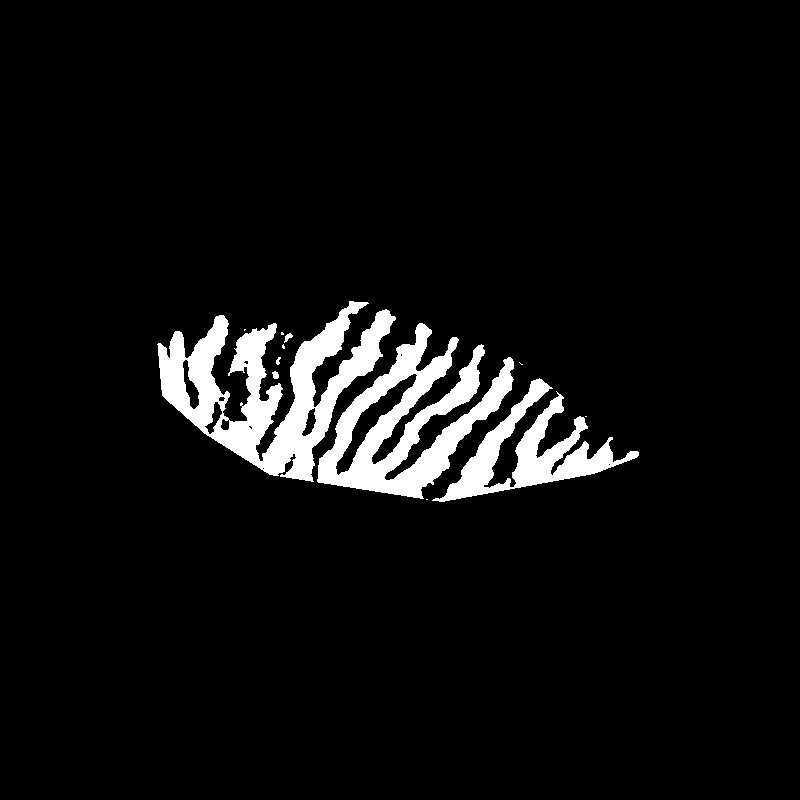

Supplement: S1 Raw images — (ZIP) [file pone.0270473.s008.zip › Healthy/healthy 14.jpg]

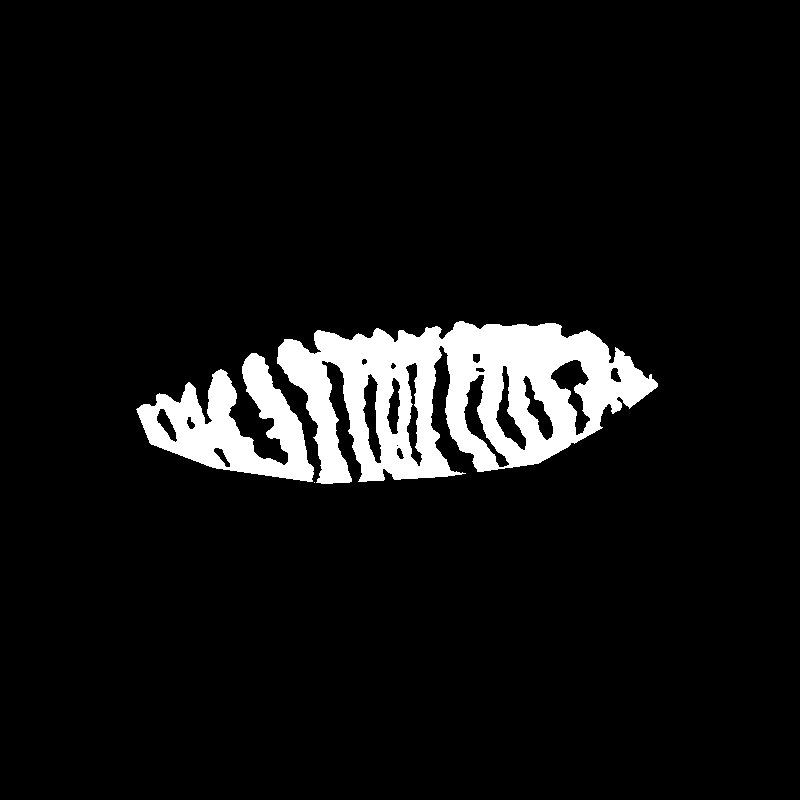

Supplement: S1 Raw images — (ZIP) [file pone.0270473.s008.zip › Healthy/healthy 15.jpg]

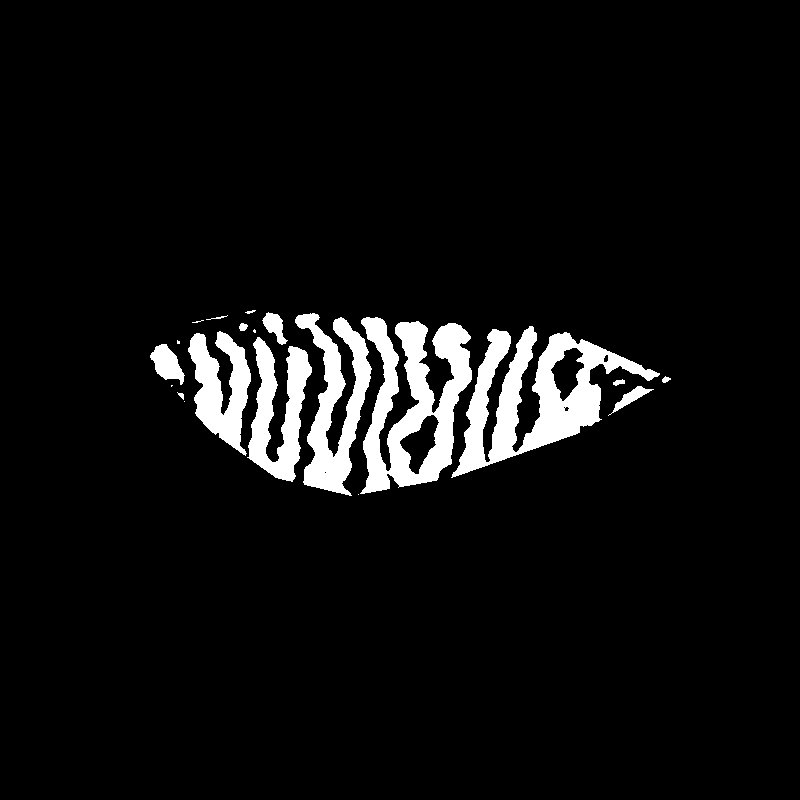

Supplement: S1 Raw images — (ZIP) [file pone.0270473.s008.zip › Healthy/healthy 16.jpg]

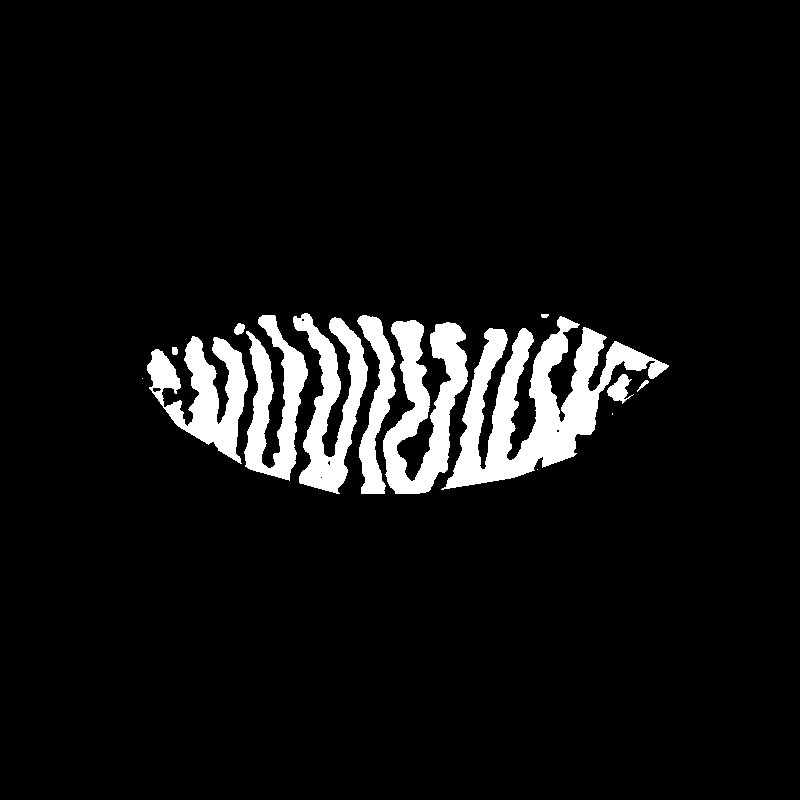

Supplement: S1 Raw images — (ZIP) [file pone.0270473.s008.zip › Healthy/healthy 17.jpg]

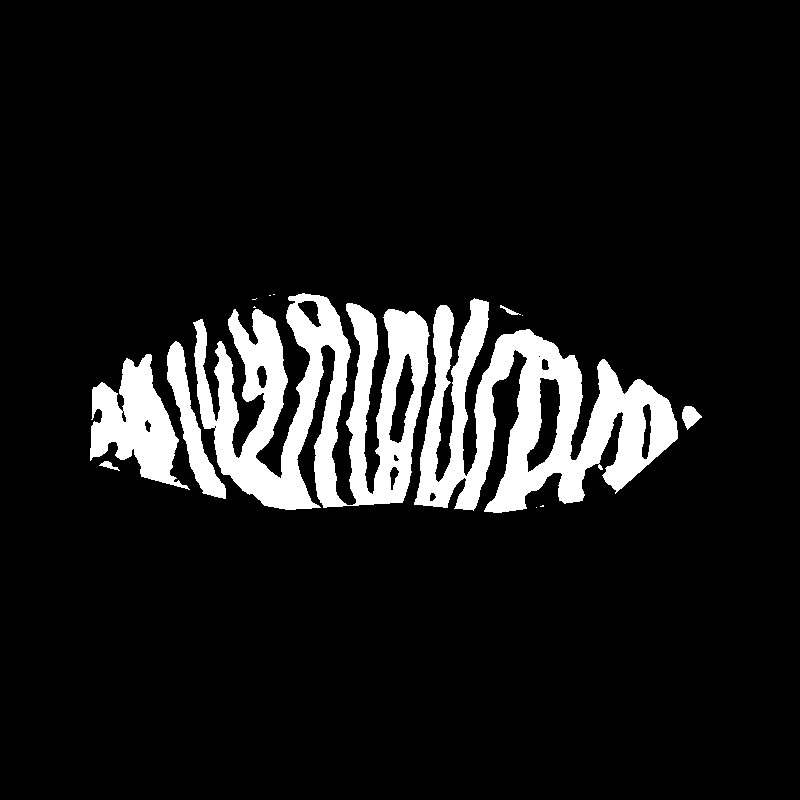

Supplement: S1 Raw images — (ZIP) [file pone.0270473.s008.zip › Healthy/healthy 18.jpg]

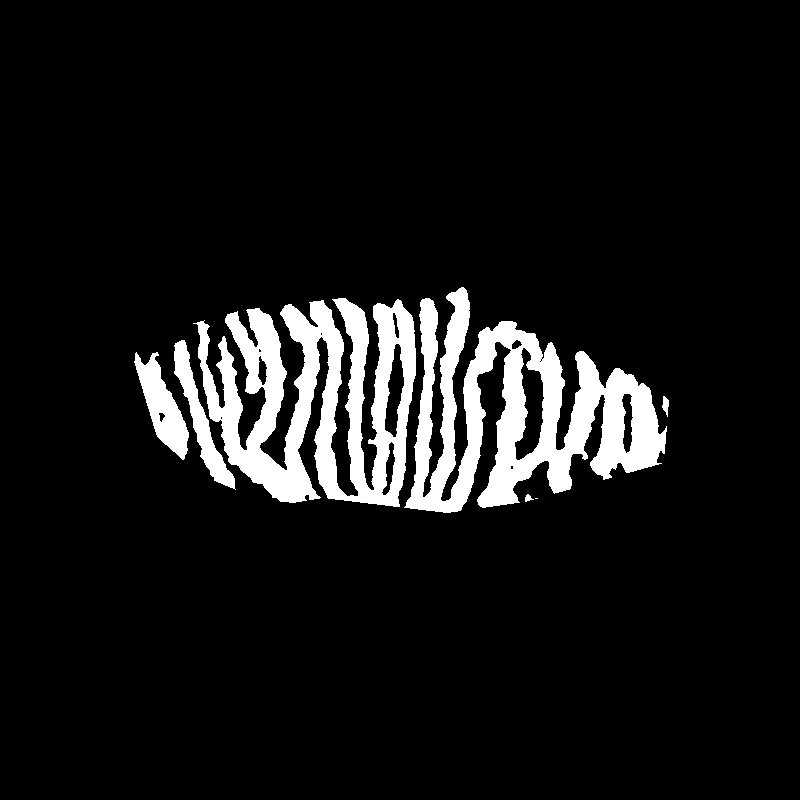

Supplement: S1 Raw images — (ZIP) [file pone.0270473.s008.zip › Healthy/healthy 19.jpg]

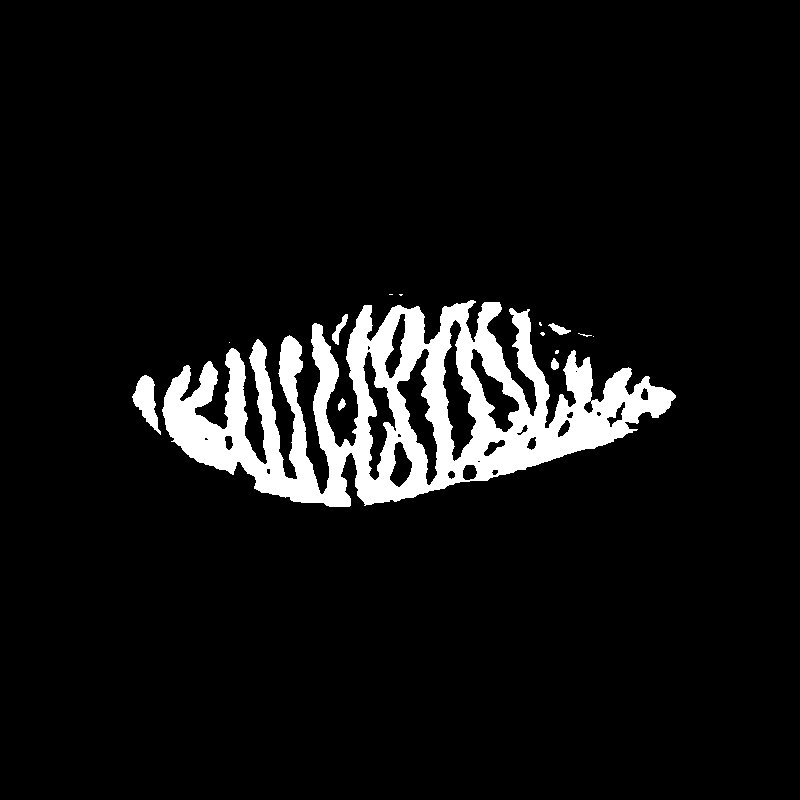

Supplement: S1 Raw images — (ZIP) [file pone.0270473.s008.zip › Healthy/healthy 2.jpg]

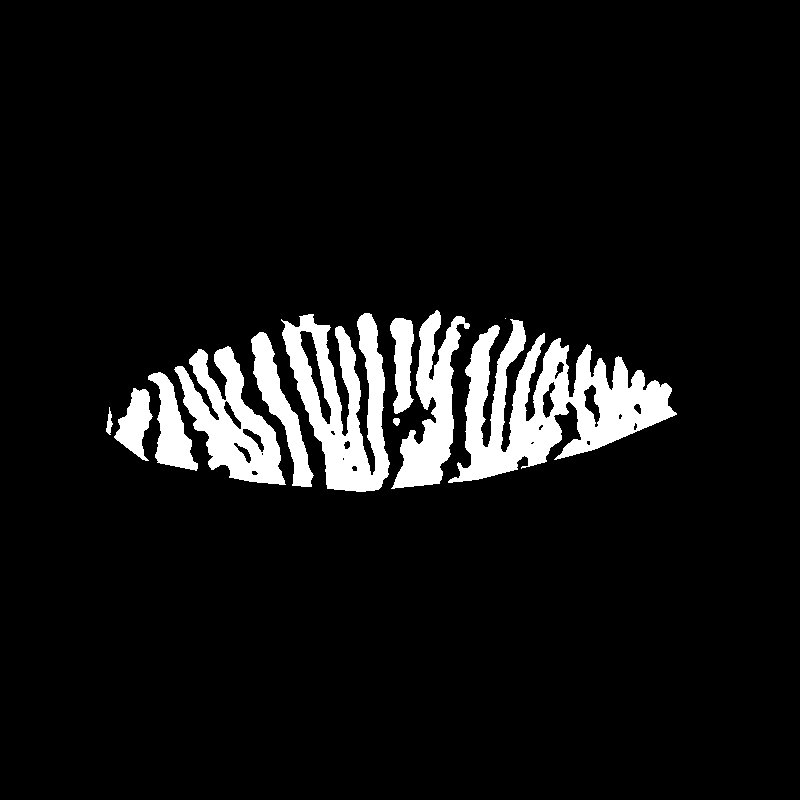

Supplement: S1 Raw images — (ZIP) [file pone.0270473.s008.zip › Healthy/healthy 20.jpg]

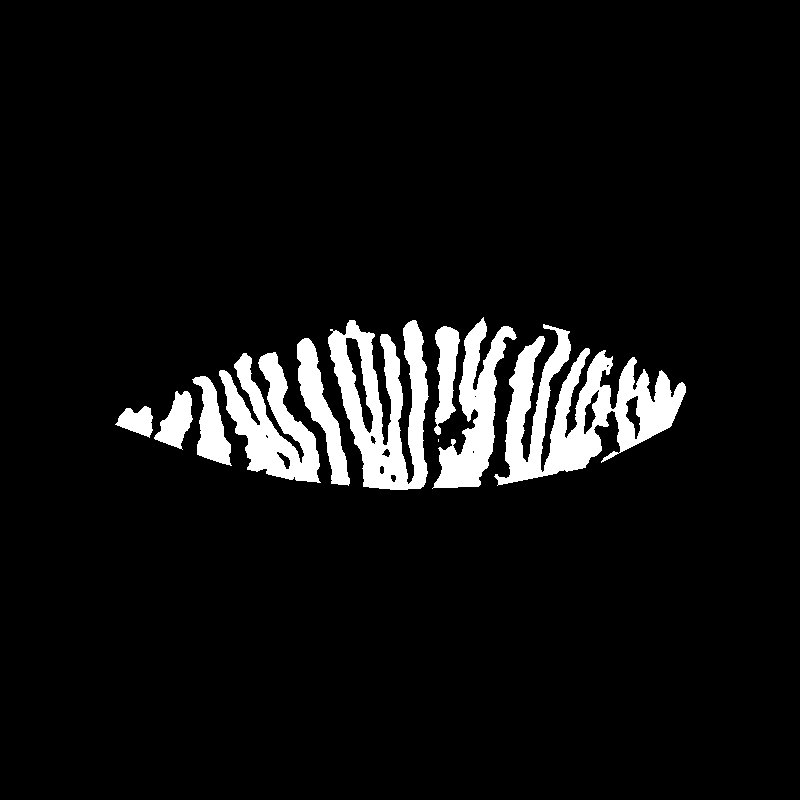

Supplement: S1 Raw images — (ZIP) [file pone.0270473.s008.zip › Healthy/healthy 21.jpg]

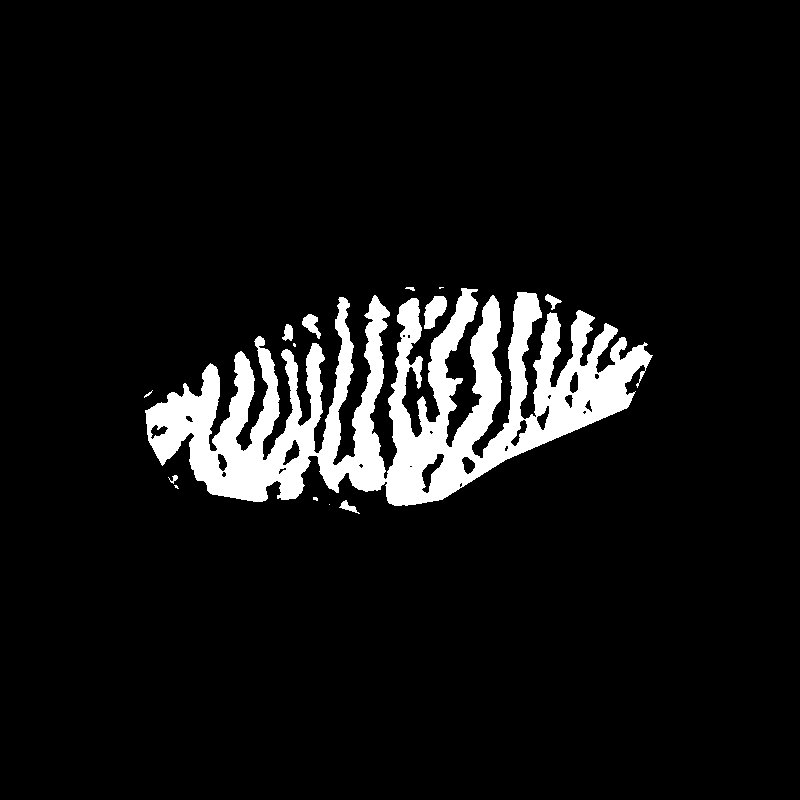

Supplement: S1 Raw images — (ZIP) [file pone.0270473.s008.zip › Healthy/healthy 22.jpg]

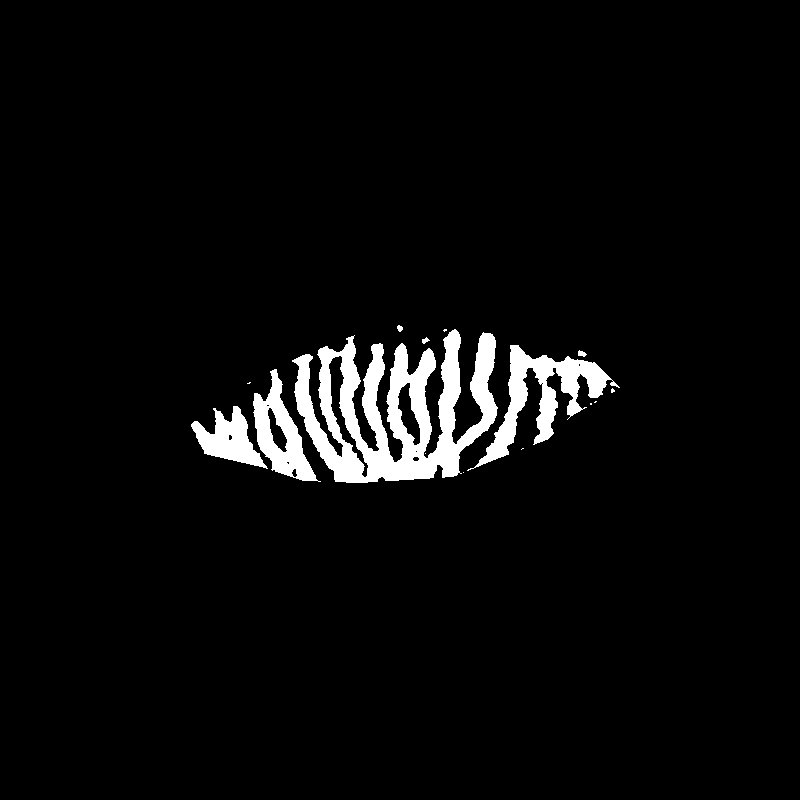

Supplement: S1 Raw images — (ZIP) [file pone.0270473.s008.zip › Healthy/healthy 23.jpg]

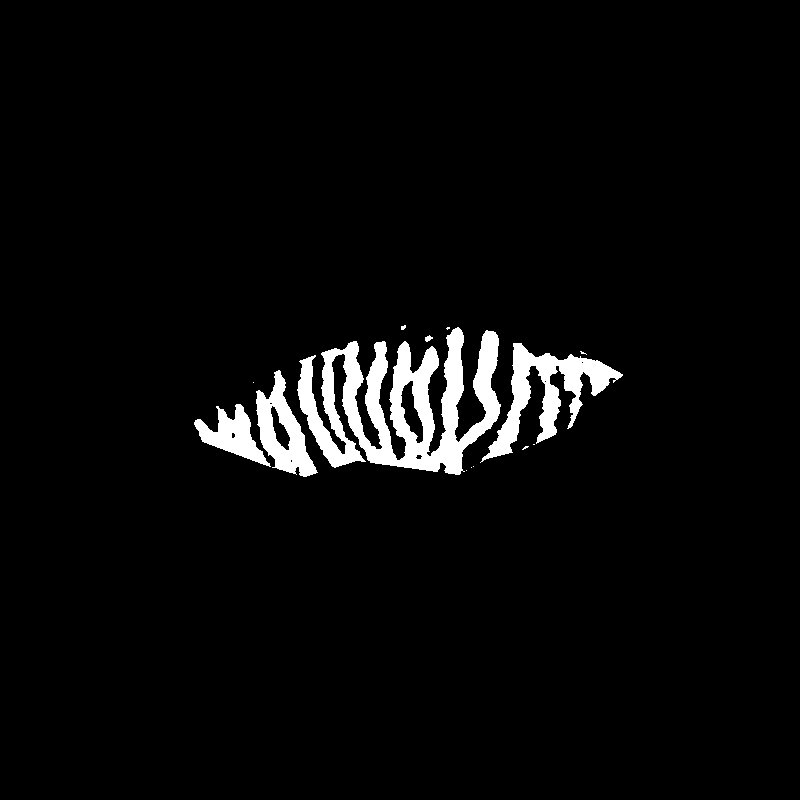

Supplement: S1 Raw images — (ZIP) [file pone.0270473.s008.zip › Healthy/healthy 24.jpg]

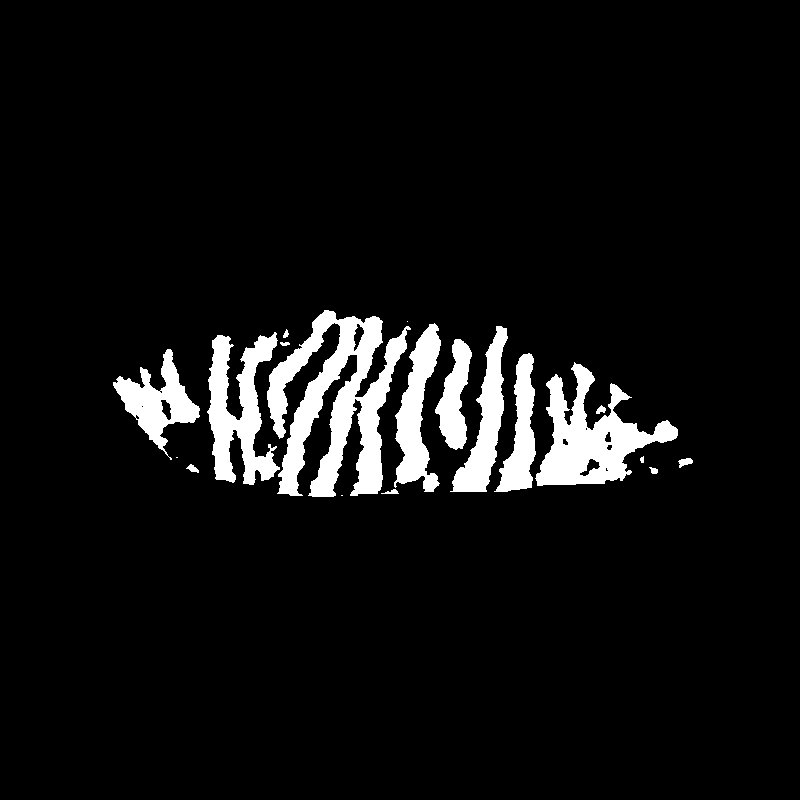

Supplement: S1 Raw images — (ZIP) [file pone.0270473.s008.zip › Healthy/healthy 3.jpg]

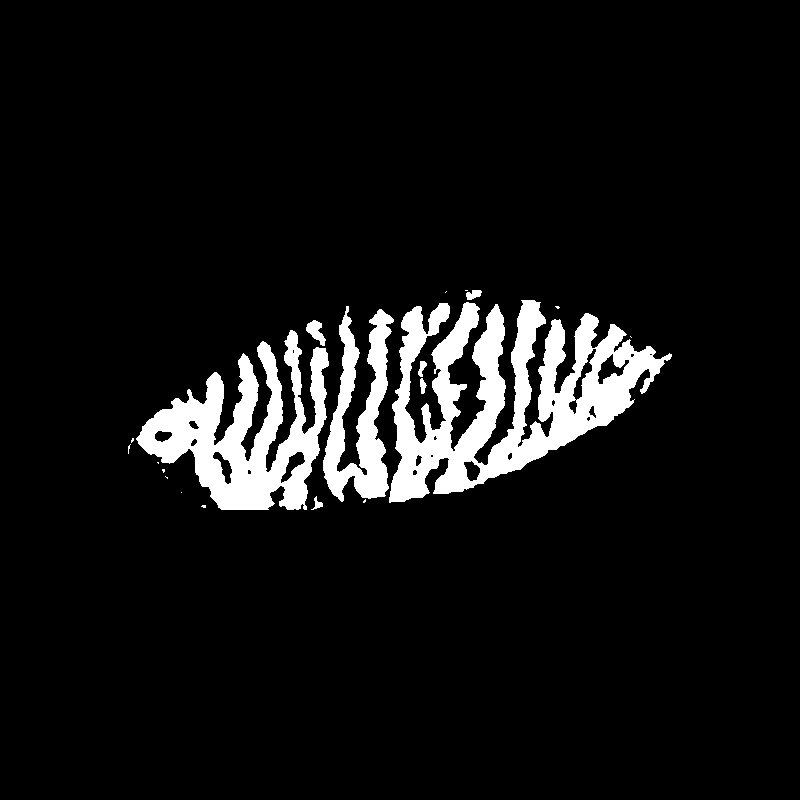

Supplement: S1 Raw images — (ZIP) [file pone.0270473.s008.zip › Healthy/healthy 4.jpg]

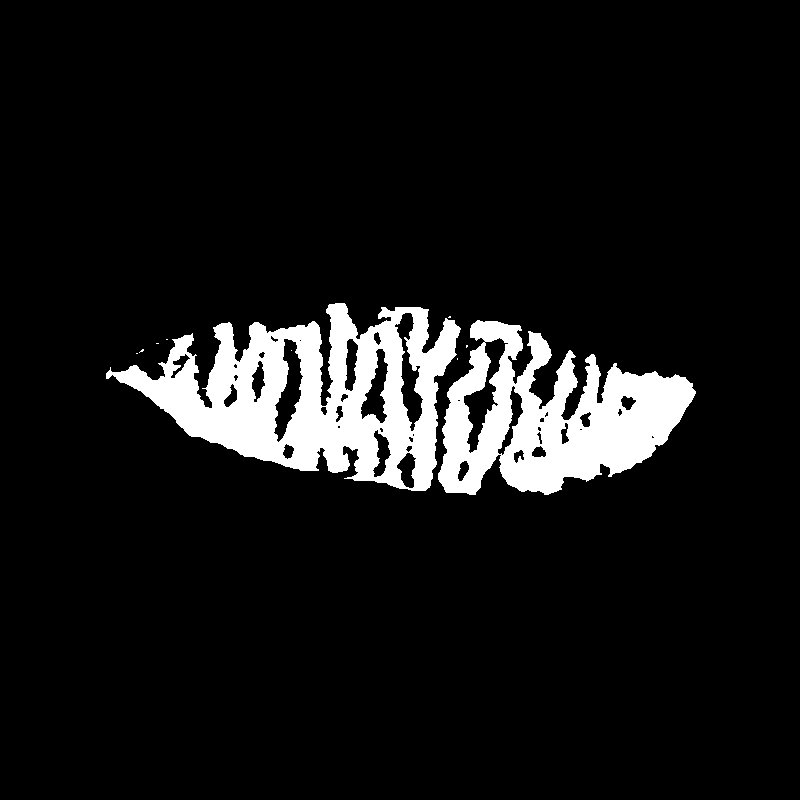

Supplement: S1 Raw images — (ZIP) [file pone.0270473.s008.zip › Healthy/healthy 5.jpg]

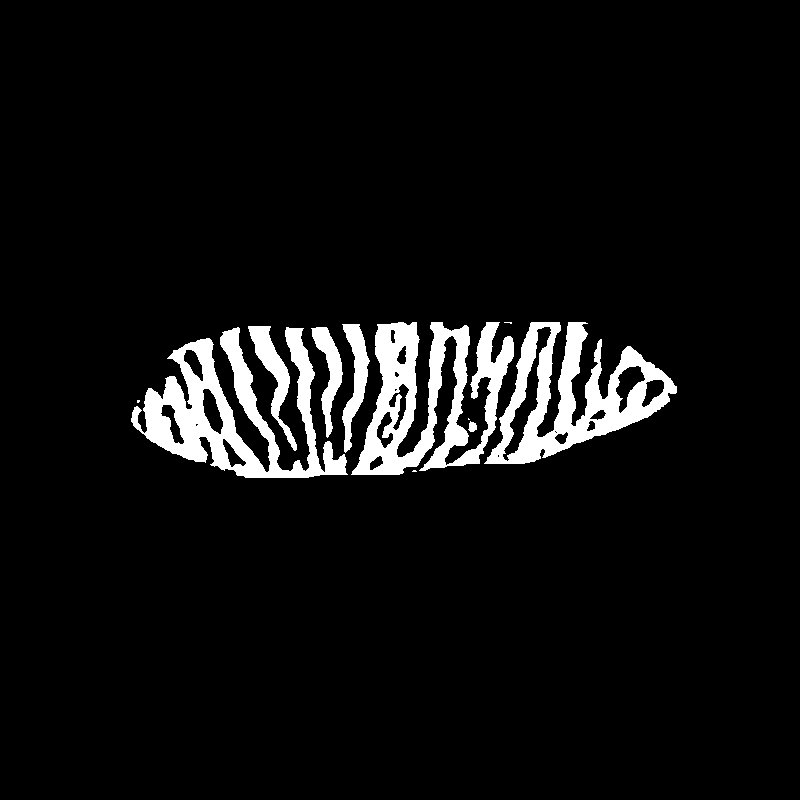

Supplement: S1 Raw images — (ZIP) [file pone.0270473.s008.zip › Healthy/healthy 6.jpg]

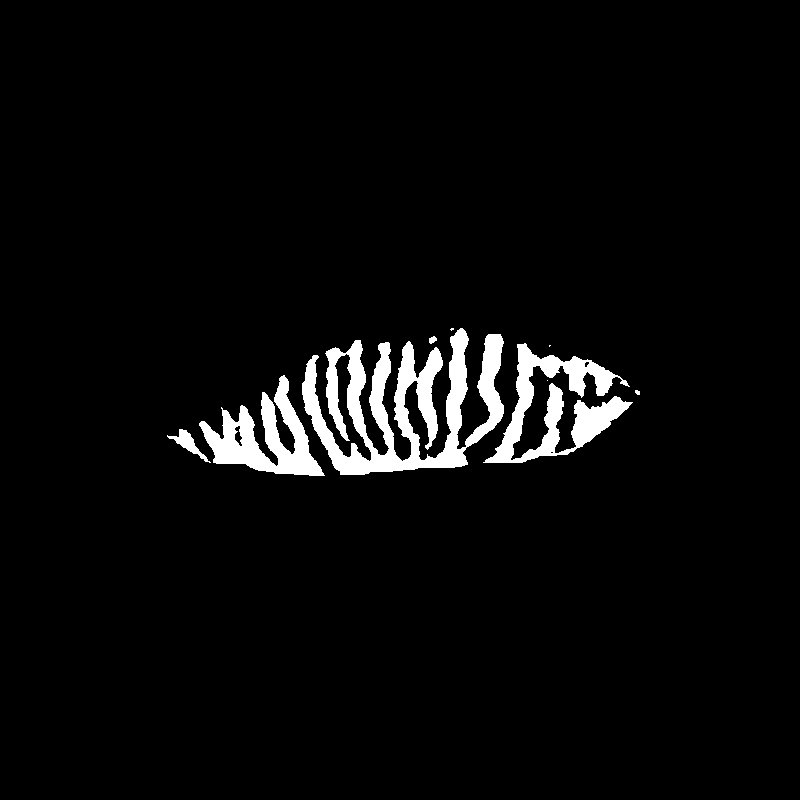

Supplement: S1 Raw images — (ZIP) [file pone.0270473.s008.zip › Healthy/healthy 7.jpg]

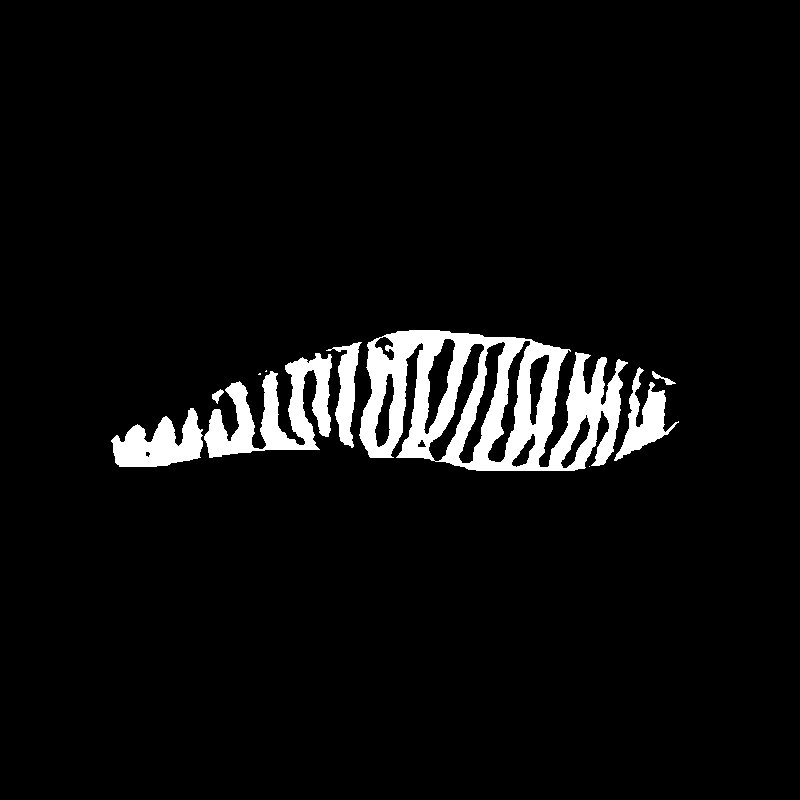

Supplement: S1 Raw images — (ZIP) [file pone.0270473.s008.zip › Healthy/healthy 8.jpg]

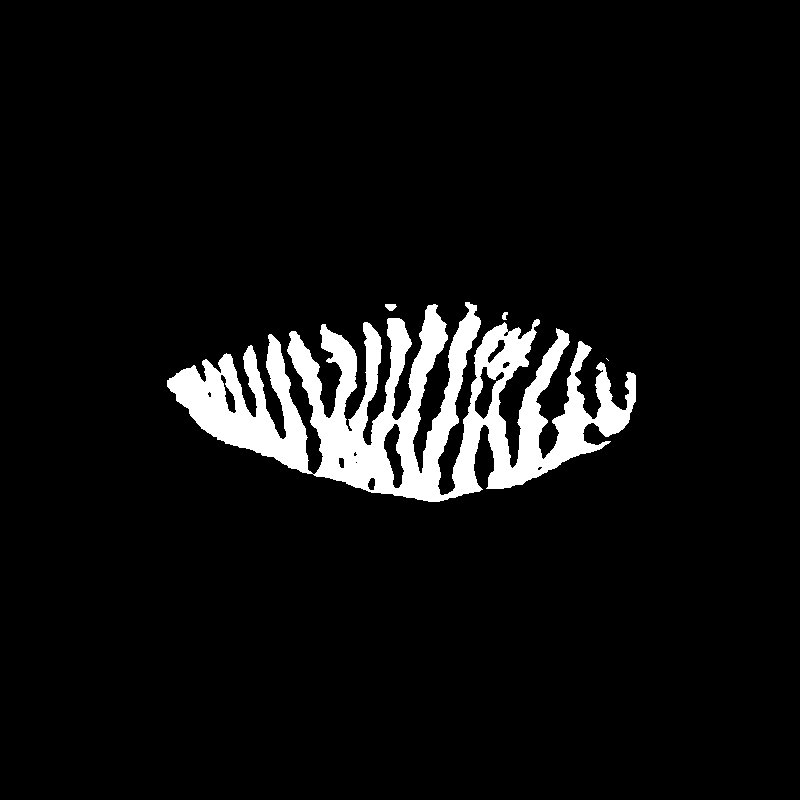

Supplement: S1 Raw images — (ZIP) [file pone.0270473.s008.zip › Healthy/healthy 9.jpg]

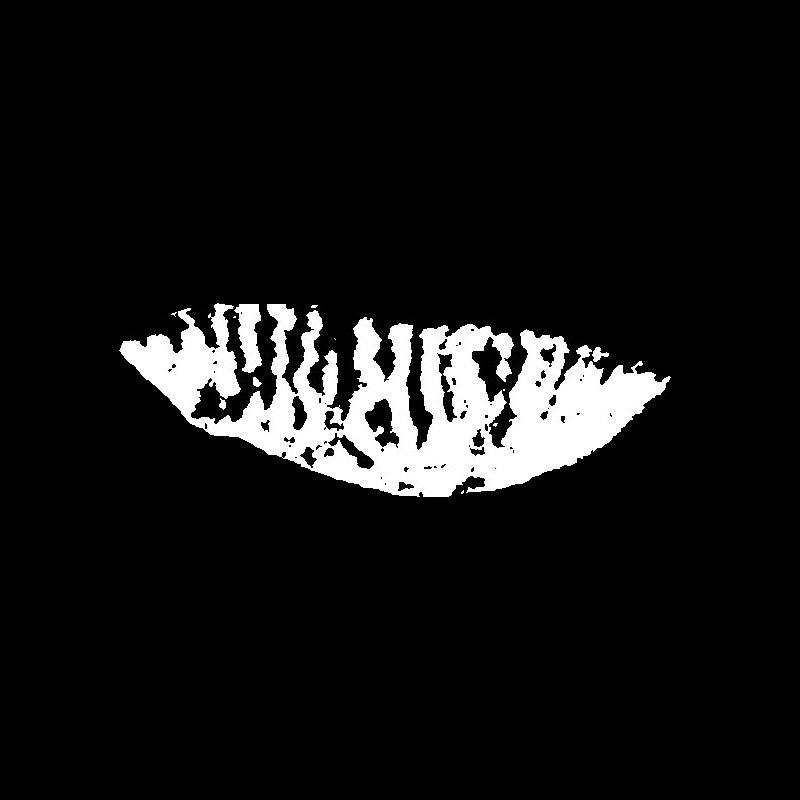

Supplement: S1 Raw images — (ZIP) [file pone.0270473.s008.zip › Intermediate/intermediate 1.jpg]

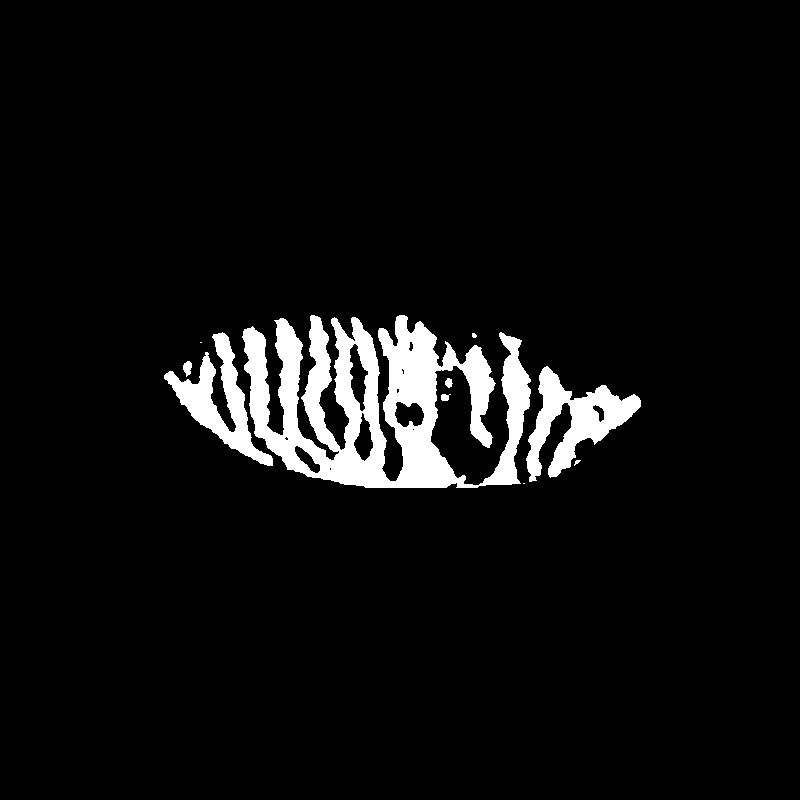

Supplement: S1 Raw images — (ZIP) [file pone.0270473.s008.zip › Intermediate/intermediate 10.jpg]

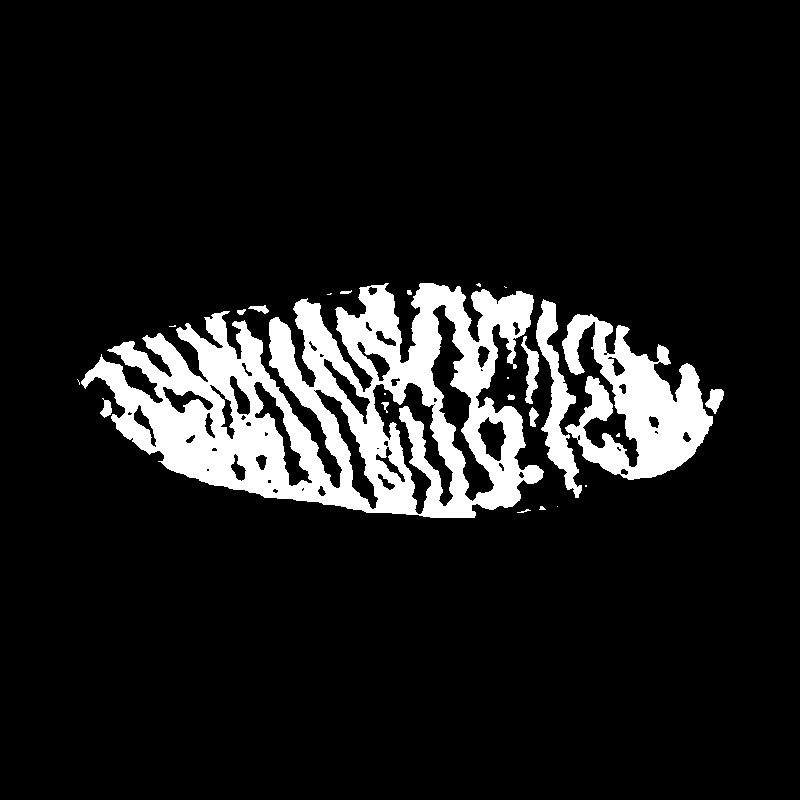

Supplement: S1 Raw images — (ZIP) [file pone.0270473.s008.zip › Intermediate/intermediate 11.jpg]

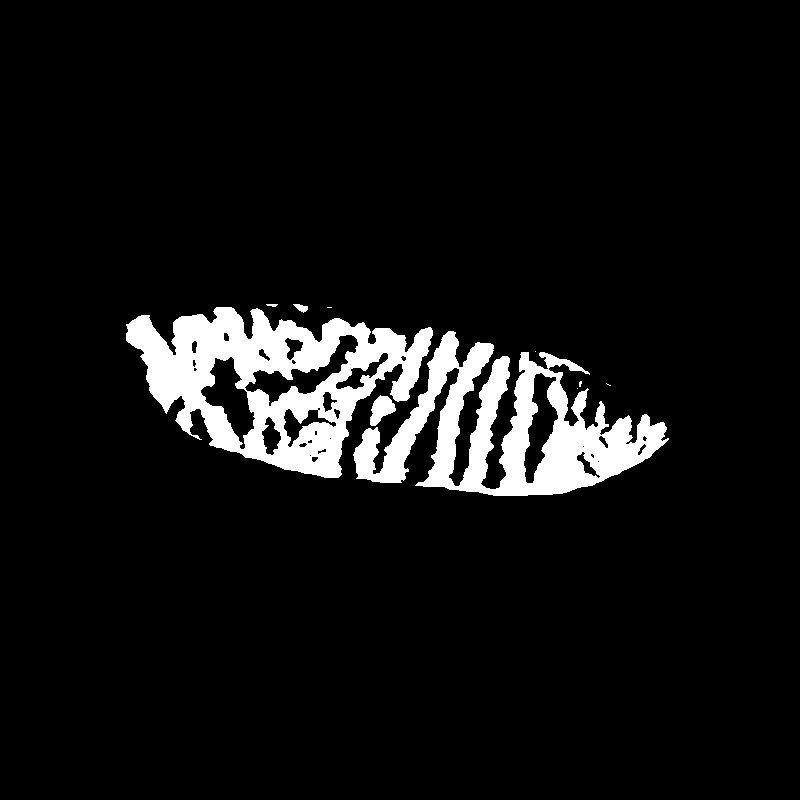

Supplement: S1 Raw images — (ZIP) [file pone.0270473.s008.zip › Intermediate/intermediate 12.jpg]

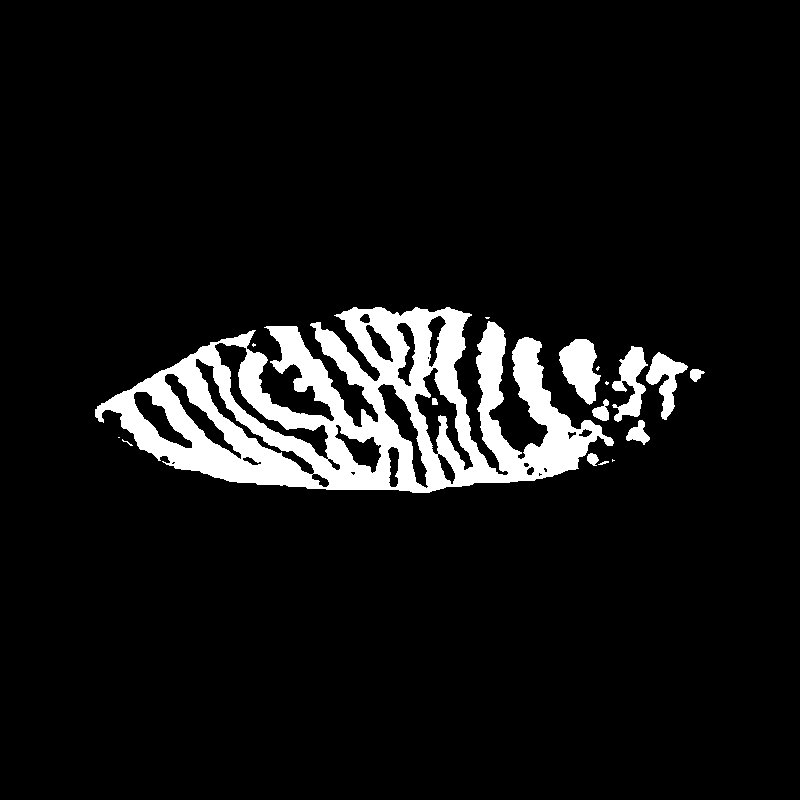

Supplement: S1 Raw images — (ZIP) [file pone.0270473.s008.zip › Intermediate/intermediate 13.jpg]

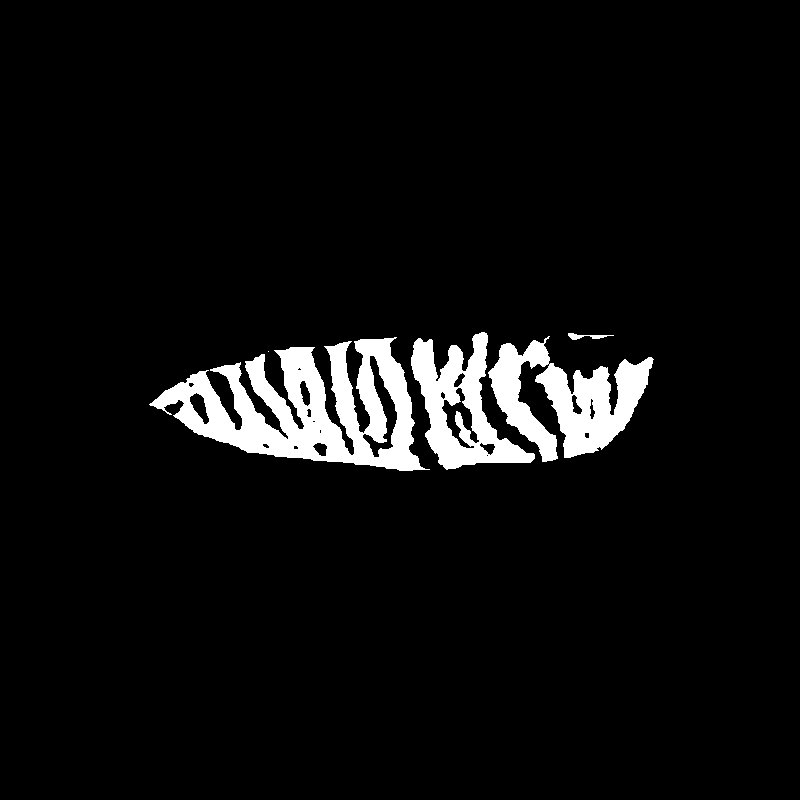

Supplement: S1 Raw images — (ZIP) [file pone.0270473.s008.zip › Intermediate/intermediate 14.jpg]

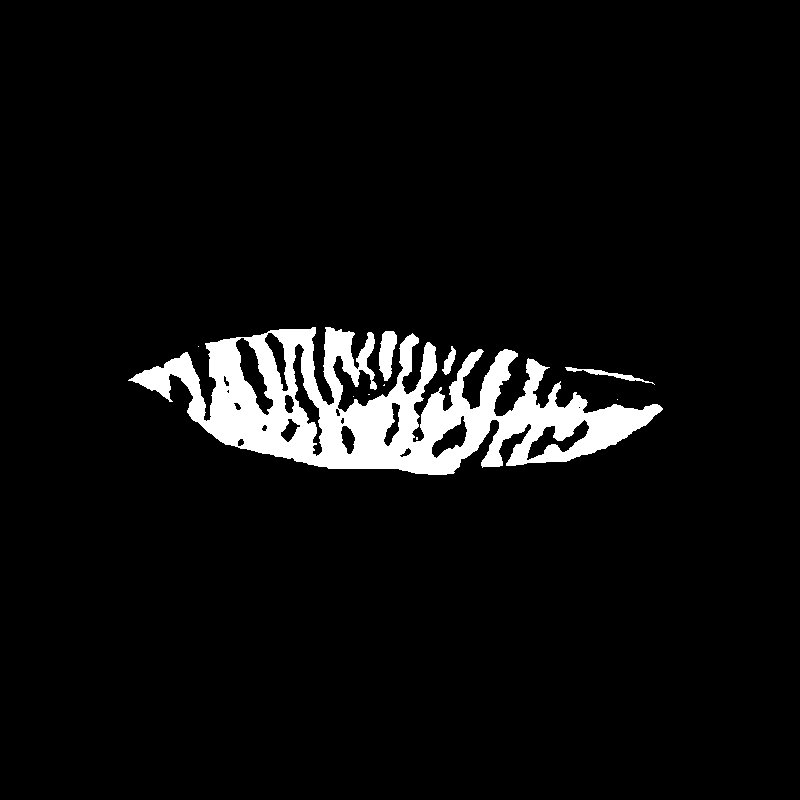

Supplement: S1 Raw images — (ZIP) [file pone.0270473.s008.zip › Intermediate/intermediate 15.jpg]

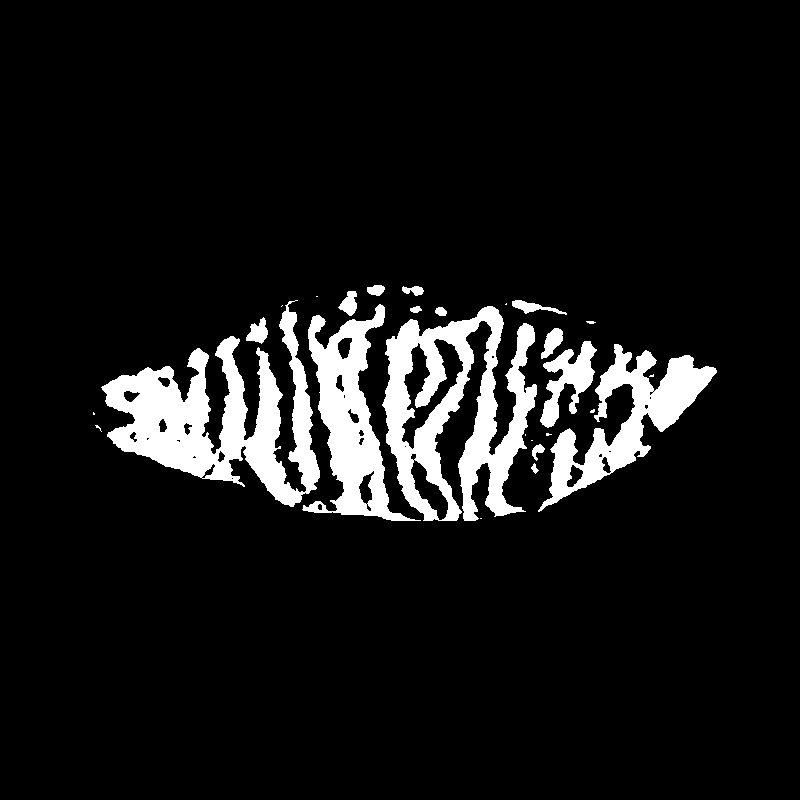

Supplement: S1 Raw images — (ZIP) [file pone.0270473.s008.zip › Intermediate/intermediate 16.jpg]

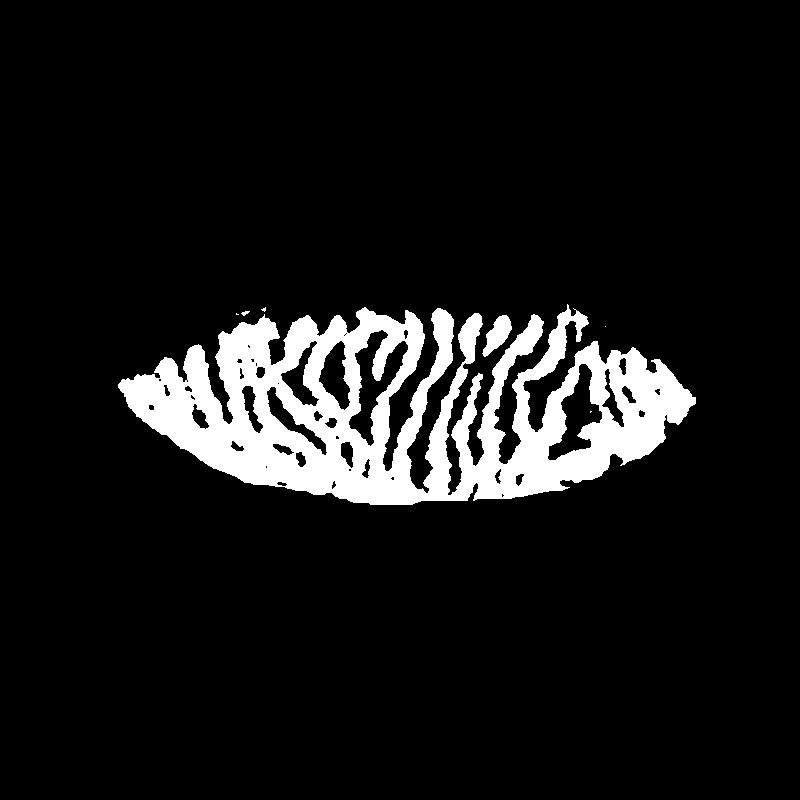

Supplement: S1 Raw images — (ZIP) [file pone.0270473.s008.zip › Intermediate/intermediate 17.jpg]

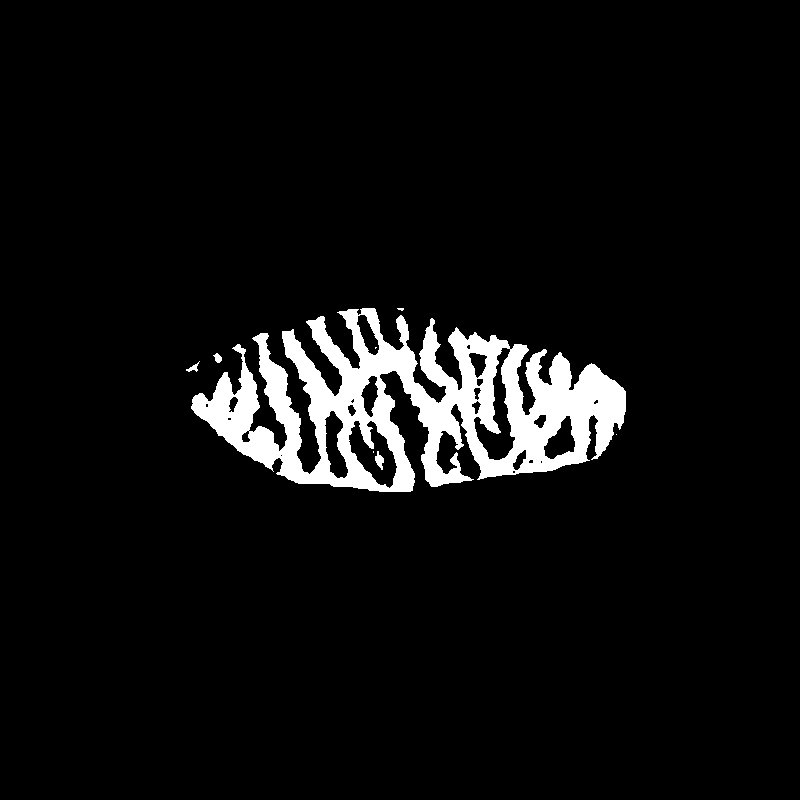

Supplement: S1 Raw images — (ZIP) [file pone.0270473.s008.zip › Intermediate/intermediate 18.jpg]

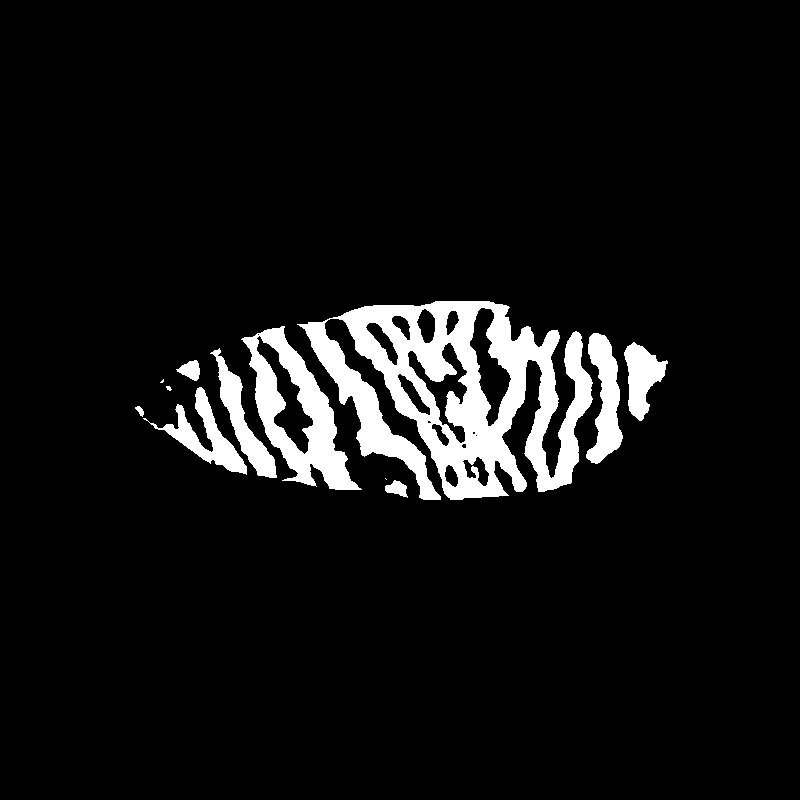

Supplement: S1 Raw images — (ZIP) [file pone.0270473.s008.zip › Intermediate/intermediate 19.jpg]

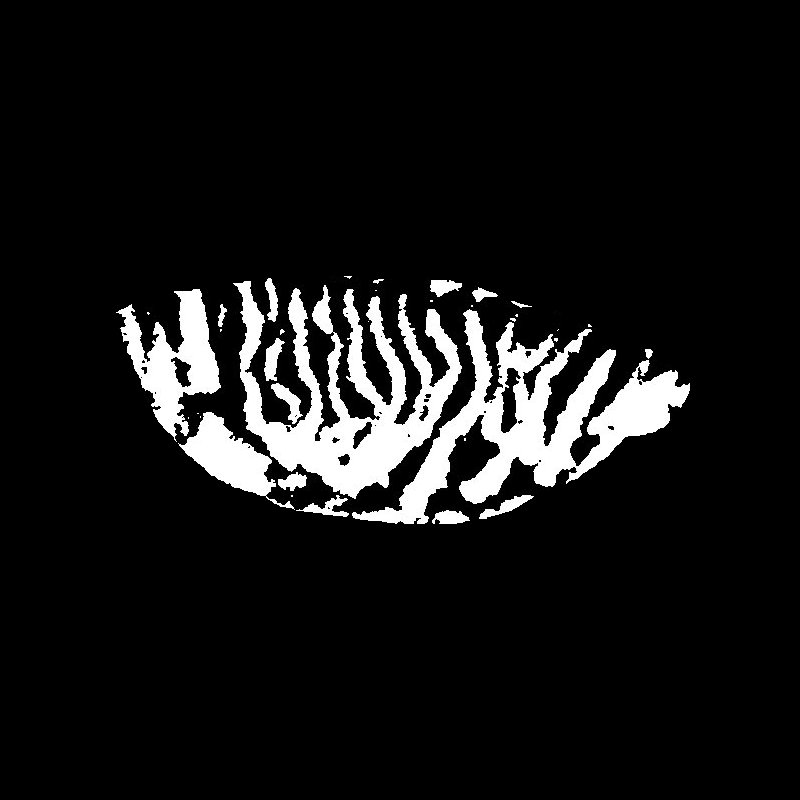

Supplement: S1 Raw images — (ZIP) [file pone.0270473.s008.zip › Intermediate/intermediate 2.jpg]

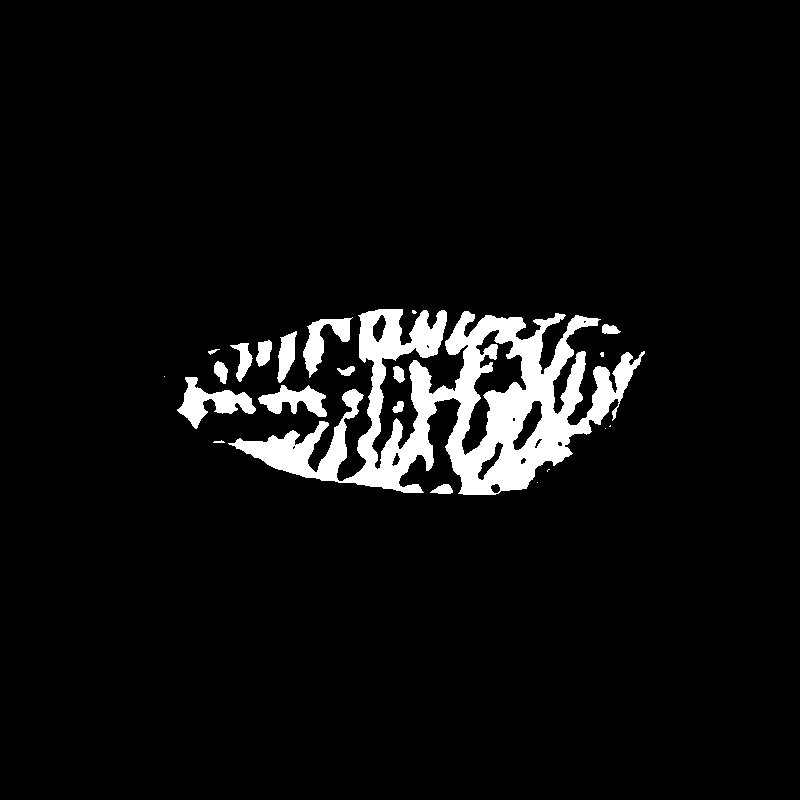

Supplement: S1 Raw images — (ZIP) [file pone.0270473.s008.zip › Intermediate/intermediate 20.jpg]

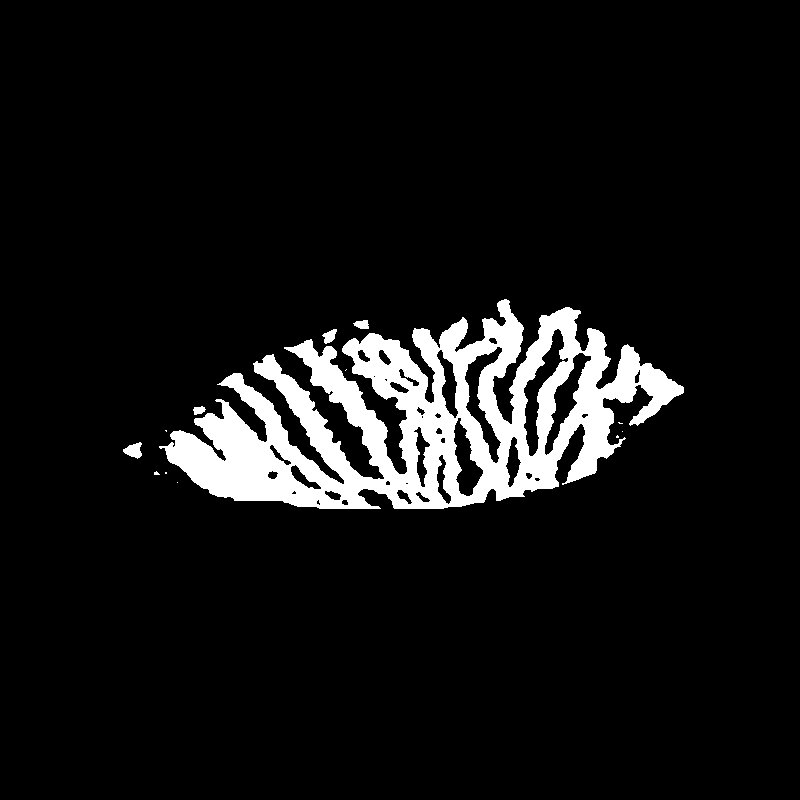

Supplement: S1 Raw images — (ZIP) [file pone.0270473.s008.zip › Intermediate/intermediate 21.jpg]

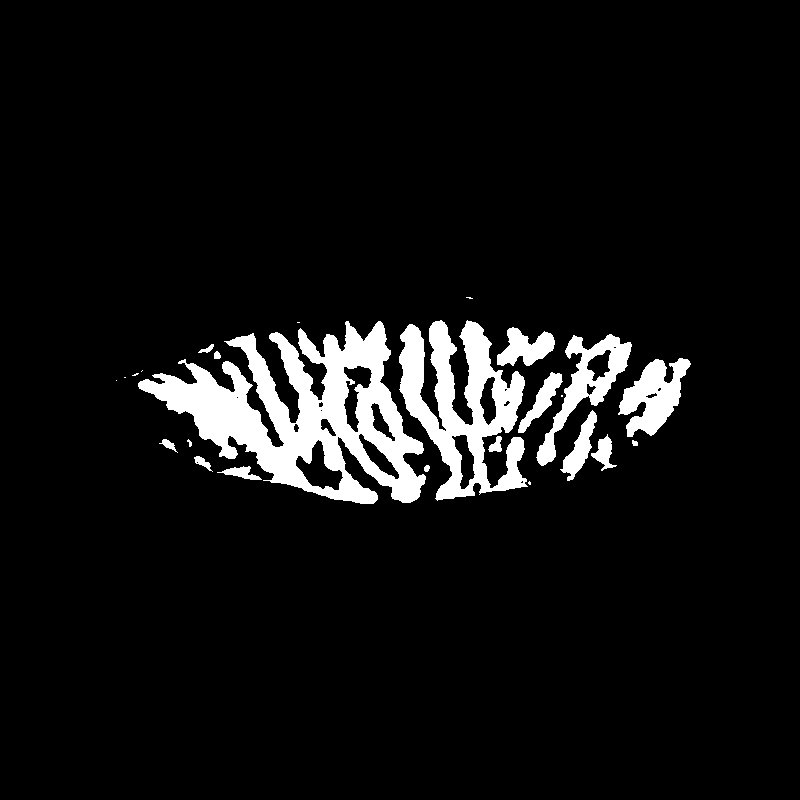

Supplement: S1 Raw images — (ZIP) [file pone.0270473.s008.zip › Intermediate/intermediate 22.jpg]

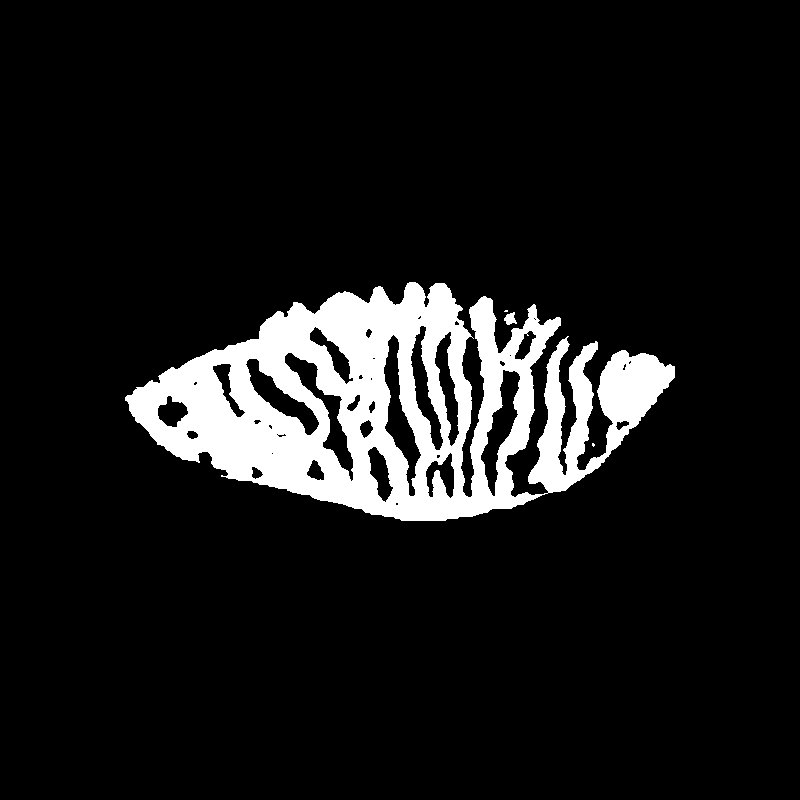

Supplement: S1 Raw images — (ZIP) [file pone.0270473.s008.zip › Intermediate/intermediate 23.jpg]

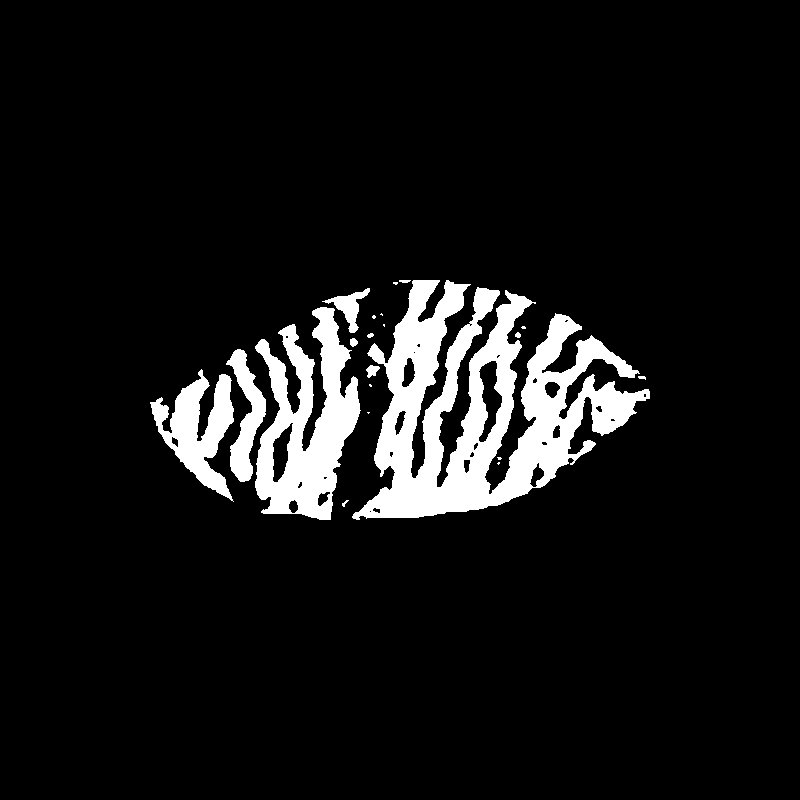

Supplement: S1 Raw images — (ZIP) [file pone.0270473.s008.zip › Intermediate/intermediate 24.jpg]

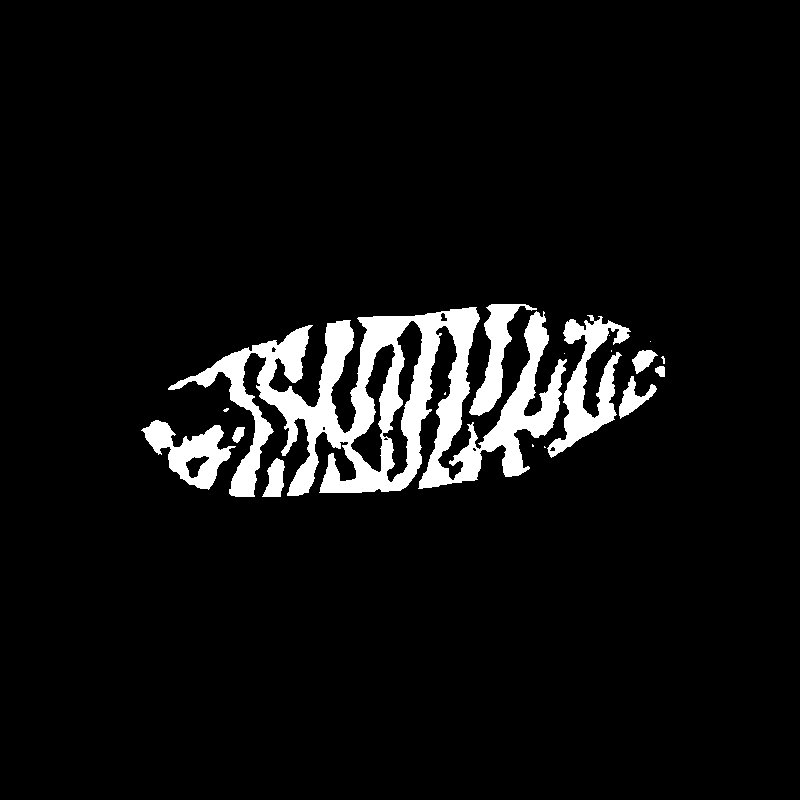

Supplement: S1 Raw images — (ZIP) [file pone.0270473.s008.zip › Intermediate/intermediate 25.jpg]

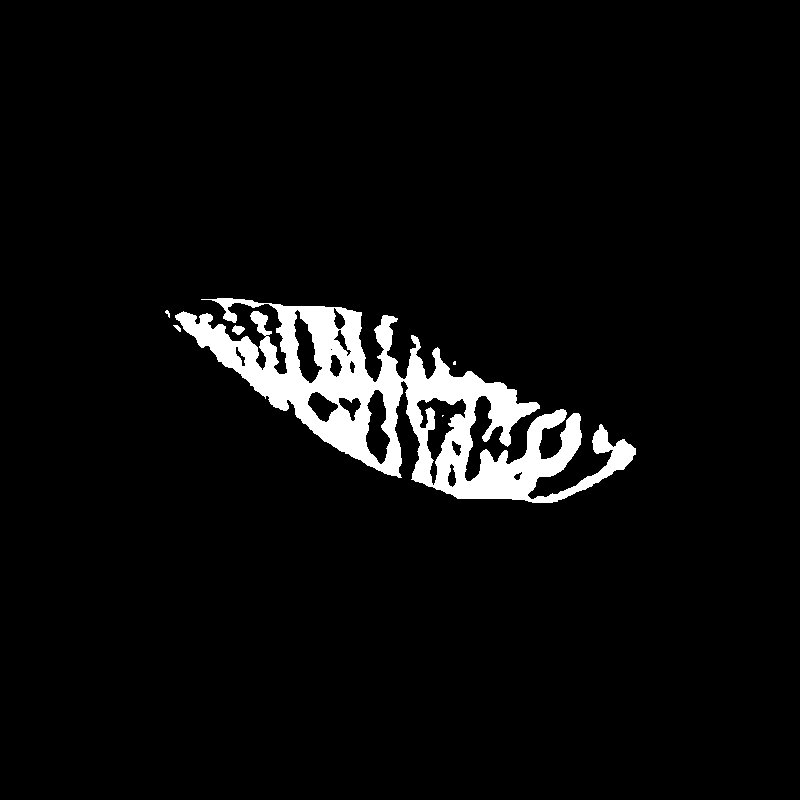

Supplement: S1 Raw images — (ZIP) [file pone.0270473.s008.zip › Intermediate/intermediate 26.jpg]

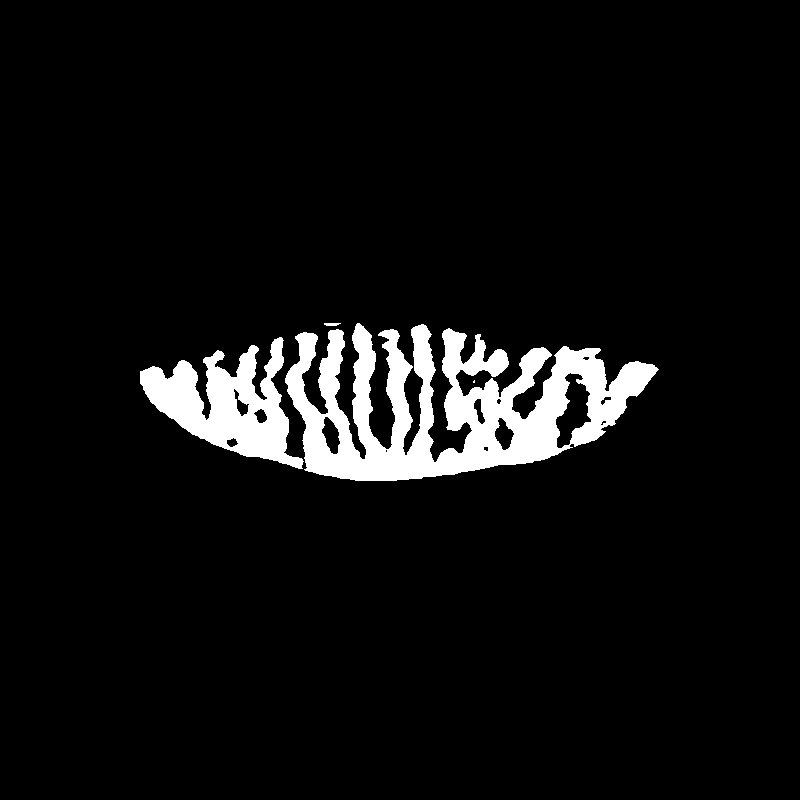

Supplement: S1 Raw images — (ZIP) [file pone.0270473.s008.zip › Intermediate/intermediate 27.jpg]

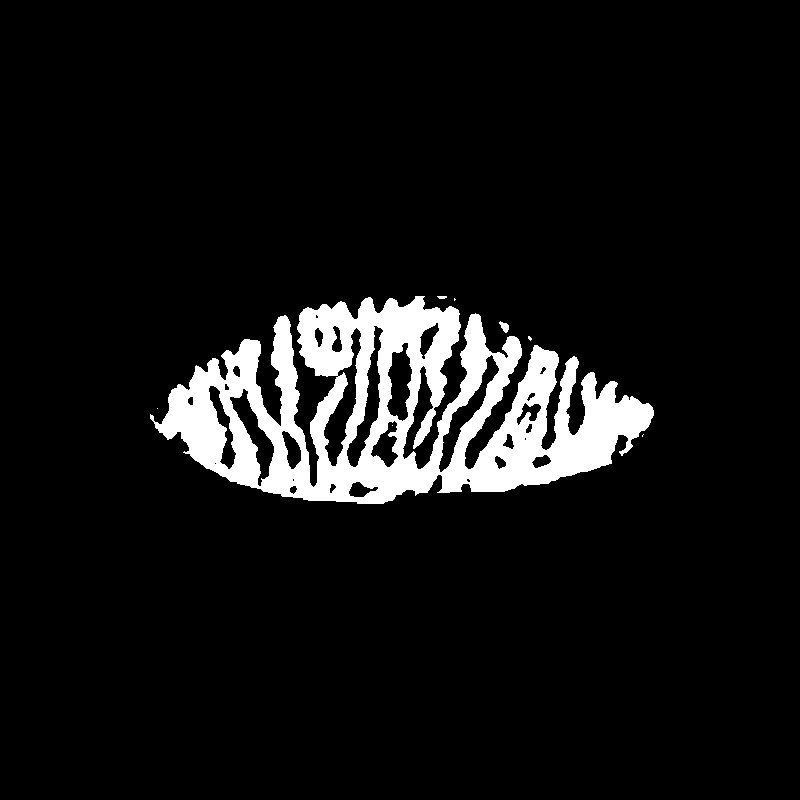

Supplement: S1 Raw images — (ZIP) [file pone.0270473.s008.zip › Intermediate/intermediate 28.jpg]

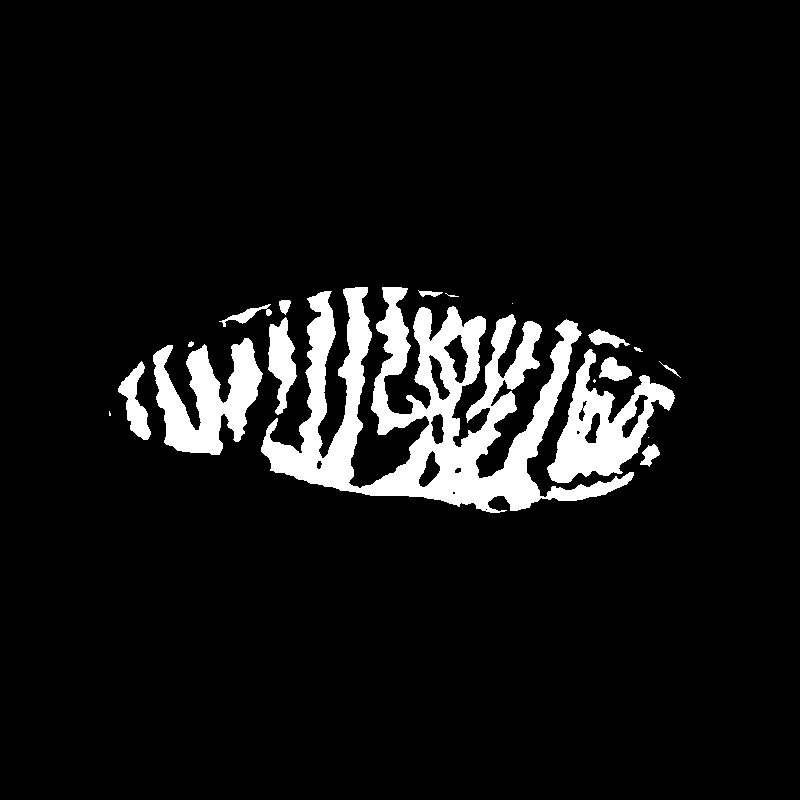

Supplement: S1 Raw images — (ZIP) [file pone.0270473.s008.zip › Intermediate/intermediate 29.jpg]

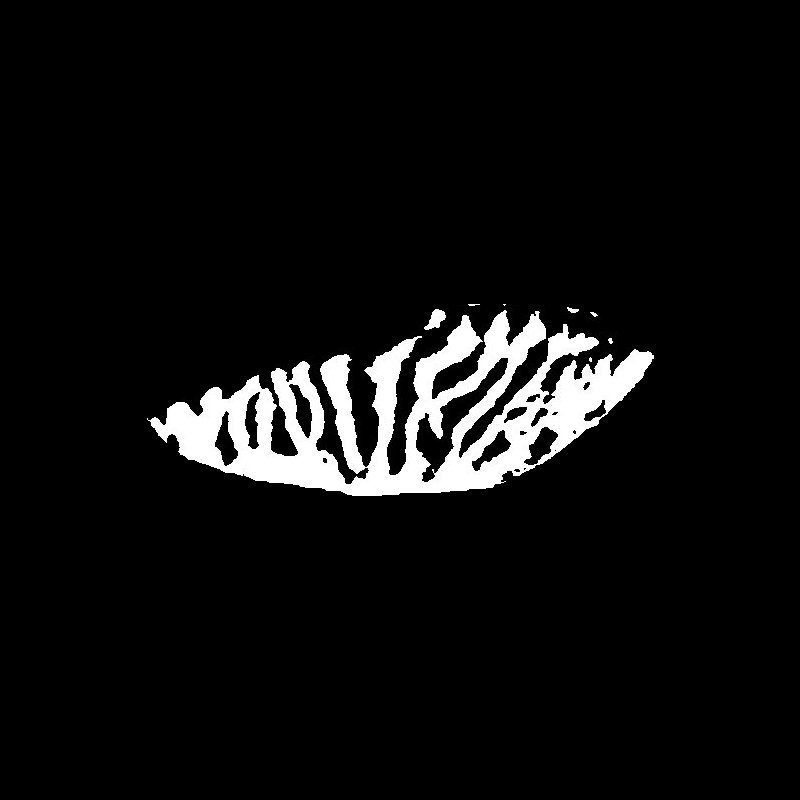

Supplement: S1 Raw images — (ZIP) [file pone.0270473.s008.zip › Intermediate/intermediate 3.jpg]

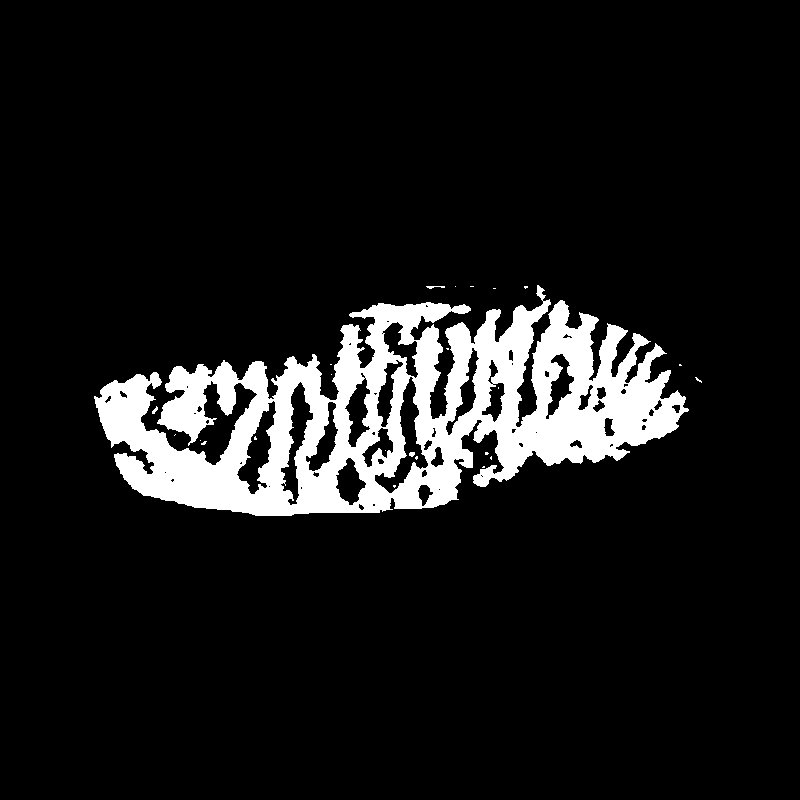

Supplement: S1 Raw images — (ZIP) [file pone.0270473.s008.zip › Intermediate/intermediate 30.jpg]

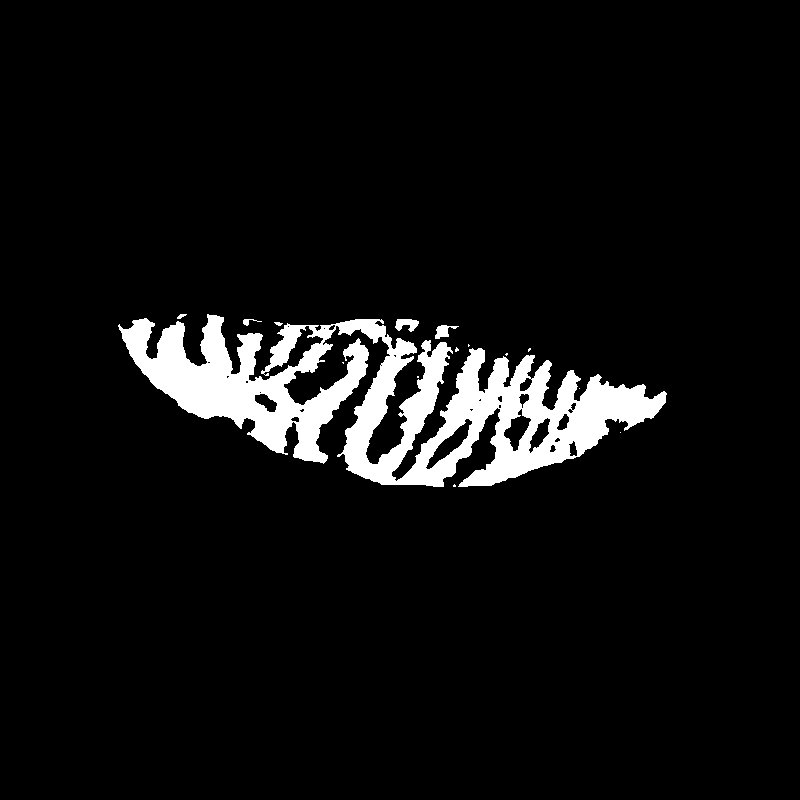

Supplement: S1 Raw images — (ZIP) [file pone.0270473.s008.zip › Intermediate/intermediate 31.jpg]

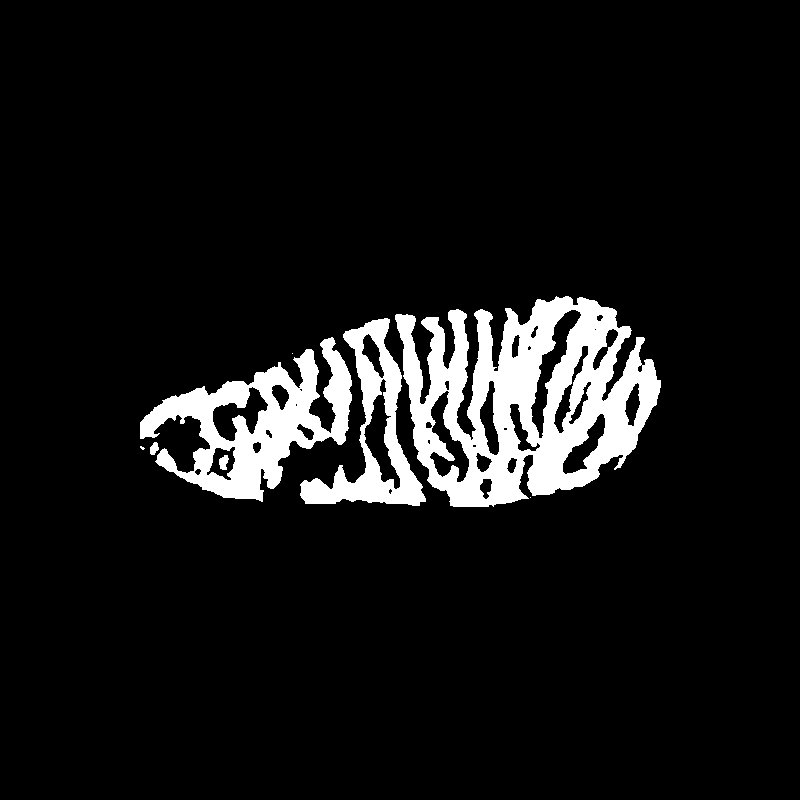

Supplement: S1 Raw images — (ZIP) [file pone.0270473.s008.zip › Intermediate/intermediate 32.jpg]

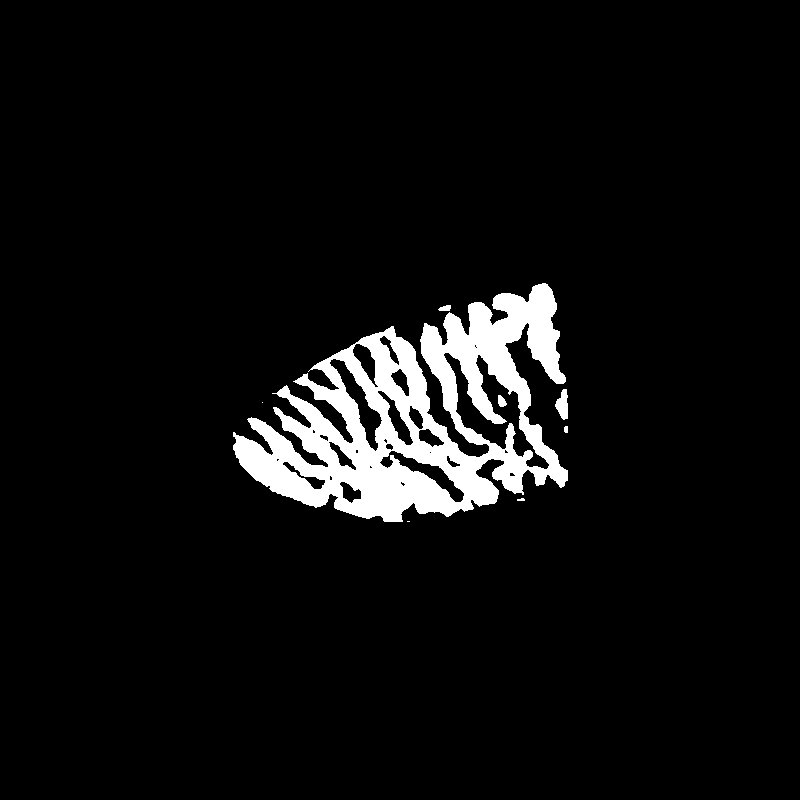

Supplement: S1 Raw images — (ZIP) [file pone.0270473.s008.zip › Intermediate/intermediate 33.jpg]

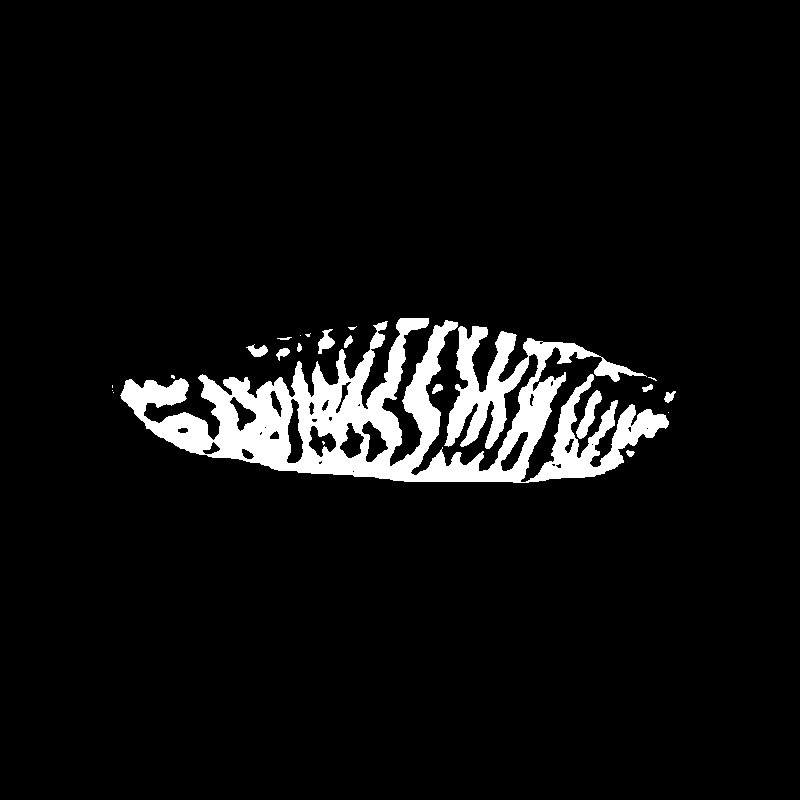

Supplement: S1 Raw images — (ZIP) [file pone.0270473.s008.zip › Intermediate/intermediate 34.jpg]

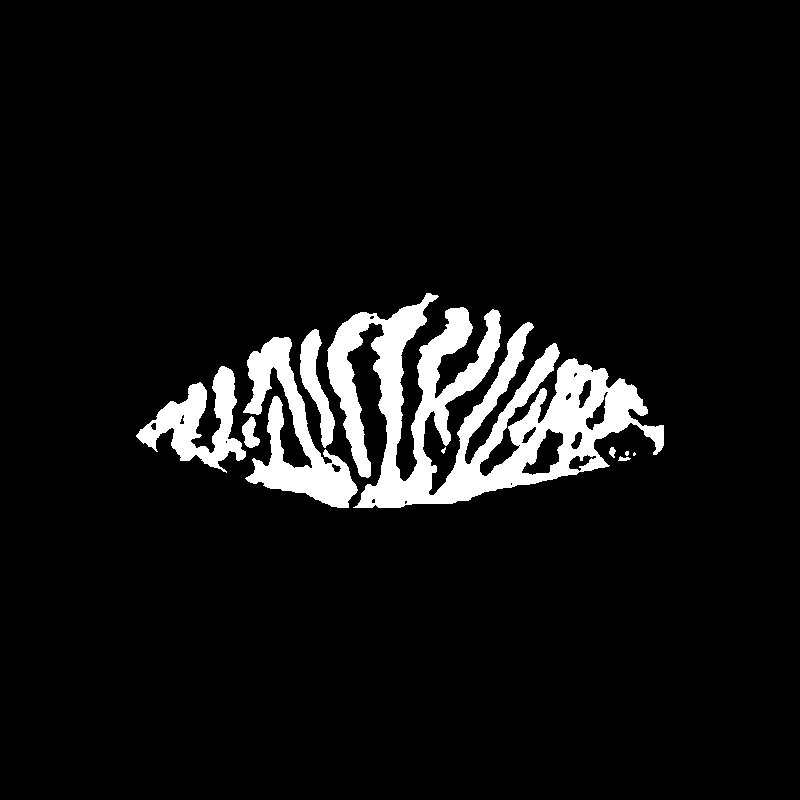

Supplement: S1 Raw images — (ZIP) [file pone.0270473.s008.zip › Intermediate/intermediate 35.jpg]

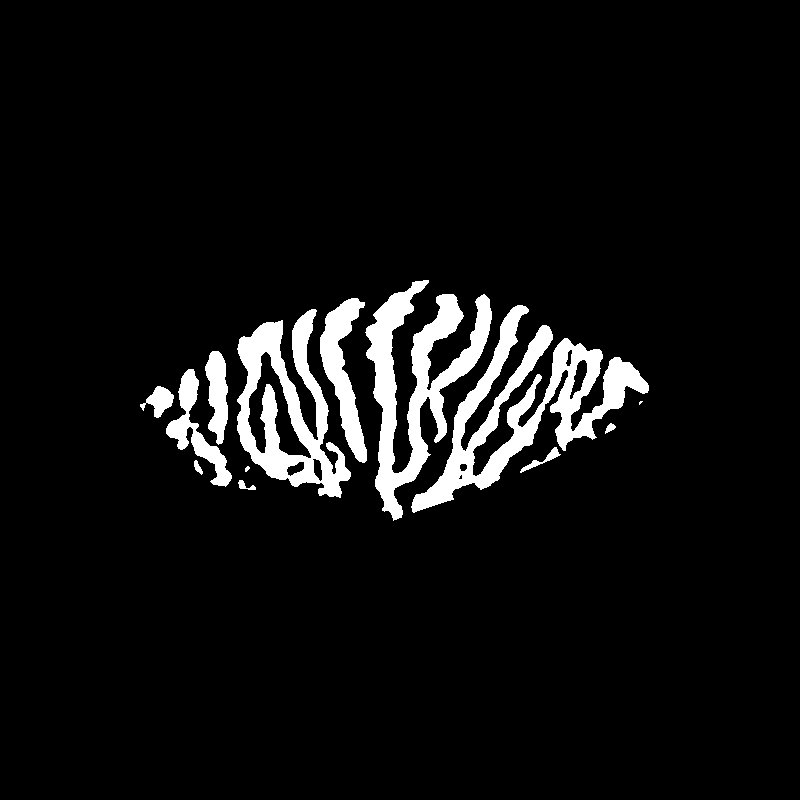

Supplement: S1 Raw images — (ZIP) [file pone.0270473.s008.zip › Intermediate/intermediate 36.jpg]

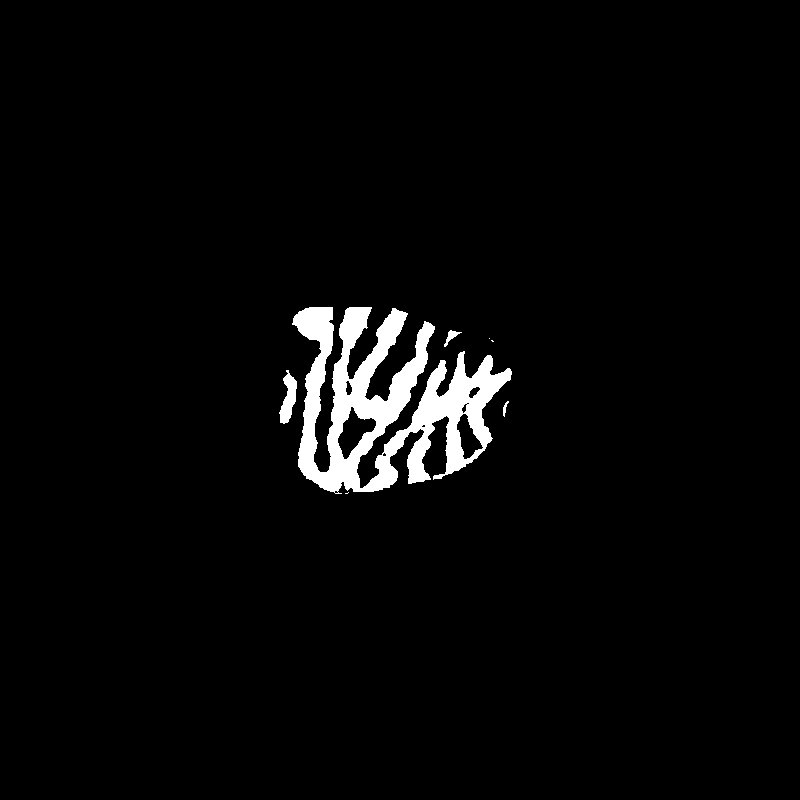

Supplement: S1 Raw images — (ZIP) [file pone.0270473.s008.zip › Intermediate/intermediate 37.jpg]

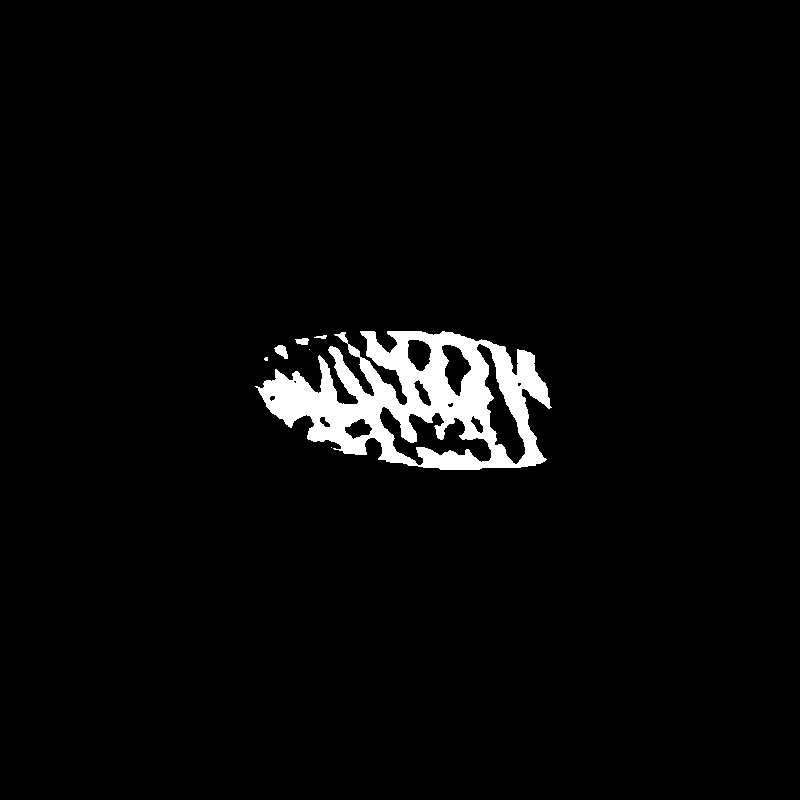

Supplement: S1 Raw images — (ZIP) [file pone.0270473.s008.zip › Intermediate/intermediate 38.jpg]

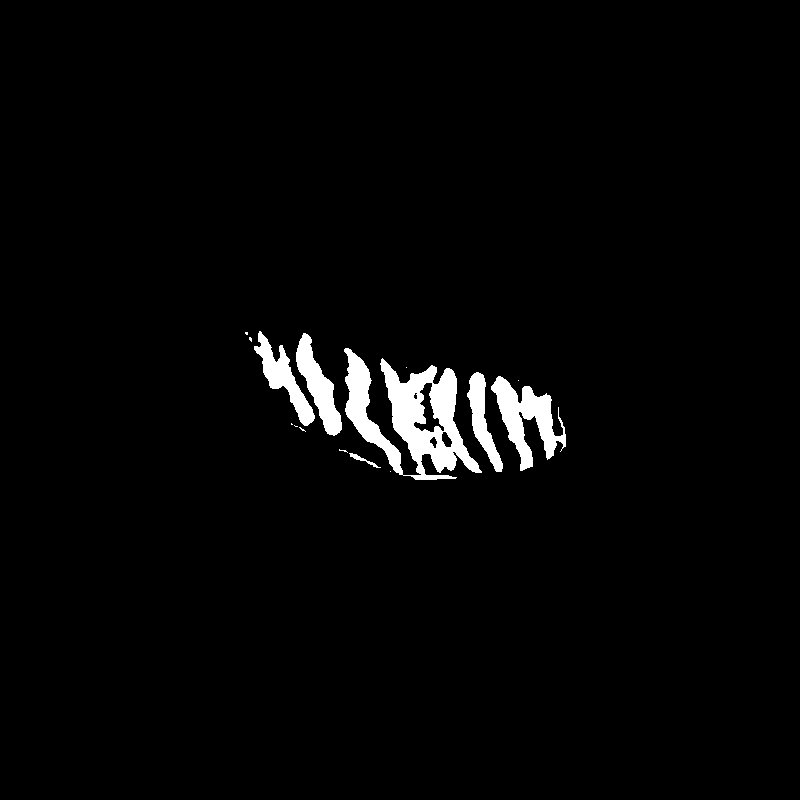

Supplement: S1 Raw images — (ZIP) [file pone.0270473.s008.zip › Intermediate/intermediate 39.jpg]

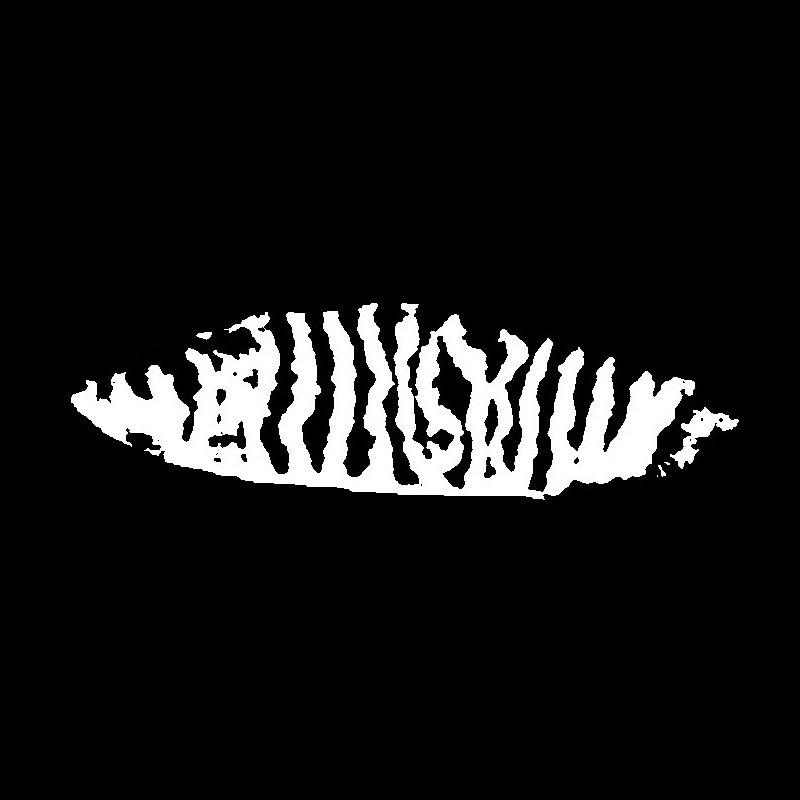

Supplement: S1 Raw images — (ZIP) [file pone.0270473.s008.zip › Intermediate/intermediate 4.jpg]

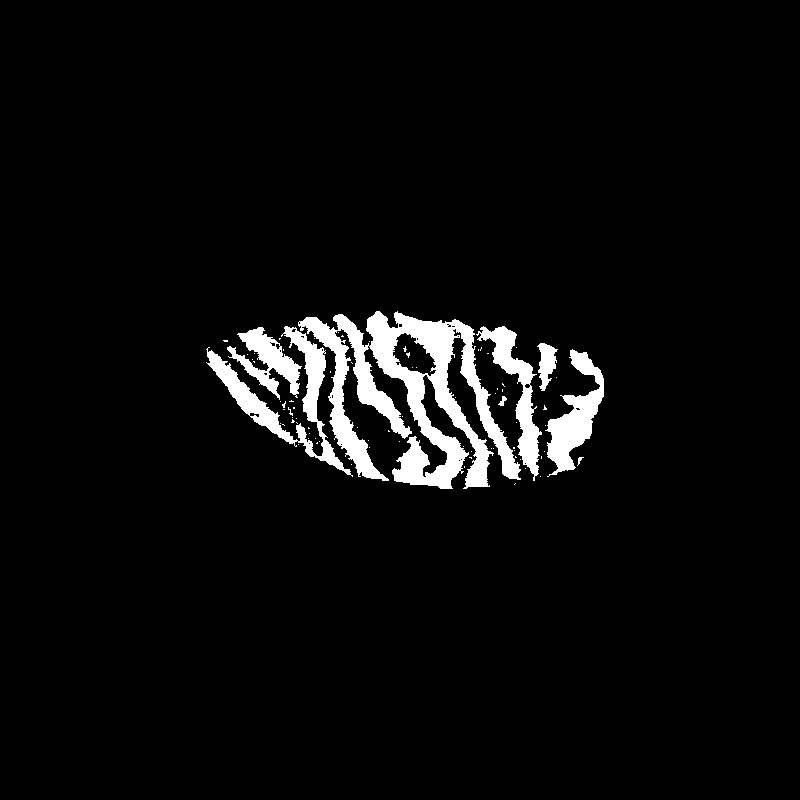

Supplement: S1 Raw images — (ZIP) [file pone.0270473.s008.zip › Intermediate/intermediate 40.jpg]

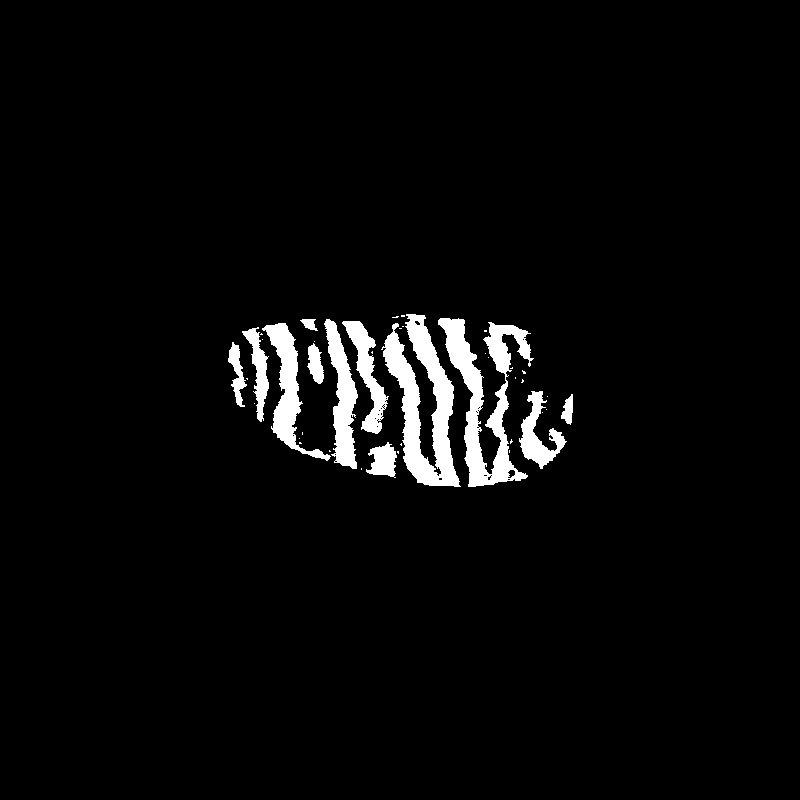

Supplement: S1 Raw images — (ZIP) [file pone.0270473.s008.zip › Intermediate/intermediate 41.jpg]

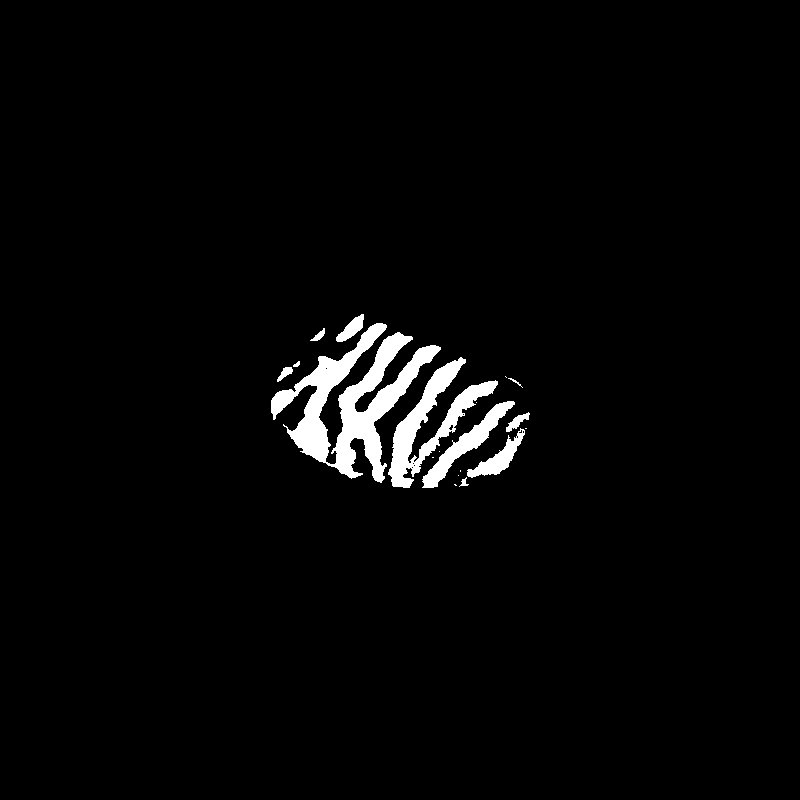

Supplement: S1 Raw images — (ZIP) [file pone.0270473.s008.zip › Intermediate/intermediate 42.jpg]

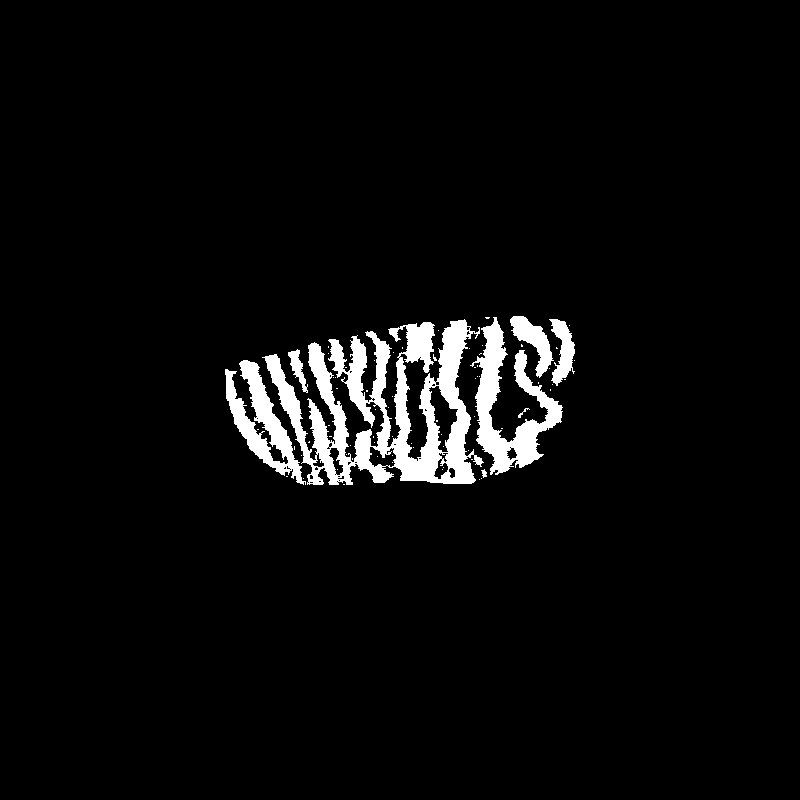

Supplement: S1 Raw images — (ZIP) [file pone.0270473.s008.zip › Intermediate/intermediate 43.jpg]

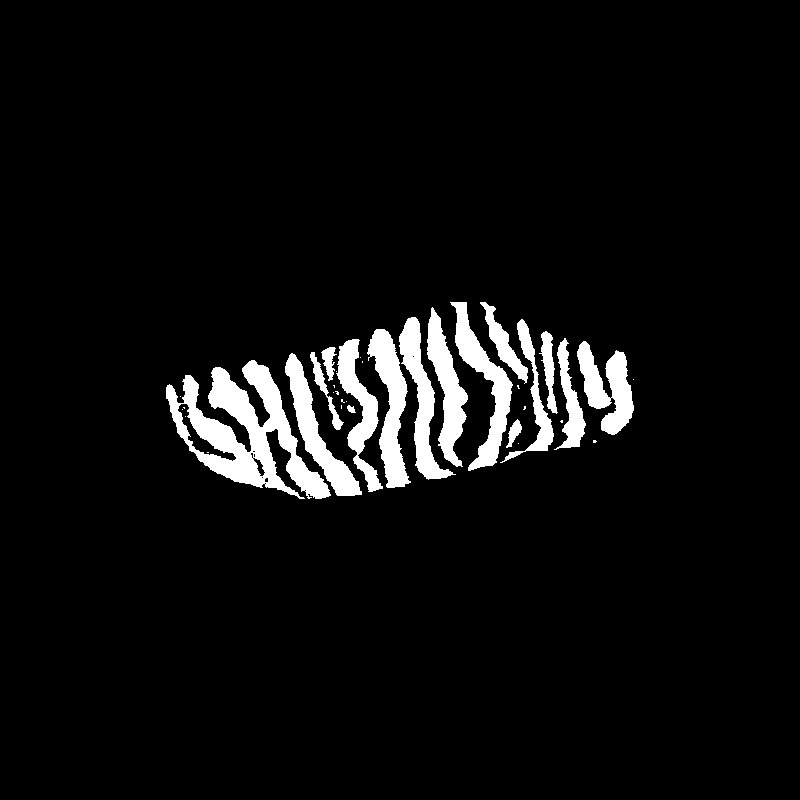

Supplement: S1 Raw images — (ZIP) [file pone.0270473.s008.zip › Intermediate/intermediate 44.jpg]

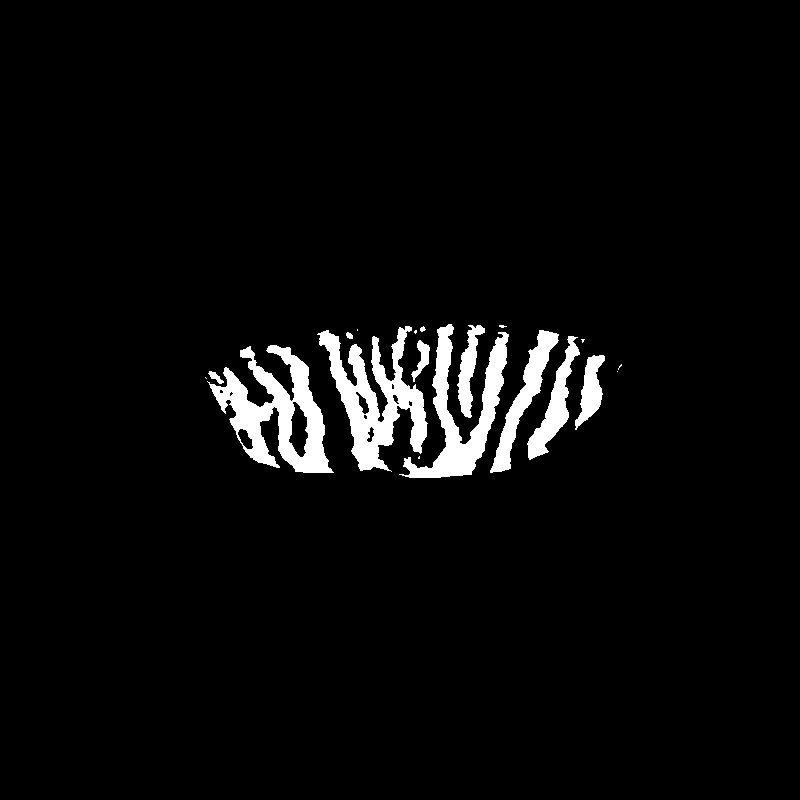

Supplement: S1 Raw images — (ZIP) [file pone.0270473.s008.zip › Intermediate/intermediate 45.jpg]

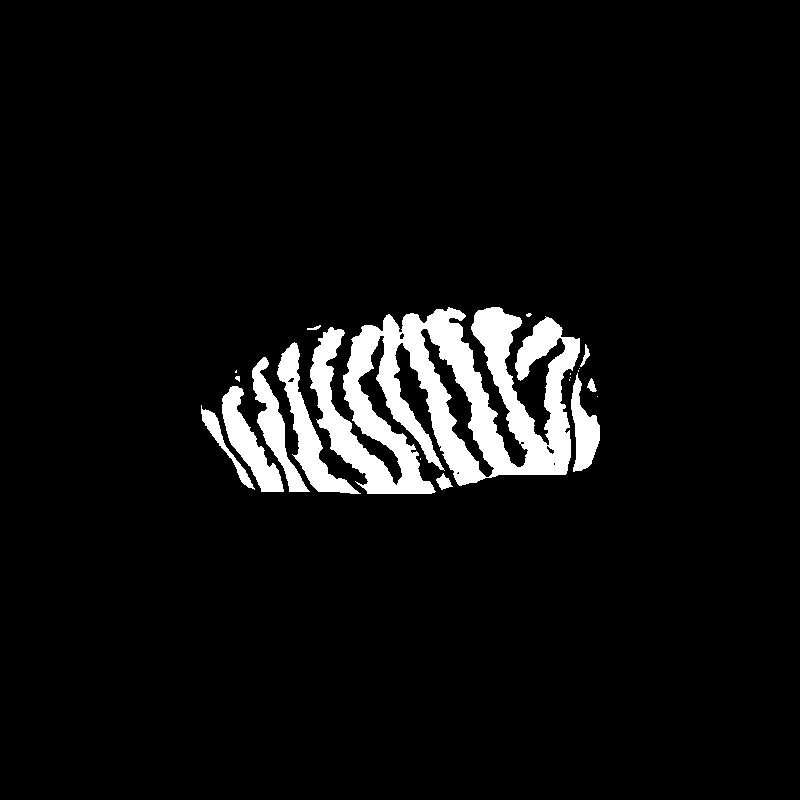

Supplement: S1 Raw images — (ZIP) [file pone.0270473.s008.zip › Intermediate/intermediate 46.jpg]

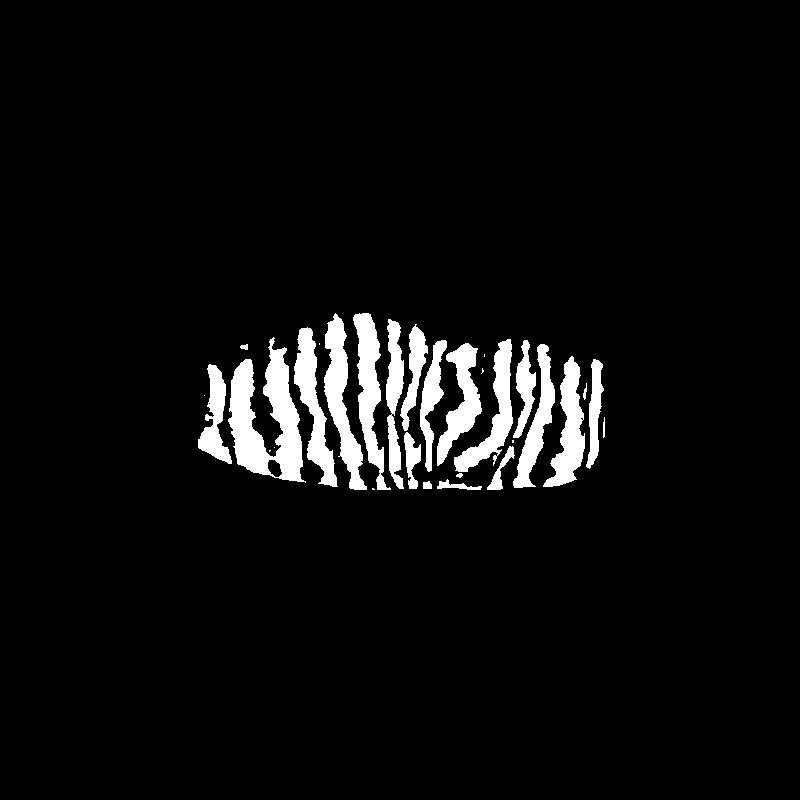

Supplement: S1 Raw images — (ZIP) [file pone.0270473.s008.zip › Intermediate/intermediate 47.jpg]

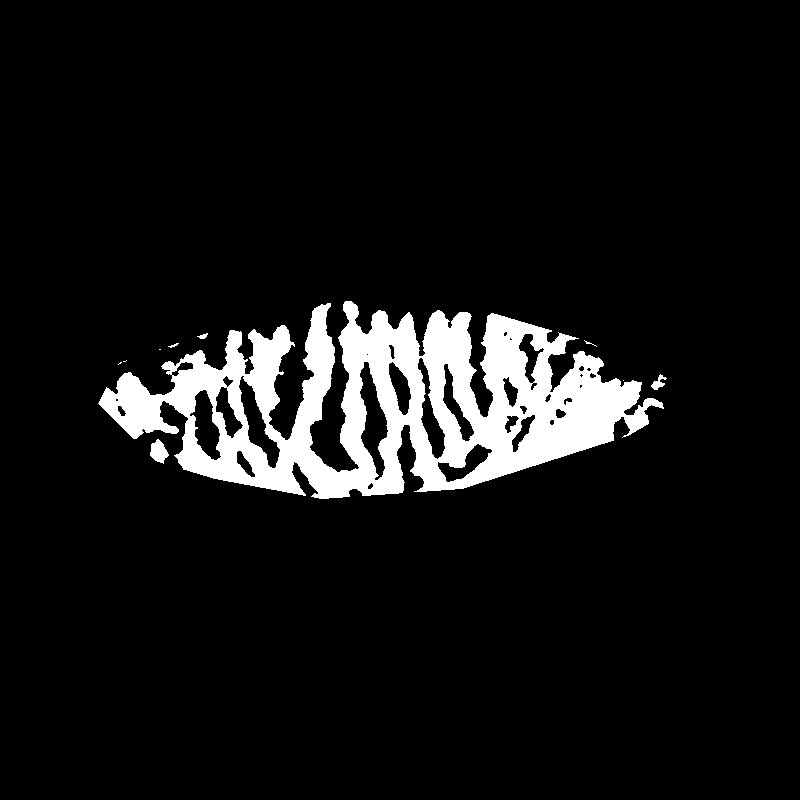

Supplement: S1 Raw images — (ZIP) [file pone.0270473.s008.zip › Intermediate/intermediate 48.jpg]

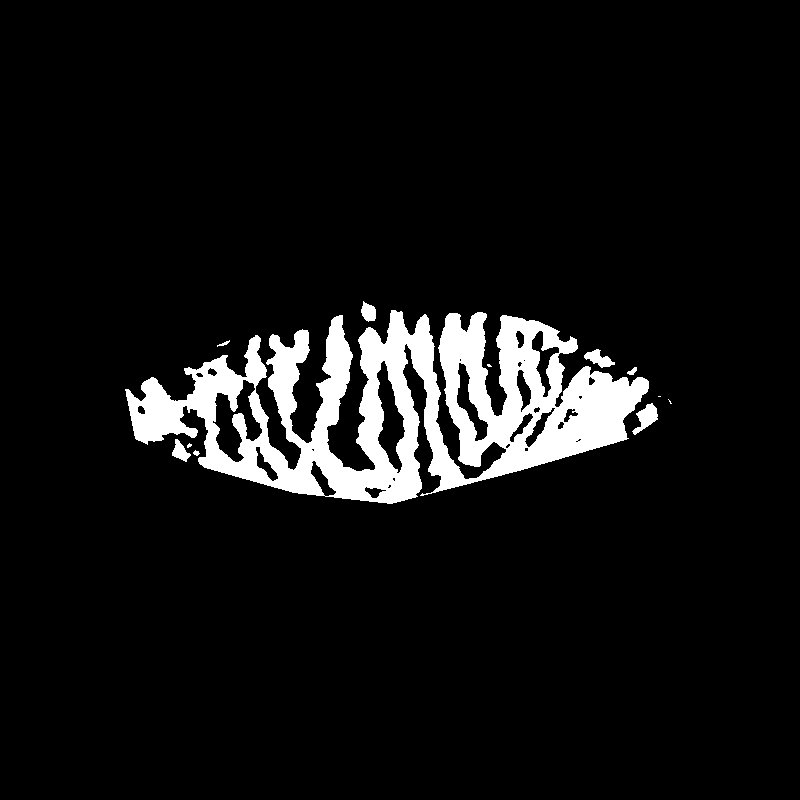

Supplement: S1 Raw images — (ZIP) [file pone.0270473.s008.zip › Intermediate/intermediate 49.jpg]

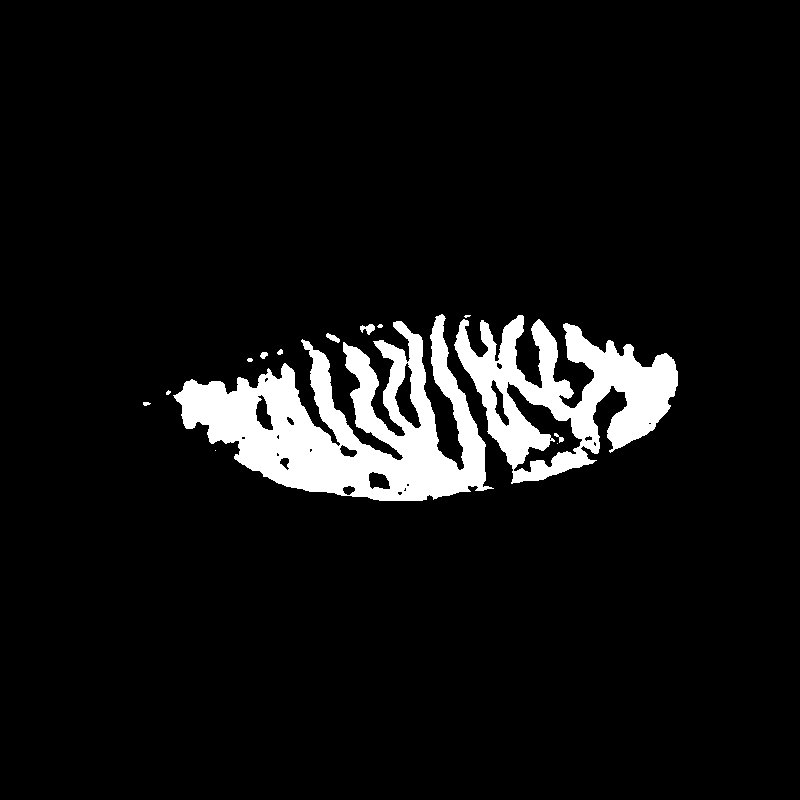

Supplement: S1 Raw images — (ZIP) [file pone.0270473.s008.zip › Intermediate/intermediate 5.jpg]

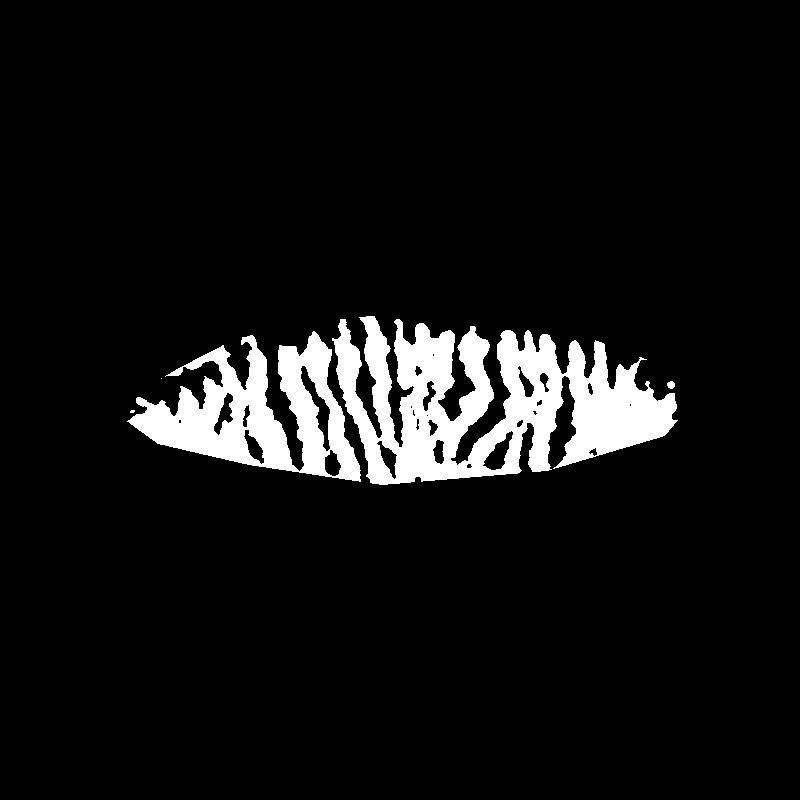

Supplement: S1 Raw images — (ZIP) [file pone.0270473.s008.zip › Intermediate/intermediate 50.jpg]

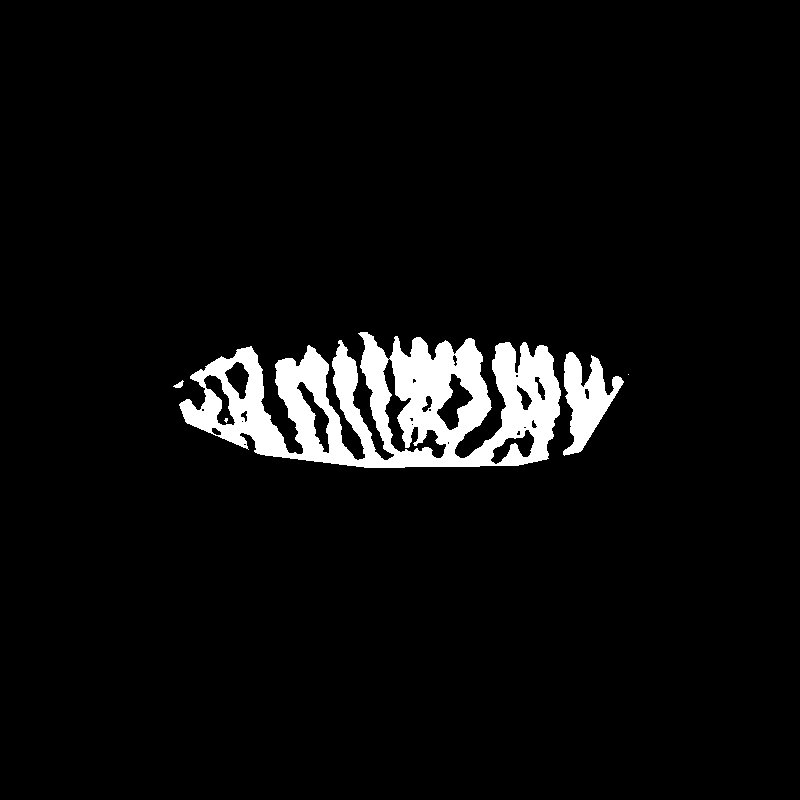

Supplement: S1 Raw images — (ZIP) [file pone.0270473.s008.zip › Intermediate/intermediate 51.jpg]

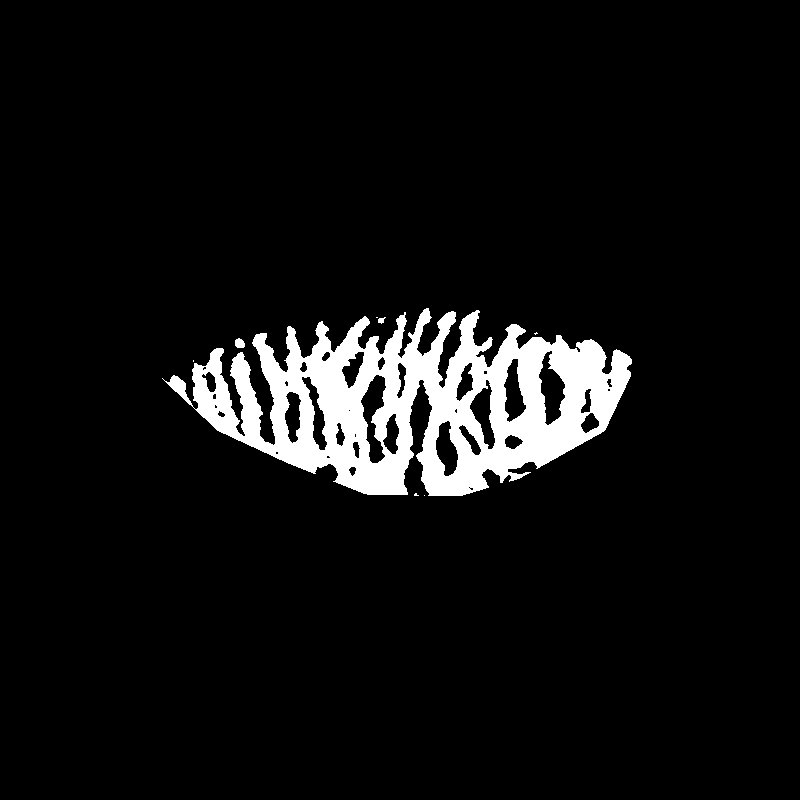

Supplement: S1 Raw images — (ZIP) [file pone.0270473.s008.zip › Intermediate/intermediate 52.jpg]

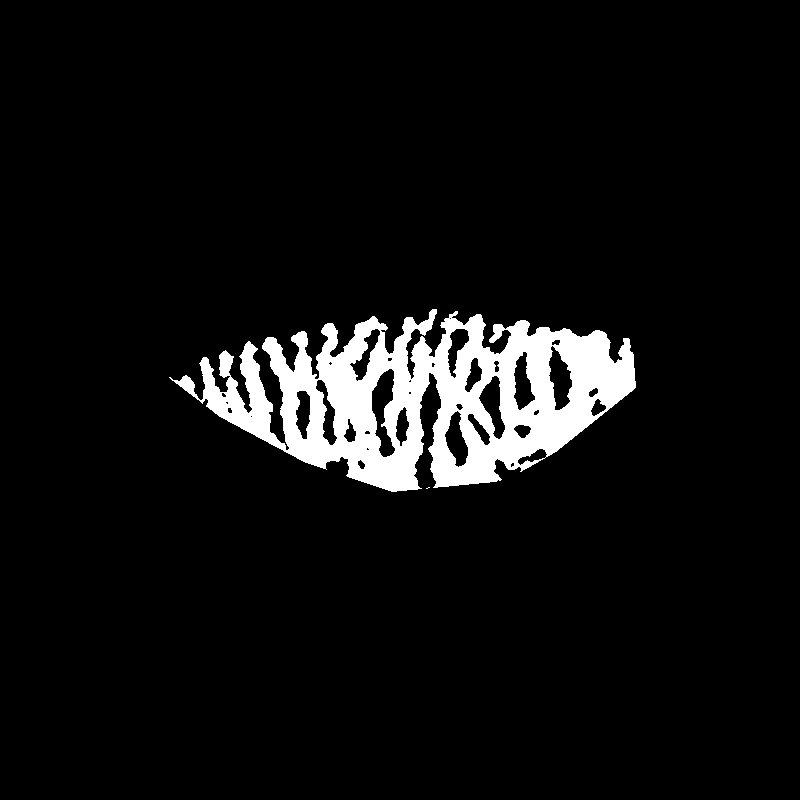

Supplement: S1 Raw images — (ZIP) [file pone.0270473.s008.zip › Intermediate/intermediate 53.jpg]

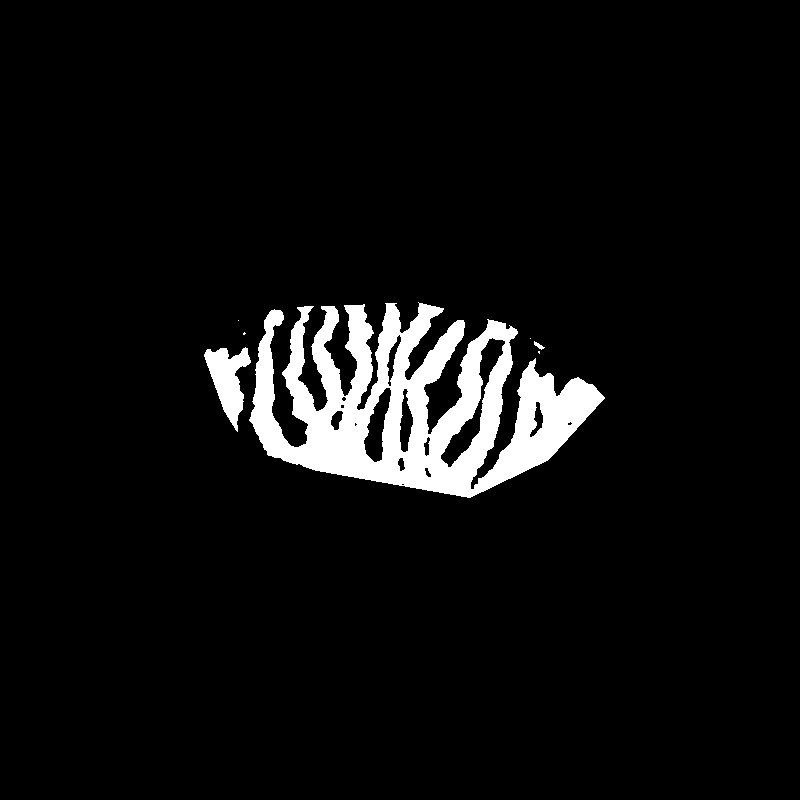

Supplement: S1 Raw images — (ZIP) [file pone.0270473.s008.zip › Intermediate/intermediate 54.jpg]

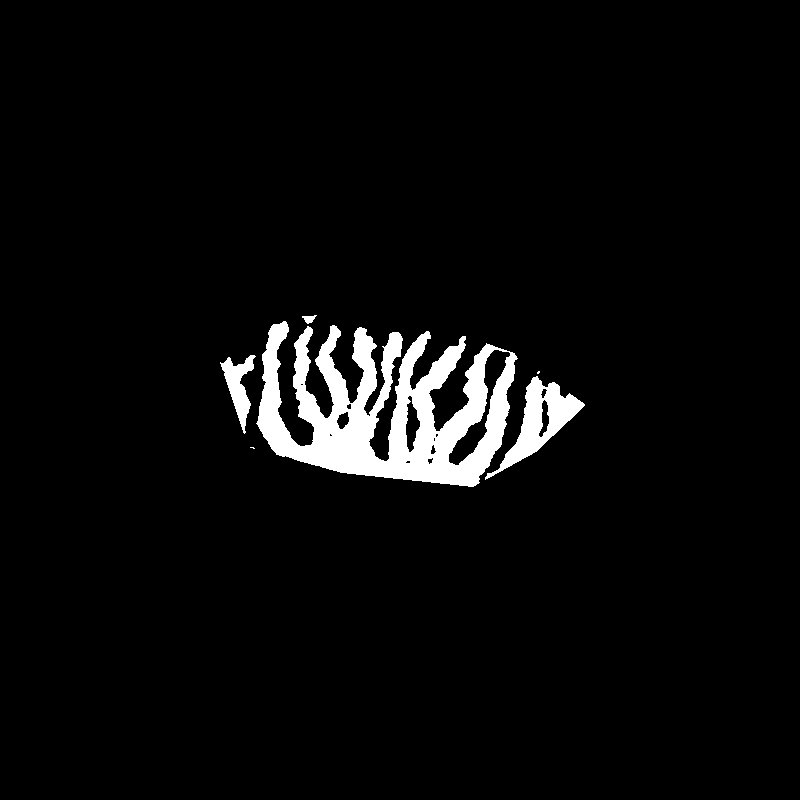

Supplement: S1 Raw images — (ZIP) [file pone.0270473.s008.zip › Intermediate/intermediate 55.jpg]

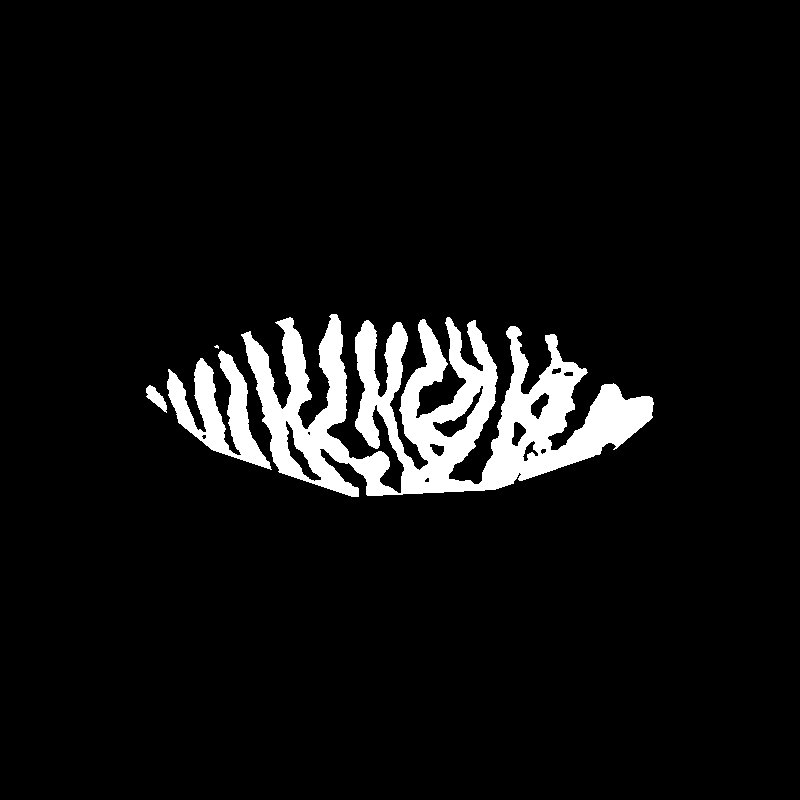

Supplement: S1 Raw images — (ZIP) [file pone.0270473.s008.zip › Intermediate/intermediate 56.jpg]

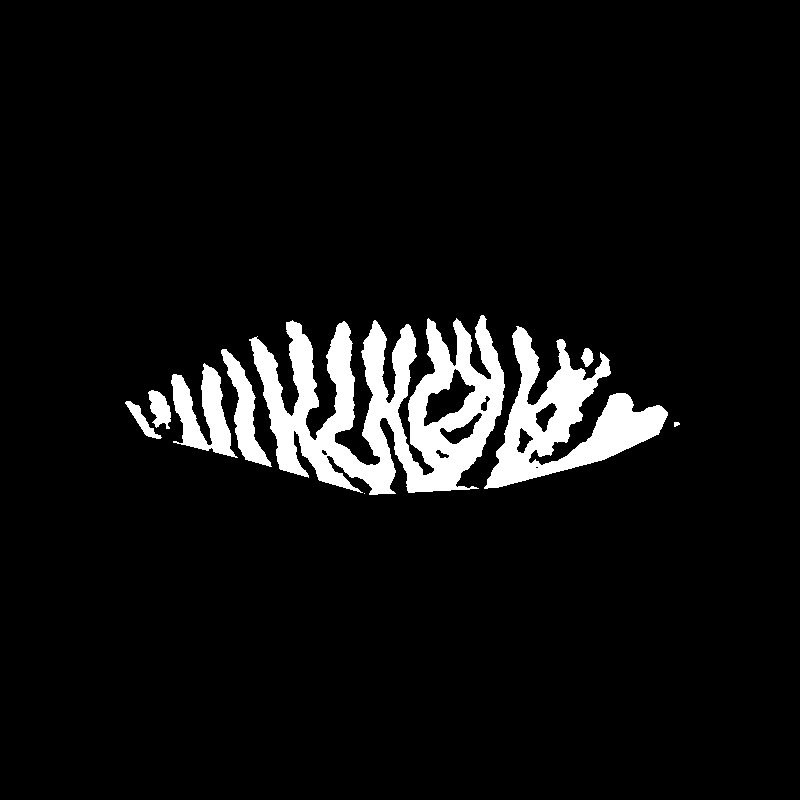

Supplement: S1 Raw images — (ZIP) [file pone.0270473.s008.zip › Intermediate/intermediate 57.jpg]

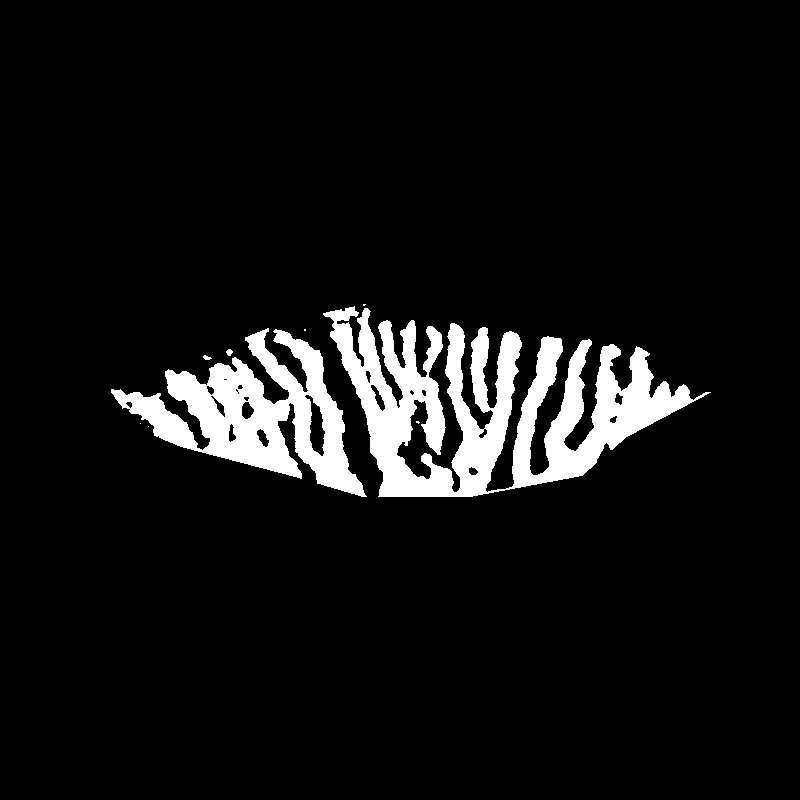

Supplement: S1 Raw images — (ZIP) [file pone.0270473.s008.zip › Intermediate/intermediate 58.jpg]

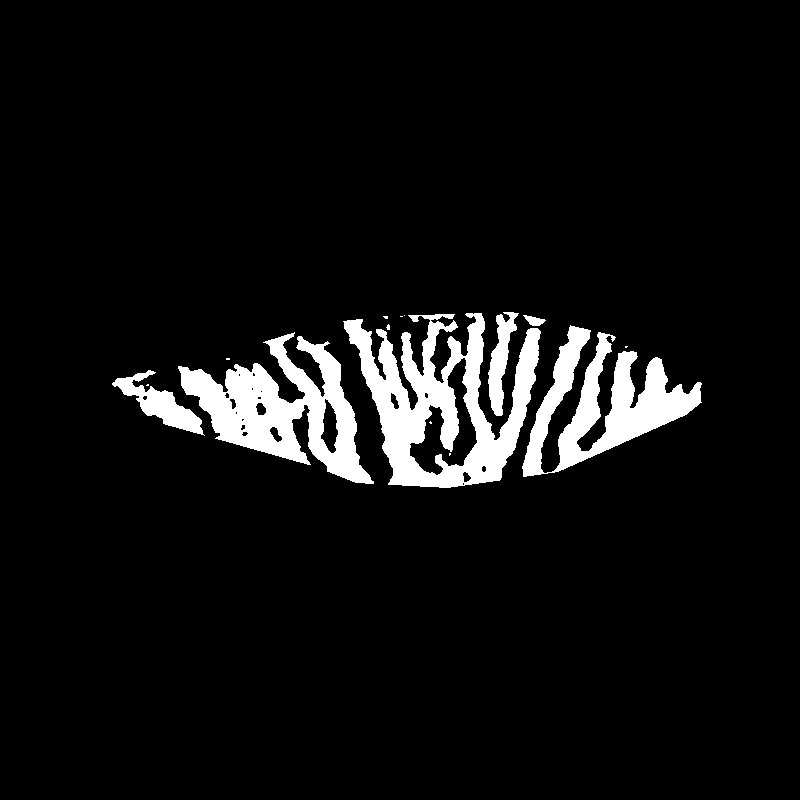

Supplement: S1 Raw images — (ZIP) [file pone.0270473.s008.zip › Intermediate/intermediate 59.jpg]

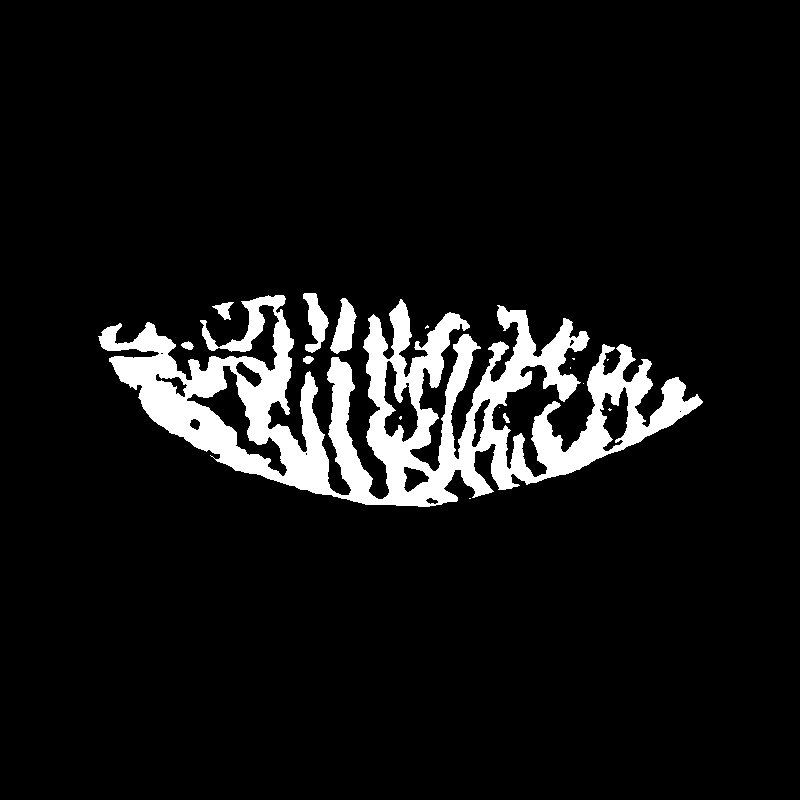

Supplement: S1 Raw images — (ZIP) [file pone.0270473.s008.zip › Intermediate/intermediate 6.jpg]

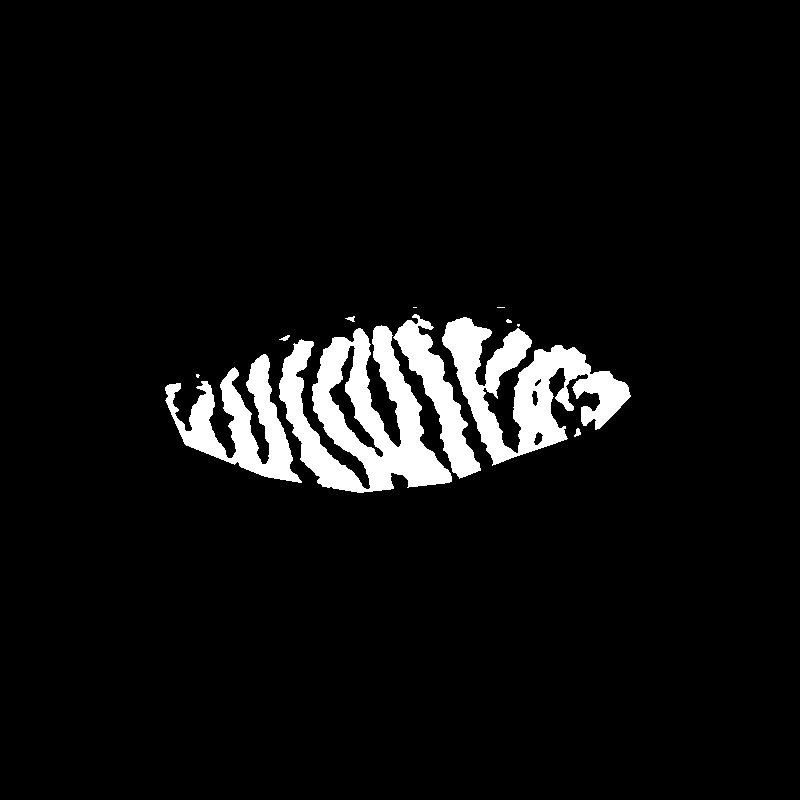

Supplement: S1 Raw images — (ZIP) [file pone.0270473.s008.zip › Intermediate/intermediate 60.jpg]

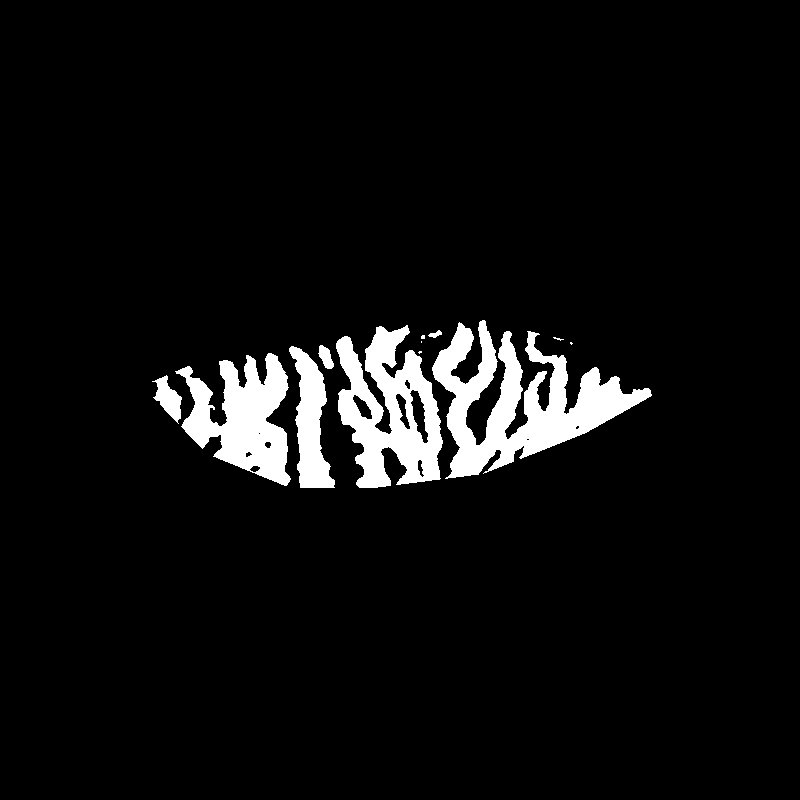

Supplement: S1 Raw images — (ZIP) [file pone.0270473.s008.zip › Intermediate/intermediate 61.jpg]

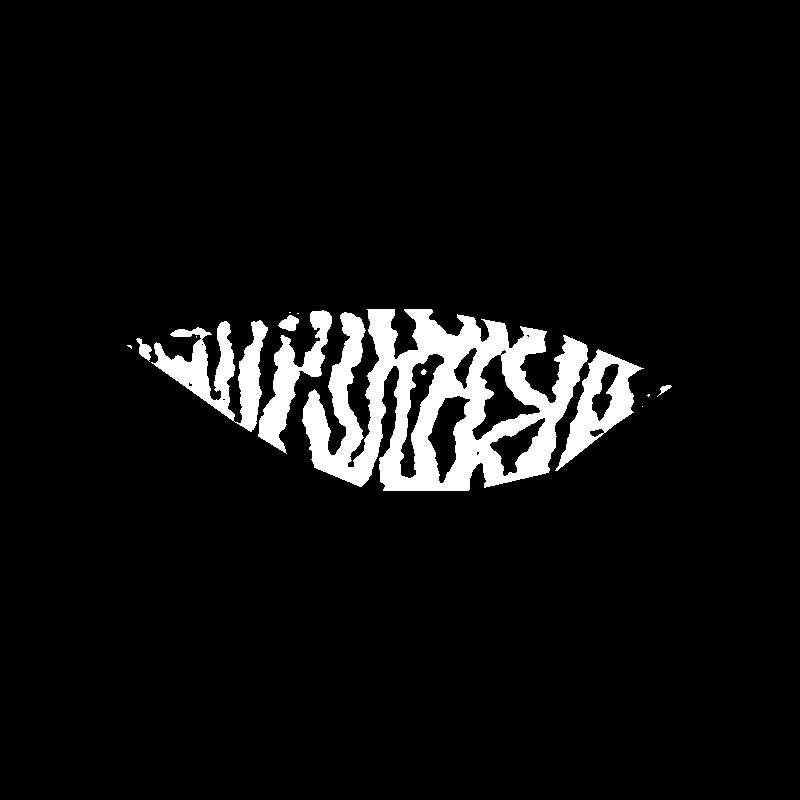

Supplement: S1 Raw images — (ZIP) [file pone.0270473.s008.zip › Intermediate/intermediate 62.jpg]

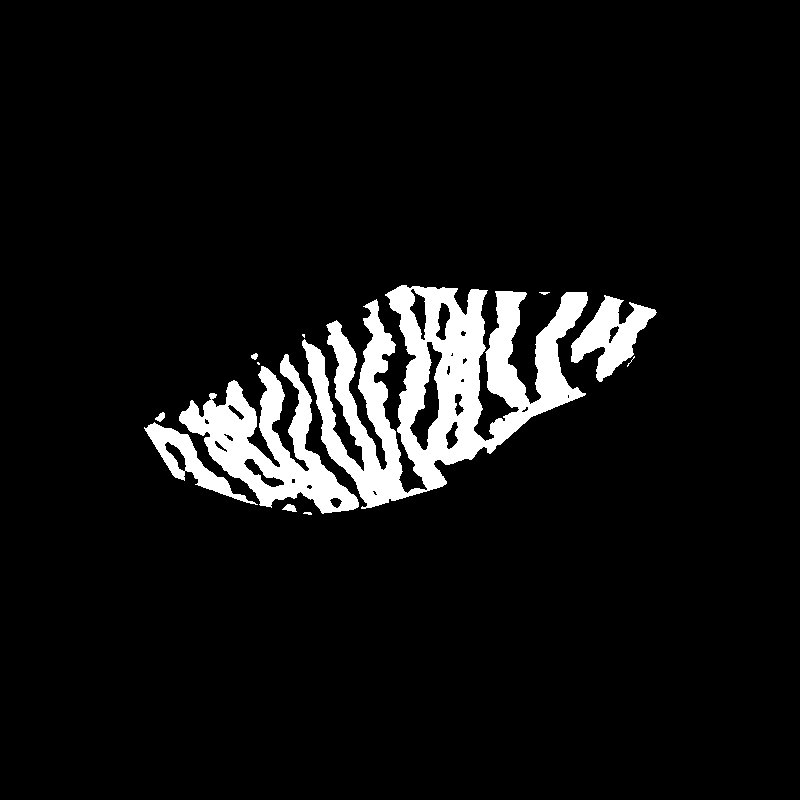

Supplement: S1 Raw images — (ZIP) [file pone.0270473.s008.zip › Intermediate/intermediate 63.jpg]

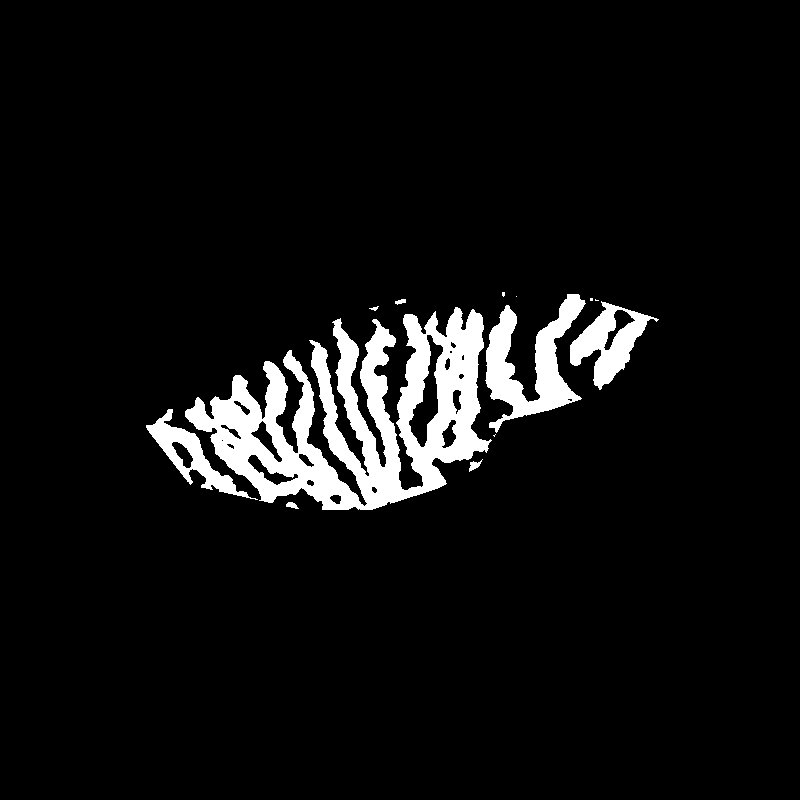

Supplement: S1 Raw images — (ZIP) [file pone.0270473.s008.zip › Intermediate/intermediate 64.jpg]

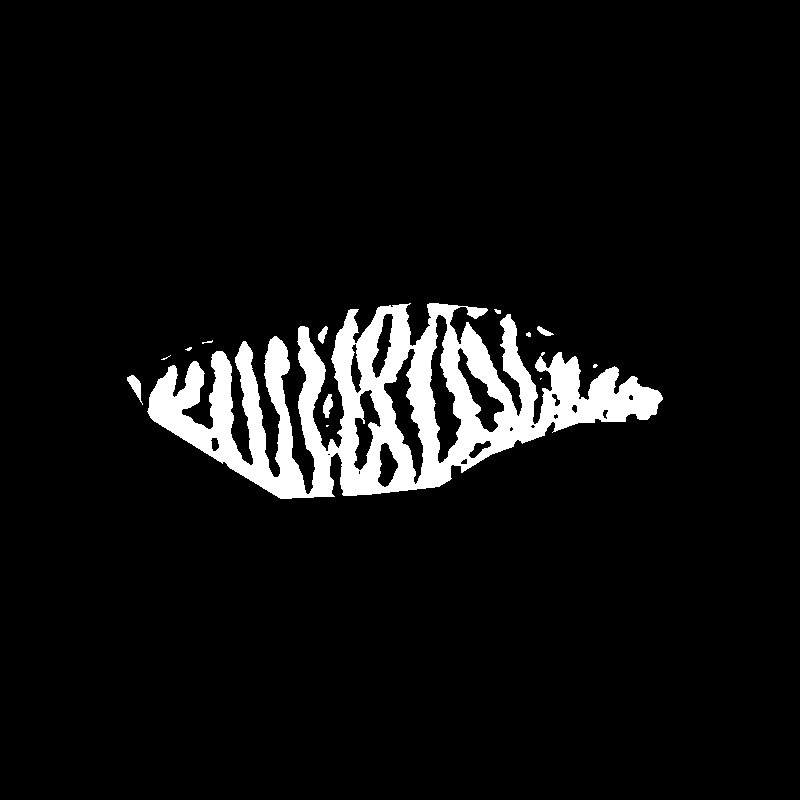

Supplement: S1 Raw images — (ZIP) [file pone.0270473.s008.zip › Intermediate/intermediate 65.jpg]

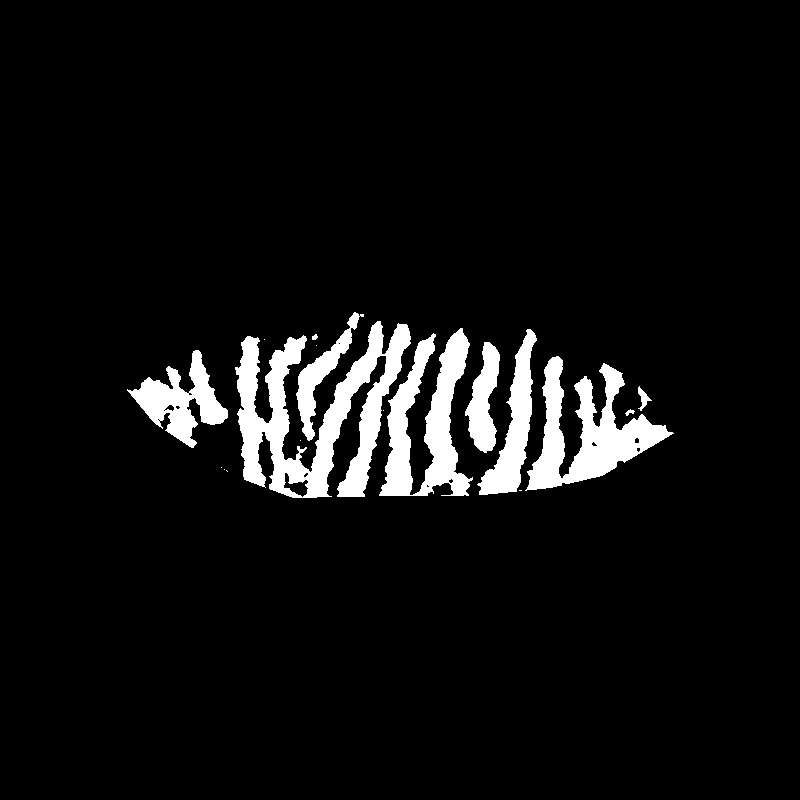

Supplement: S1 Raw images — (ZIP) [file pone.0270473.s008.zip › Intermediate/intermediate 66.jpg]

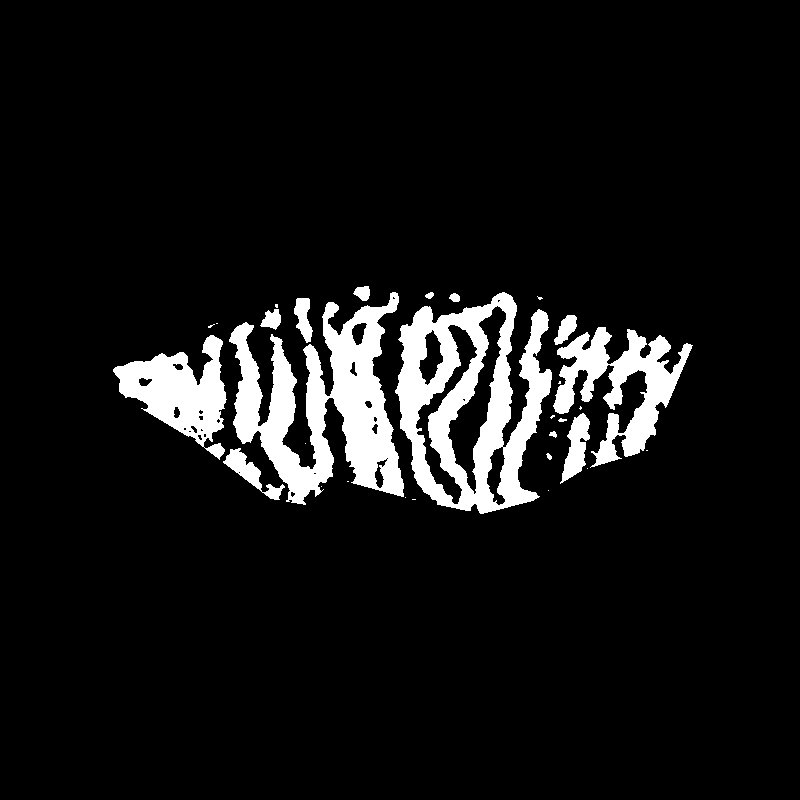

Supplement: S1 Raw images — (ZIP) [file pone.0270473.s008.zip › Intermediate/intermediate 67.jpg]

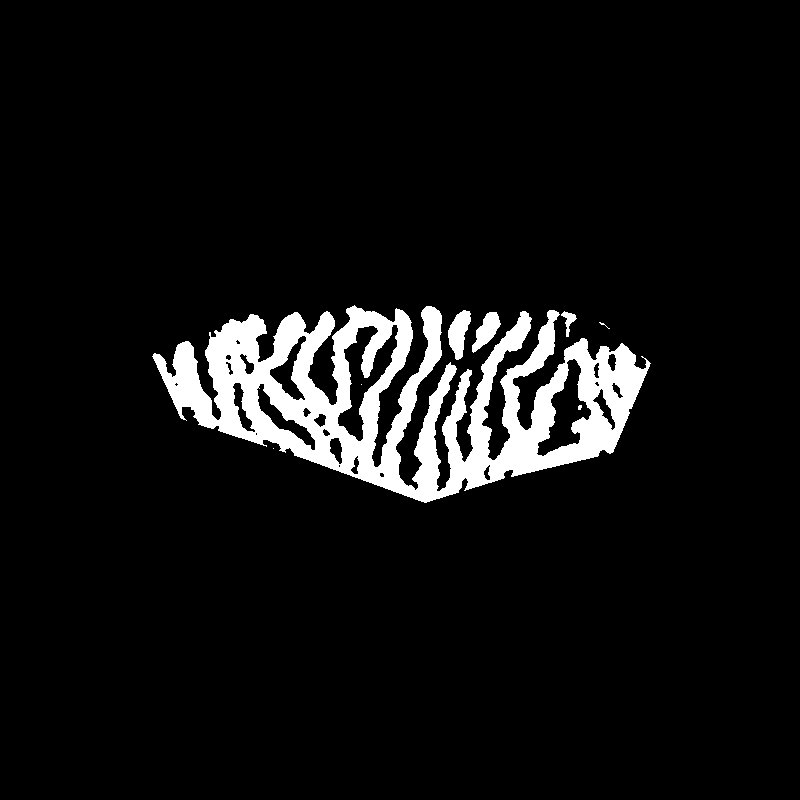

Supplement: S1 Raw images — (ZIP) [file pone.0270473.s008.zip › Intermediate/intermediate 68.jpg]

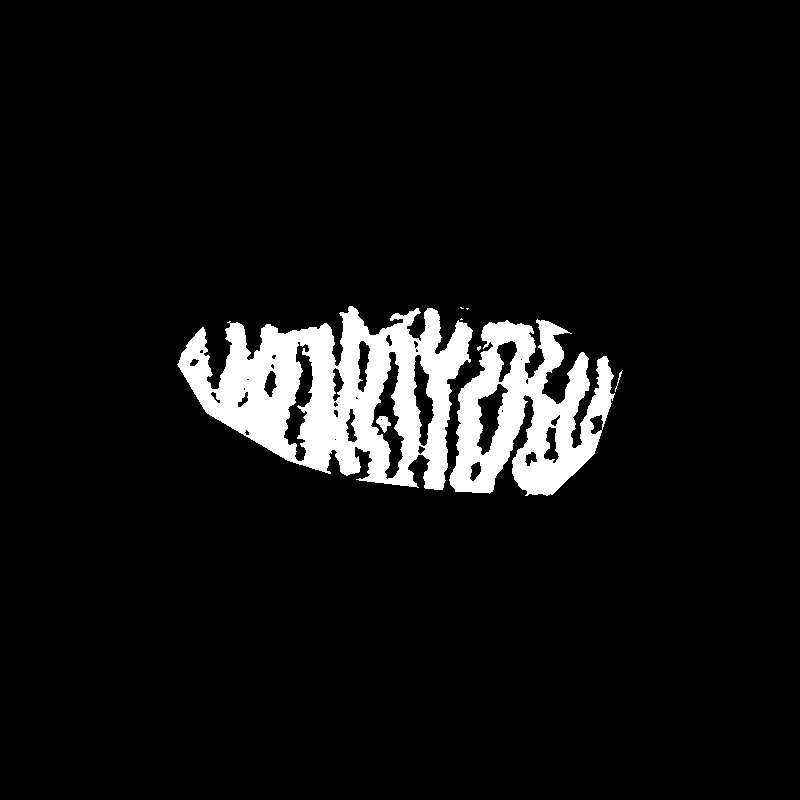

Supplement: S1 Raw images — (ZIP) [file pone.0270473.s008.zip › Intermediate/intermediate 69.jpg]

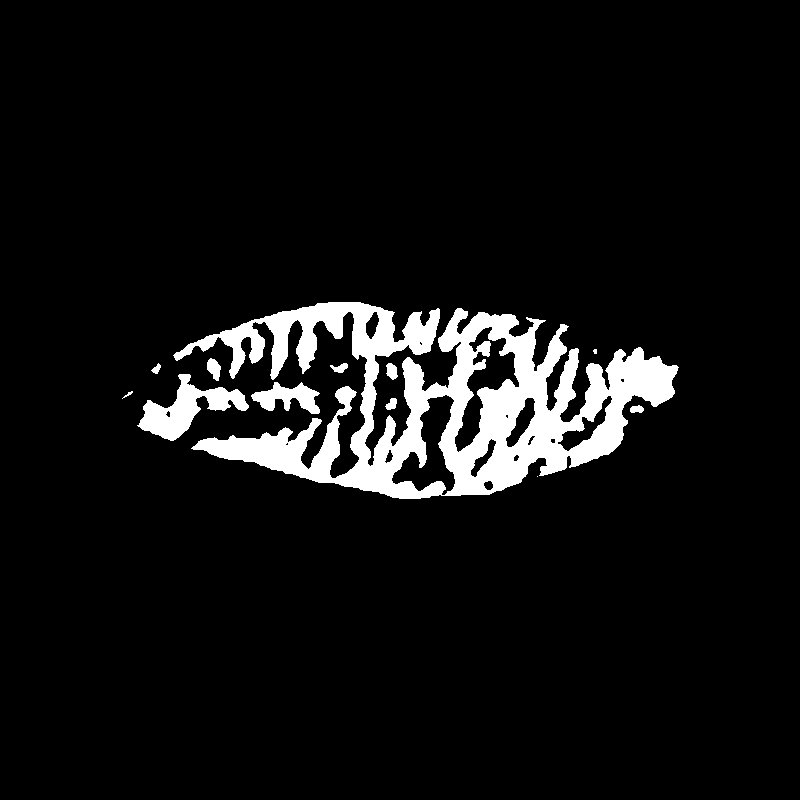

Supplement: S1 Raw images — (ZIP) [file pone.0270473.s008.zip › Intermediate/intermediate 7.jpg]

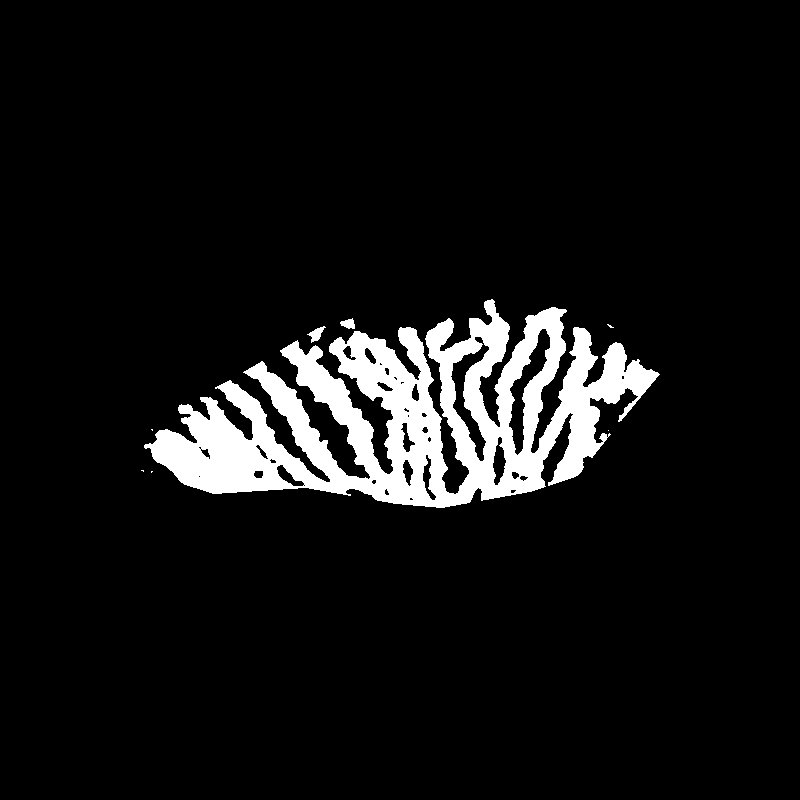

Supplement: S1 Raw images — (ZIP) [file pone.0270473.s008.zip › Intermediate/intermediate 70.jpg]

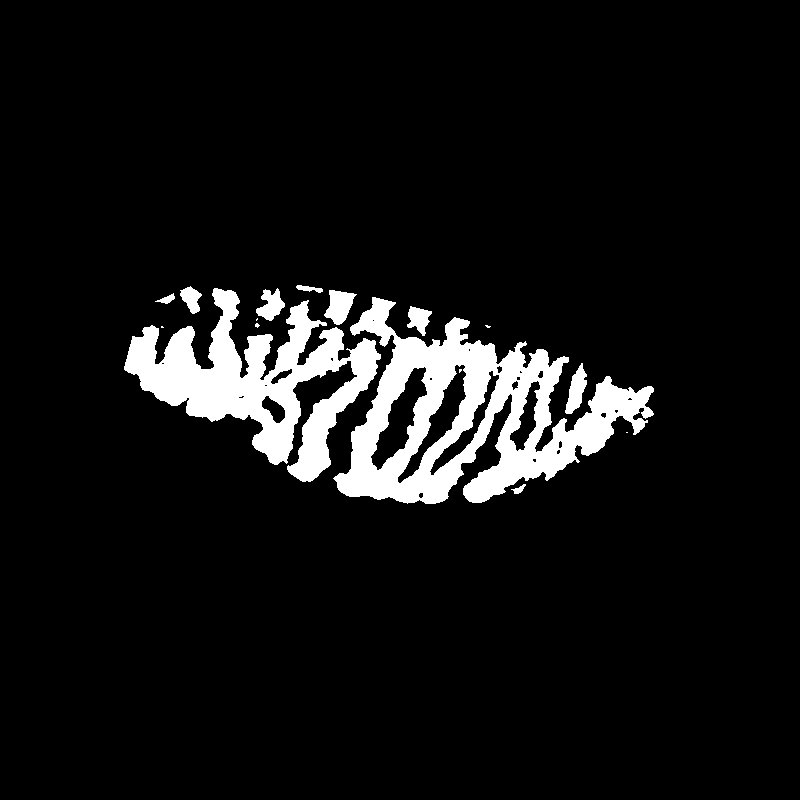

Supplement: S1 Raw images — (ZIP) [file pone.0270473.s008.zip › Intermediate/intermediate 71.jpg]

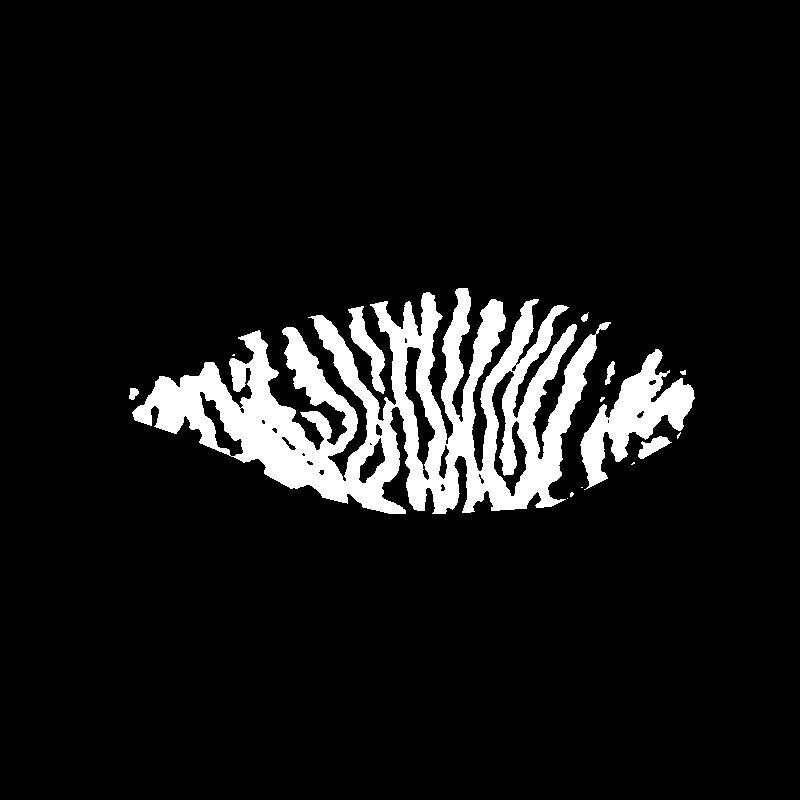

Supplement: S1 Raw images — (ZIP) [file pone.0270473.s008.zip › Intermediate/intermediate 72.jpg]

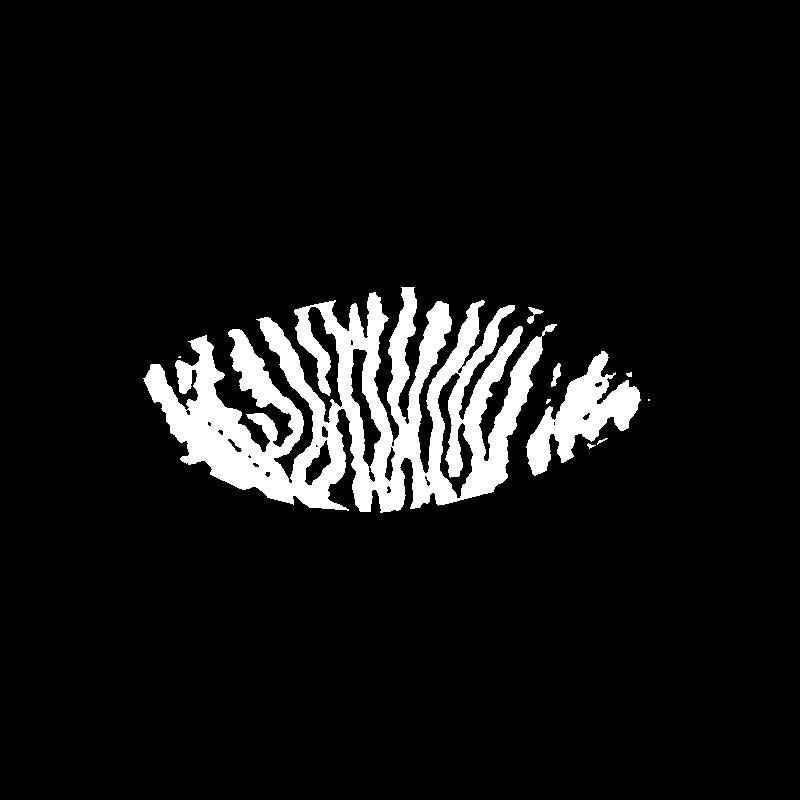

Supplement: S1 Raw images — (ZIP) [file pone.0270473.s008.zip › Intermediate/intermediate 73.jpg]

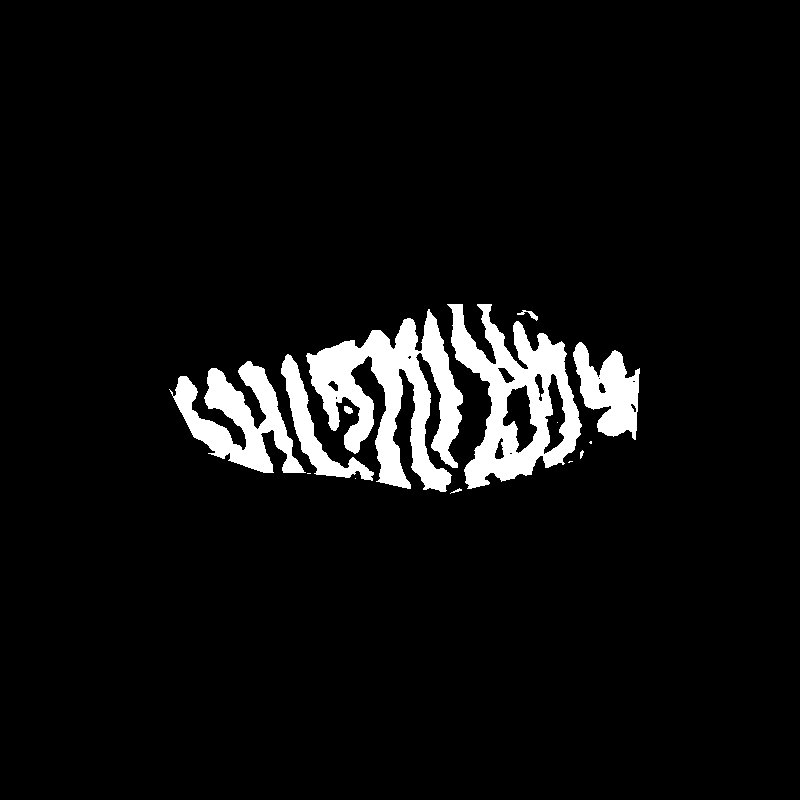

Supplement: S1 Raw images — (ZIP) [file pone.0270473.s008.zip › Intermediate/intermediate 74.jpg]

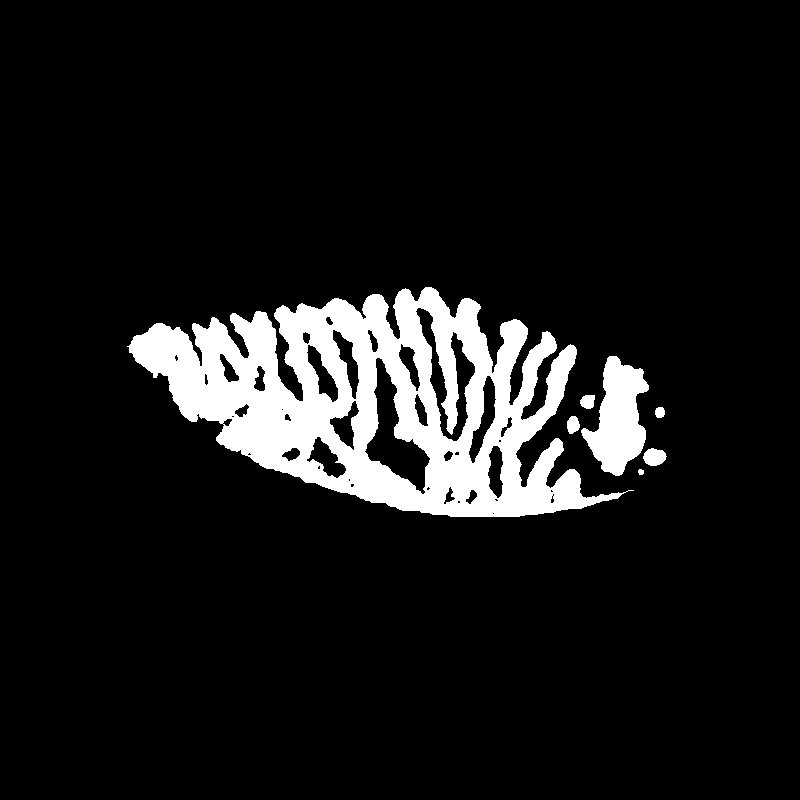

Supplement: S1 Raw images — (ZIP) [file pone.0270473.s008.zip › Intermediate/intermediate 8.jpg]

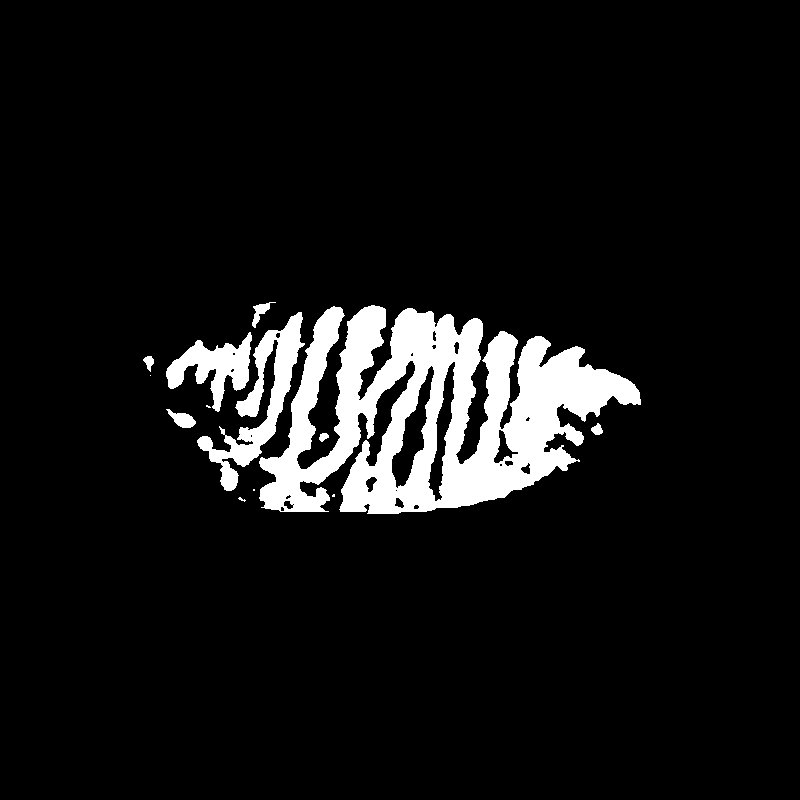

Supplement: S1 Raw images — (ZIP) [file pone.0270473.s008.zip › Intermediate/intermediate 9.jpg]

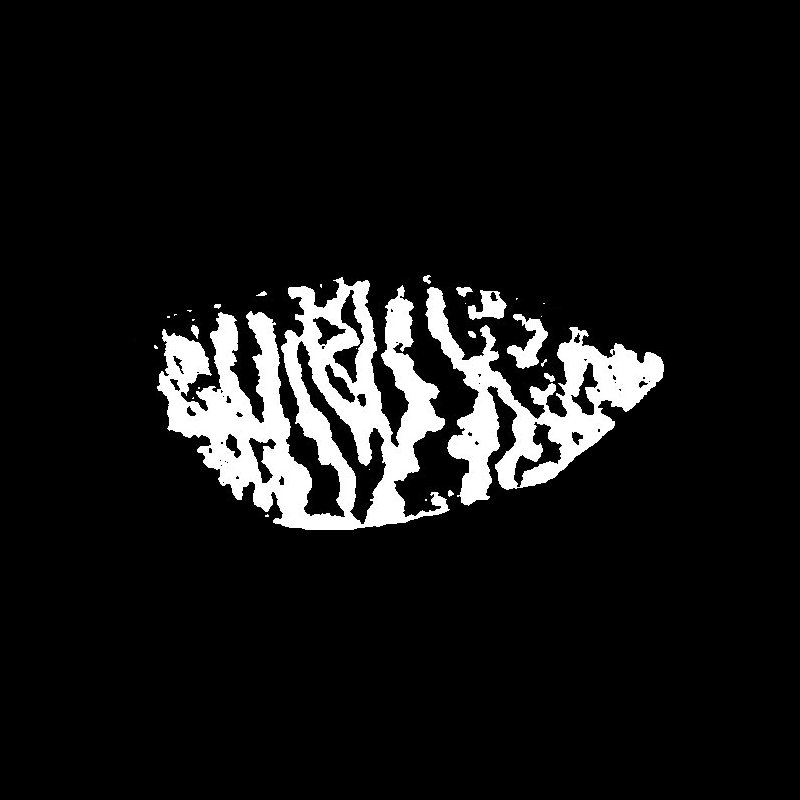

Supplement: S1 Raw images — (ZIP) [file pone.0270473.s008.zip › Unhealthy/unhealthy 1.jpg]

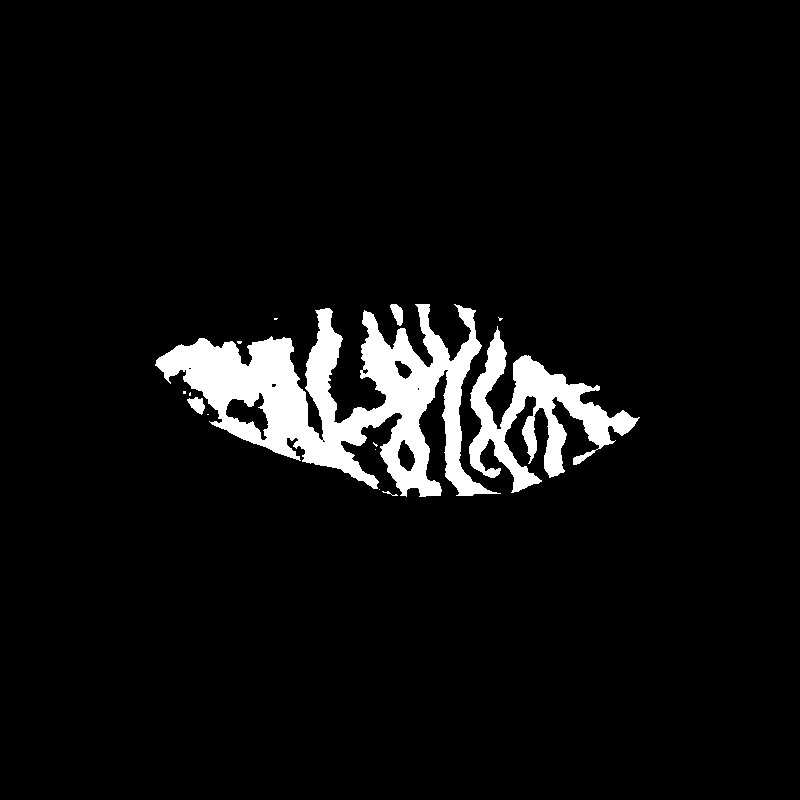

Supplement: S1 Raw images — (ZIP) [file pone.0270473.s008.zip › Unhealthy/unhealthy 10.jpg]
