# Supplementary material for: 2D Short-Time Fourier Transform for local morphological analysis of meibomian gland images
Source: PLoS One. 2022 Jun 24;17(6):e0270473. doi: 10.1371/journal.pone.0270473 (PMC9491703; doi:10.1371/journal.pone.0270473)
Supplement: Attachment — Submitted filename: Response to Reviewers.docx [file pone.0270473.s009.docx]

**Response to Reviewers**

**Response to the points raised by an Academic Editor:**

**(**Editor’s comments are in **red**. Author’s response is in **black)**

1. Please include ethics approval details for the current study. This used images from the study in The Ocular Surface 2020 (also not very details regarding ethics). Details on inclusion/exclusion criteria should also be included, and how many patients were examined should be stated. The number of images is noted in the manuscript, and that there were 2 images per person, but 'n' not provided.

New subsection named “Subjects” in “Materials and methods” was introduced where details on ethics approval, inclusion/exclusion criteria and number of examined patients were included.

2. Please give full details on the imaging techniques used for meibography. The authors used a custom built meibographer (based on the 2020 study). How does this compare to the clinically used (commercial) instruments, in terms of image resolution for of upper eyelid meibomian glands. Have the authors used their 2D Fourier transform analysis on routine clinical meibography images?

Standard IR retro-reflection meibograph is very simple device. Actually it is ordinary camera adapted to work with infrared light. The quality of the acquired images is determined just by the quality of the lens, resolution and dynamical range of the camera. The parameters of our system (resolution 20pix/mm and 8-bit dynamics) were sufficient to record a clear image of the basic structure of the Meibomian glands, that is, the feature we were interested during our study. We added new figure (Fig.1) which shows an exemplary raw meibographic image acquired by our system. As one can see it does not look much different from what is obtained with commercial instruments. We did not test our method on images acquired with such devices. It is certain that the quality of the images provided by these instruments will not be worse than ours. Therefore, we are confident that the described method will work also for that case.

More information on imaging technique used for meibography was introduced into “Meibographic images” section of “Materials and methods” as well as in dedicated “S1 Appendix. Meibomian gland images acquisition” supplementary document.

3. Can the individual meibomian gland analysis described here be practically applied in a clinical context - the analysis is complex, and it is not clear how this would be incorporated into a routine clinical examination.

A routine clinical examination can easily be extended to include proposed objective analysis of the morphological status. We remind that only the raw image of meibomian glands is sufficient to perform the described analysis. In the case of such an extended examination, the clinician would have access to both: a real picture of the glands, and to the corresponding morphometric maps. Of course, we do not expect the clinician to perform this analysis himself. Our method should be included into the imaging software. Most conveniently to imaging software of commercial meibograph (sort of module/plug-in). We hope we provide all the necessary technical details of the technique to make it easier to replicate it by software engineers.

We agree that the analysis is not trivial, but from the clinician's point of view, what is important is the way of interpreting the morphometric maps produced by our method. We have significantly expanded the "Discussion" section with additional information on how to read those maps so that the reader has a better understanding of their interpretation.

Another clinically useful feature of presented method is that morphometric maps can also be used to define and automatically estimate the values of new meibo-scores. We added new Fig.8 to show the potential definition of such score. Of course, the usefulness of any specific parameter determined this way in the clinical practice must be demonstrated by the future dedicated and systematic tests.

4. This is important to justify, noting the conclusion (line 316): "To conclude, our method for isolating individual morphometric components from the original Meibomian image can be of invaluable aid in the diagnostic process. It may help clinicians to see in which part of the eyelid disturbance is taking place and also to quantify it with a numerical value providing essentially insight into disease pathophysiology." The results presented do not really support the 'invaluable aid' comment and should be modified as such.

The new section “Conclusion” was introduced where we summarize our results in a broader way. The statement on 'invaluable aid' was softened.

5. Can the authors comment on associations between the eyelid meibography 'heatmaps' generated using the local analysis, and symptoms reported by the patients. Thank you.

The connection between the observed gland distortion and the patient’s symptoms was already recognized and described in the literature. We have cited appropriate papers in our manuscript [9-11, 14-15, 24-25, 32-34]. This observation was based on manual measurements of different morphological features. The aim of our work was not to search for a new correlation between gland’s morphological change and particular symptom, but to provide a tool for automatic and objective characterization of local gland distortion. This tool allows for quick and automatic mapping of defined morphological parameters in an objective manner, not requiring manual measurements. We hope that in the near future the systematic research using this tool may speed up and objectify the measurement of morphological features providing deeper understanding of the connection between gland distortion and various clinical measures.

We addressed this issue in several places in the significantly expanded "Discussion" section and in the "Conclusions" section.

6. A careful edit for English grammar and expression in the entire manuscript would improve the communication; there are sections that are not well expressed.

We have reviewed the manuscript to improve language usage. In addition to minor corrections, many new sentences appeared and old sentences have been rewritten to improve their readability without changing their meaning.

**Response to the points raised by Reviewer #1:**

**(**Referee’s comments are in **red**. Author’s response is in **black)**

1. Please include details of the original ethics approval, from which the images were obtained.

It would also be helpful to understand the general inclusion/exclusion criteria of the original study population

New subsection named “Subjects” in “Materials and methods” was introduced where details on ethics approval, inclusion/exclusion criteria and number of examined patients were included.

2. Materials and Methods – the meibomian gland images were graded 1-3 on two consecutive

days and the results were well correlated. However, it would be helpful if the authors could clarify how many researchers were involved in the grading, and how the images were classified if the grades assigned were different on the two days

Only one experienced optometrist was involved in the grading of meibomian images and the rating was done on two consecutive days. If the grades assigned were different on the two days, the images were reanalysed again to make a proper decision based on the presented criteria.

We have added the appropriate sentences in the section “Meibographic images” to better clarify this point.

3. Table 1 – please add confidence intervals or other statistical analyses to this table. Otherwise,

it is difficult to determine whether these proportions are significantly different from one another

95% Confidence intervals were added to the table. Details on their evaluation was also introduced into the Supplementary Materials document (S7 Appendix).

4. Line 211 belongs in the Materials and Methods section, but further detail needs to be added

regarding the “home-built meibographic imaging equipment” used. How does this compare to commercially available imaging tools

Standard IR retro-reflection meibograph is very simple device. Actually it is ordinary camera adapted to work with infrared light. The quality of the acquired images is determined just by the quality of the lens, resolution and dynamical range of the camera. The parameters of our system (resolution 20pix/mm and 8-bit dynamics) were sufficient to record a clear image of the basic structure of the Meibomian glands, that is, the feature we were interested during our study. We added new figure (Fig.1) which shows an exemplary raw meibographic image acquired by our system. As one can see it does not look much different from what is usually presented as obtained with commercial instruments. We did not test our method on images acquired with such devices. It is certain that the quality of the images provided by these instruments will not be worse than ours. Therefore, we are confident that the described method will work also for that case. Surely, with better equipment better results can be expected.

More information on imaging technique used for meibography was introduced into “Meibographic images” section of “Materials and methods” as well as in dedicated “S1 Appendix. Meibomian gland images acquisition” supplementary document.

5. Line 121 – the sentence beginning “In next section” is unnecessary

The sentence was removed

6. The manuscript should remove all personal pronouns e.g. line 121 “we”, line 132 “our”

Personal pronouns were removed

7. Line 316 – please soften the conclusion “invaluable aid” – this is too strong and not necessarily supported by the data

The new section “Conclusion” was introduced where we summarize our results in a broader way. The statement on 'invaluable aid' was softened.

8. Do the findings correlate with any ocular symptoms or other signs of dry eye?

The aim of our work was not to search for a correlation between gland’s morphological change and particular symptom, but to provide a tool for automatic and objective characterization of local gland distortion. Of course, the motivation for creating such a tool is a strong evidence that the meibomian gland morphological changes correlate with the ocular symptoms and signs of the dry eye. We have cited appropriate papers in our manuscript [9-11, 14-15, 24-25, 32-34]. The presented method allows for quick and automatic quantification of defined morphological parameters in an objective manner, not requiring manual measurements. While we do not provide any evidence for this, this logical sequence implies that the reported results should correlate with ocular symptoms.

We hope that in the near future the systematic research using this tool will provide such a proof and will confirm the clinical usefulness of the proposed approach.

9. What is the clinical utility of this analysis system? Is it something that can be easily

incorporated into clinical practice? What do the findings actually mean, since no associations are discussed relating the health of the glands and other signs and symptoms of dry eye?

Distortion of the glands is a well-known indicator of the health of the glands and it is considered to be a symptom of early stage dry eye disease. Our finding concerns the quantitative and automatic assessment of the degree of deformation of the glands. Additionally, morphological parameters are presented in the form of spatial color-coded maps.

A routine clinical examination can easily be extended to include the proposed objective analysis of the morphological status. We remind that in principle only the raw image of meibomian glands is sufficient to perform the described analysis. Of course, we do not expect the clinician to perform this analysis himself. Our method should be included into the imaging software. Most conveniently to imaging software of commercial meibograph (sort of module/plug-in). In the case of such an extended examination, the clinician would have access to both: a real picture of the glands, and to the corresponding morphometric maps. We have significantly expanded the "Discussion" section with additional information on how to read those maps so that the reader has a better understanding of their interpretation.

Another clinically useful feature of presented method is that morphometric maps can also be used to define and automatically estimate the values of new meibo-scores. We added new Fig.8 to show the potential definition of such score. Of course, the usefulness of any specific parameter determined this way in the clinical practice must be demonstrated by the future dedicated and systematic tests.

We have expanded the "Discussion" section with many additional descriptive sentences, hoping that the potential clinical utility will be better understood.

**Response to the points raised by Reviewer #2:**

**(**Referee’s comments are in **red**. Author’s response is in **black**.**)**

1. How was MG function scored?

The aim of our research was to develop a method for automatic and objective determination of local changes in the morphology of Meibomian glands. For such a task, scoring of the MG functions was not necessary. Therefore, the function of the MG and other ocular symptoms and signs of the dry eye disease were not scored. To make it clear, we've added a sentence to the "Meibographic images" section.

Of course, it would be very useful to determine the correlation between morphological changes and other features that determine the condition of the glands. This task is beyond the scope of our work. However, there is a strong evidence that the meibomian gland morphological changes correlate with the ocular symptoms and signs of the dry eye. We have cited appropriate papers in our manuscript [9-11, 14-15, 24-25, 32-34]. Therefore, we can hope that the parameters estimated by us may also be useful in diagnosis of the ocular symptoms and the image analysis method will make the procedure faster and more objective.

2. How user-friendly this method is?

Our meibography system is based on the usual retroreflective approach. It has been mounted on a Topcon SL-D701 slit lamp allowing the recording of meibographic images of the upper and lower eyelids during a routine eye examination. Image analysis method described in the article can be easily integrated into an image acquisition software. What is important for the clinical practice this method is fast and is not only user-friendly but also patient-friendly causing no discomfort during the procedure and providing the images which can be easily demonstrated to the patients. Information on this point was added to the conclusion section.

3. Adding 1 or 2 sample images to the manuscript will help readers better understand and visualize to what extent authors are referring to deformity of glands.

In the "Materials and methods" section, a new image has been added (Fig. 1), which shows the effect of image preprocessing and marks the areas considered by the optometrist as distorted.

4. Also, I would like to know about the complete tarsal area what was exposed to calculate MGs height. Height of MGs may differ with eversion specially for lower lids as we have less surface area.

In our work, the measure of distortion was strictly defined by derivatives of the orientation and the width of the glands and did not require knowledge of their length. Therefore, the height of the glands was not calculated. However, even if our approach focuses on the shape of the glands (rather than their dimensions) it was very important to expose the eyelid properly, especially in order to correctly image the distal areas of the eyelid. We used only the images of the best quality and presenting the largest area of the tarsal plate containing the Meibomian glands.

Although we have always collected a set of meibographic images of the upper and lower eyelids for each patient, we only used images of the upper eyelid to test our method. This decision was made only because of the larger surface of the upper eyelid on which the shape changes can be easier to visualized. In the further study the lower eyelids should be examined as well.

5. Good but do comment on usefullness of this method. What is the clinical utility of this technique?

As mentioned before, distortion of the glands is a well-known indicator of the health of the glands and it is considered to be a symptom of early stage dry eye disease. Therefore the detailed morphometric analysis is needed to establish what kind of changes are crucial in the proper diagnostic procedure. Our finding concerns the quantitative and automatic assessment of the degree of deformation of the glands. Additionally, morphological parameters are presented in the form of spatial color-coded maps. Compared to the other methods our algorithm allows to indicate subtle changes in the MG morphology, which is important for the proper follow up examinations.

A routine clinical examination can easily be extended to include proposed objective analysis of the morphological status. Our method should be included into the imaging software. Most conveniently to imaging software of commercial meibograph (sort of module/plug-in). Then, the clinician would have access to both: a real picture of the glands, and to the corresponding morphometric maps indicating where in the eyelid there is a specific morphological change (change in angle or change in width). We have added new sentences into the "Discussion" section on how to read those maps so that the reader has a better understanding of their interpretation.

Another clinically useful feature of presented method is that morphometric maps can also be used to define and automatically estimate the values of new meibo-scores. We added new Fig.8 to show the potential definition of such score. Of course, the usefulness of any specific parameter determined this way in the clinical practice must be demonstrated by the future dedicated and systematic tests.

We have significantly expanded the "Discussion" section and introduced additional information into “Conclusion” section, hoping that the potential clinical utility will be better understood.

6. Tortuosity is related more to the twisting or curved MGs. In literature, there are several methods to measure tortuosity and deformity. I would like to know more about the method authors have used and its reliability as well. Better to describe with sample images.

So far, all the recognized in the literature methods to measure tortuosity and deformity of the meibomian gland consider only individual the most affected glands as a representation of the entire eyelid area. The simplest approach is to simply count the number of distortion events. In the new Fig.1 we have marked, for example, the regions considered by the optometrist to be distorted. Here we have limited ourselves to only two features: change of gland direction and glands narrowing. With our method, the values of these features (or their derivatives) can be estimated automatically for each position on the eyelid. This not only makes the analysis procedure more objective and faster, but also makes it possible to define new meibo-scores. An example of two such scores that quantify the percentage of the eyelid area covered by deformed regions of the glands is shown in the new Fig. 8.

7. There are various ways to quantify Meibomian gland images particularly three-dimensional Fourier domain OCT. How this 2D Fourier transform is better?

The method of 3D Fourier domain OCT is just another tool for collecting images of the gland structure. It is imaging technique, not an analytical technique. Here, the Fourier transformation is used to speed-up acquisition process. This imaging technique is much more sophisticated than simple retroflection of usual meibographs (after all it is OCT method). The data are better quality (although the field of view is rather small) and provide information also on the depth of the gland (instead of just its surface studied by usual retroreflection meibographs). So surely, 3D OCT tool provide a lot of high-quality data. But again, it does not provide ways for quantitative analysis. In order to extract information about any gland feature (e.g. drop-out volume or gland orientation), the data from 3D OCT should also be subjected to appropriate analysis. This is the area of application of our method. Our work does not involve new method for acquiring images (we used usual retroreflection method), but rather a new way of their analysis focused on automatic and objective quantification of glands distortion. This is where Fourier transform came in handy. We are confident that our method can be successfully used also to analyze data from 3D OCT instruments.
